# Continuous-Flow Asymmetric Photocatalysis for the Synthesis of Natural and Unnatural α‑Amino Acid Derivatives

**DOI:** 10.1021/acsorginorgau.6c00024

**Published:** 2026-05-06

**Authors:** Marcelo Straesser Franco, Rodrigo Costa e Silva, Rafael Alan Carvalho Souza, Eric Yoshitaka Lee, João Marcos Batista Junior, Julio Cezar Pastre

**Affiliations:** † Institute of Chemistry, 28132 Universidade Estadual de Campinas (UNICAMP), 13083-862, Campinas, São Paulo, Brazil; ‡ Institute of Science and Technology, Universidade Federal de São Paulo (UNIFESP), 12231-280, São José dos Campos, São Paulo, Brazil

**Keywords:** asymmetric photocatalysis, continuous-flow, α-amino acid derivatives, radical addition, enantioselective synthesis, chiral-at-metal rhodium catalyst

## Abstract

Enantiomerically enriched α-amino acids and their derivatives are important building blocks, widely used in the pharmaceutical industry, drug research, and materials science. Therefore, the development of straightforward and versatile synthetic methods remains highly desirable. Herein, we disclose a photochemical enantioselective addition of alkyl radicals to α-imino esters, mediated by a chiral-at-metal rhodium catalyst under continuous flow conditions. This strategy provides rapid and direct access to enantioenriched derivatives of both natural and unnatural α-amino acids, achieving yields of up to 75% and enantiomeric ratios of up to 1:99.

## Graphical abstract

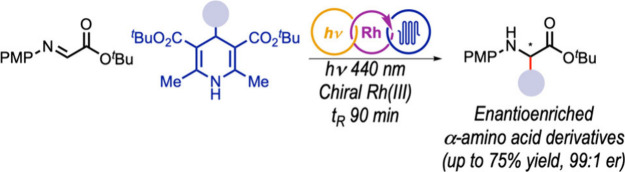

Chiral α-amino acids (α-AAs) and their derivatives constitute privileged building blocks, widely applied in the pharmaceutical industry, new drug research, catalysis and materials science.^1–5^ Furthermore, these compounds can be readily converted into diverse high-value enantioenriched architectures, underscoring the importance of efficient and broadly applicable methods for their synthesis.^6,7^

Over the past decade, the resurgence of visible light catalysis has provided a powerful platform for the activation of organic substrates. Coupled with advances in asymmetric photocatalysis, this strategy has opened new horizons for the direct, efficient, and sustainable synthesis of enantioenriched compounds. These developments have not only expanded the synthetic toolbox but also inspired new conceptual approaches for accessing molecular complexity.^8–12^

In parallel, continuous flow chemistry has emerged as a transformative enabling technology for photochemistry. Flow reactors offer superior photon transport, enhanced reproducibility, and straightforward scalability, thereby overcoming key limitations of traditional batch systems. As a result, photocatalytic processes under continuous flow conditions have become increasingly recognized as the method of choice for translating light-driven reactions into practical synthetic applications.^13,14^

To date, only a few batch-based enantioselective photocatalytic approaches have been reported for the asymmetric synthesis of α-amino acids, most of which rely on dual-catalysis strategies to achieve stereocontrolled addition of carbon-centered radicals or nucleophiles to glycine or dehydroalanine derivatives (Figure 1A).^15–19^ Notably, to the best of our knowledge, no method has yet been established for the synthesis of α-AAs under continuous flow conditions.

**Figure 1:**
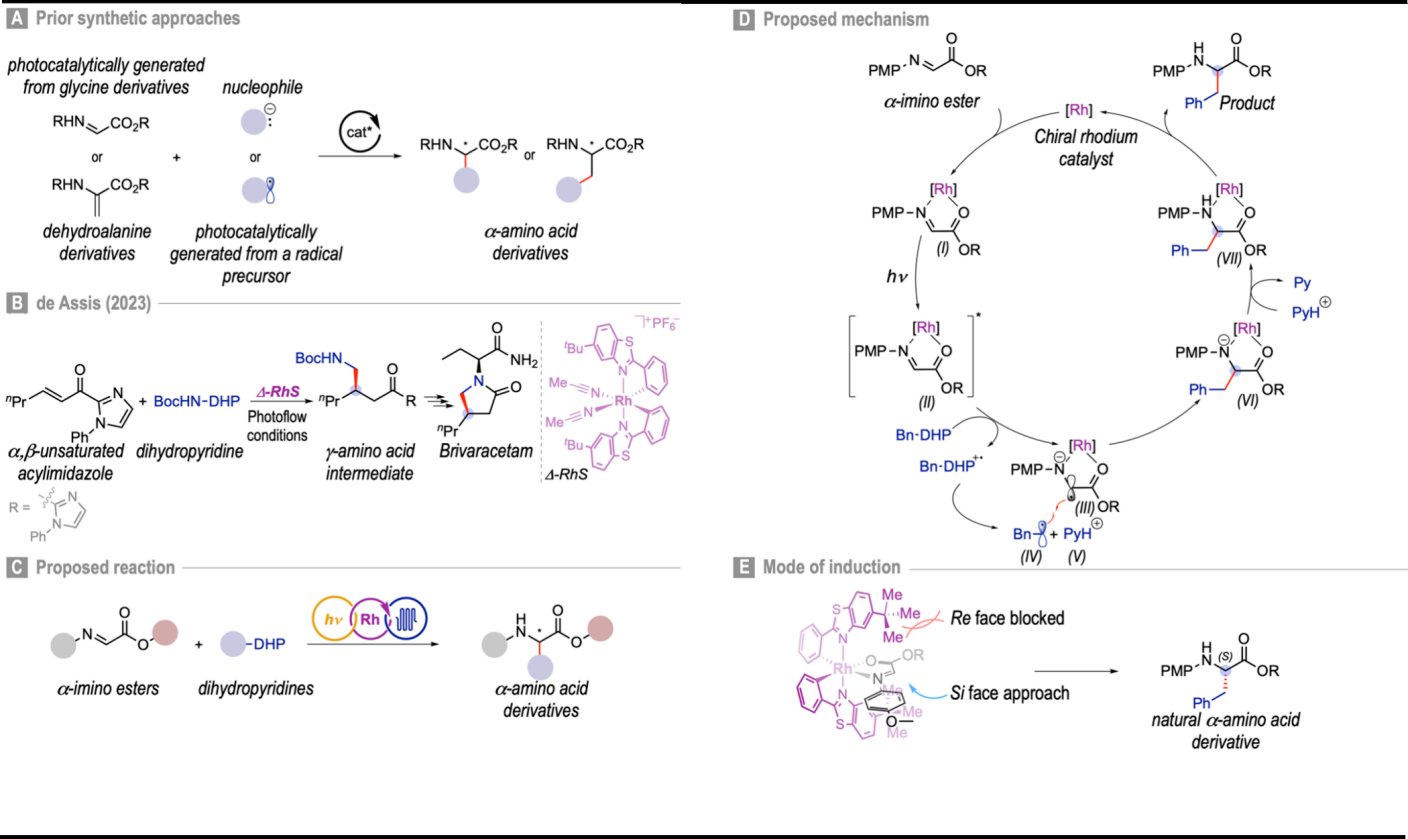
Enantioselective photocatalytic synthesis of α-AAs, proposed mechanism and predicted induction mode. DHP = dihydropyridine; PMP = *p*-methoxyphenyl.

Recently, de Assis and co-workers reported an asymmetric photocatalytic methodology mediated by a chiral-at-metal rhodium­(III) complex (**Δ-RhS**) under continuous-flow conditions for the synthesis of a γ-amino acid intermediate, which was subsequently applied to the preparation of brivaracetam, a drug used for the treatment of epilepsy (Figure 1B).^20^ This chiral-at-metal rhodium catalyst, pioneered by Meggers’s group, has emerged as a powerful platform in asymmetric photocatalysis, typically applied to substrates bearing coordinating auxiliaries.^21^ Inspired by this precedent, we envisioned an enantioselective photocatalytic strategy in a continuous flow regime for the stereocontrolled synthesis of both natural and unnatural α-amino acid derivatives, mediated by chiral bifunctional rhodium­(III) catalysts (Figure 1C). In contrast, our design employs an α-imino ester that inherently functions as a bidentate ligand.

In the proposed design, the chiral rhodium Lewis acid catalyst coordinates with the α-imino ester to form a photoactivatable complex (**I**). Upon irradiation, this complex (**II**) undergoes single-electron transfer (SET) with the dihydropyridine (**Bn-DHP**), generating the Rh-substrate radical anion (**III**), the alkyl radical (**IV**), and pyridine (**V**). The alkyl radical then engages in a stereocontrolled radical–radical coupling with intermediate (**III**) to furnish species (**VI**). Subsequent protonation affords the Rh-bound product (**VII**), which is released to regenerate the rhodium catalyst, thereby closing the catalytic cycle (Figure 1D).

Based on previously proposed induction models,^21,22^ we anticipated that with the **Λ-RhS** catalyst, radical–radical coupling would proceed through the *Si* face of the prochiral substrate, as approach from the *Re* face is sterically hindered by the *tert*-butyl groups of the catalyst (Figure 1E).

Guided by this design, we set out to develop the asymmetric photocatalytic synthesis of α-amino acid derivatives under continuous flow conditions. As a model system, we selected the α-imino ester (**1a**, 1 equiv) and the dihydropyridine (DHP, **2a**, 1.2 equiv) in combination with the chiral rhodium complex as catalyst (**Λ-RhS**, 10 mol %).

To our delight, under preliminary conditions (30 min residence time at 25 °C under blue-light irradiation – 440 nm), ethyl (4-methoxyphenyl)­phenylalaninate **3aa** was obtained in 80% yield with an enantiomeric ratio of 22:78 (Table 1, entry 1). This promising outcome demonstrated the potential of the system while revealing the need for further optimization to improve stereocontrol.

**Table 1:**
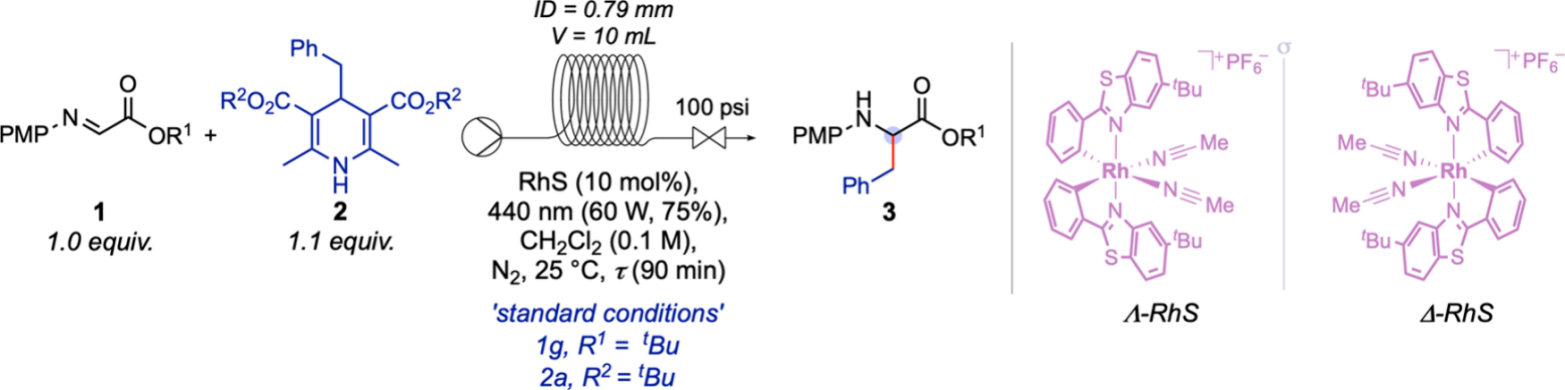
Reaction Optimization and Effect of Deviations from the Standard Conditions^k^

Entry [^a^]	experimental conditions [^b^]	compound **3**	yield (%) [^c^]	er [^d^]
1	**1a** (R^1^ = Et) with **2a** (R^2^ = ^*t*^Bu), τ = 30 min	**3aa**	80	22:78 [^e^]
2	**1a** (R^1^ = Et) with **2ab** (R^2^ = Et), τ = 180 min	**3aa**	26 [^g^]	23:77 [^e^]
3	**1a** (R^1^ = Et) with **2a** (R^2^ = ^*t*^Bu) in absence of catalyst, τ = 30 min	**3aa**	22	50:50
4	**1b** (R^1^ = Et and 3,4-DMP instead of PMP) with **2a** (R^2^ = ^*t*^Bu), τ = 30 min	**3ba**	61	29:71 [^e^]
5	**1c** (R^1^ = Et and 2,4-DMP instead of PMP) with **2a** (R^2^ = ^*t*^Bu), τ = 30 min	**3ca**	45	47:53 [^e^]
6	**1d** (R^1^ = ^*i*^Pr) with **2a** (R^2^ = ^*t*^Bu), τ = 30 min	**3da**	75	15:85 [^e^]
7	**1e** (R^1^ = ^*i*^Bu) with **2a** (R^2^ = ^*t*^Bu), τ = 30 min	**3ea**	64	15:85 [^e^]
8	**1f** (R^1^ = cyclohexyl) with **2a** (R^2^ = ^*t*^Bu), τ = 30 min	**3fa**	34	20:80 [^e^]
9	**1g** (R^1^ = ^*t*^Bu) with **2a** (R^2^ = ^*t*^Bu), τ = 30 min	**3ga**	35	93:7 [^f^]
10	**1g** (R^1^ = ^*t*^Bu) with **2a** (R^2^ = ^*t*^Bu) and 5 mol % of catalyst, τ = 30 min	**3ga**	16	13:87 [^e^]
11	**1g** (R^1^ = ^*t*^Bu) with **2a** (R^2^ = ^*t*^Bu) at – 20 °C, τ = 30 min	**3ga**	21	93:7 [^f^]
12	**1g** with **2a** and τ = 90 min	**3ga**	75	93:7 [^f^]
13	**1g** with **2a**; no catalyst	**3ga**	4 [^g^]	50:50
14	**1g** with **2a**; no light	**3ga**	0	-
15	**1g** with **2a**; addition of 3 equiv of TEMPO	**3ga**	<1 [^g^]	-
16	**1g** with **2a**; under air [^h^]	**3ga**	56	92:8 [^f^]
17	**1g** (1.5 equiv), **2a** (1.0 equiv)	**3ga**	74 [^i^]	14:86 [^e^]
18	**1g** with **2a**; 120 min, batch, rt [^j^]	**3ga**	60	14:86^e^

a All reactions were performed in a commercially available Vaportec R-series device equipped with a UV-150 photoreactor.

b Experimental conditions: **1** (1.0 equiv., 0.1 M in CH_2_Cl_2_), **2** (1.1 equiv), chiral rhodium catalyst (D-RhS or Λ-RhS, 10 mol %), visible light (440 nm, 60 W, 75% power), N_2_ atmosphere, 25 °C, flow rate (0.111 mL·min^–1^), residence time (*t*, 90 min).

c Isolated yield based on **1**.

d er: Enantiomeric ratio – Determined by chiral HPLC analysis.

e Used the Λ-RhS catalyst.

f Used the D-RhS catalyst.

g Yield was determined via ^1^H NMR by using 1,3,5-Trimethoxybenzene as internal standard.

h Nondegassed solvent.

i Isolated yield based on **2a**.

j The reaction was performed in batch mode, using a Schlenk tube, under visible-light irradiation (Kessil 440 nm, 40 W, 75% power). DMP = dimethoxyphenyl.

k See Supporting Information for more details.

Subsequent studies systematically examined the influence of substrate structure, catalyst loading, temperature, and residence time (Table 1; see the Supporting Information for the full range of conditions tested). Employing the Hantzsch ester **2ab** (R^2^ = Et), even with an extended residence time of 180 min, furnished **3aa** in only 26% yield and without improvement in selectivity (entry 2), demonstrating the superior efficiency of DHP **2a** (R^2^ = ^*t*^Bu) in alkyl radical transfer.^23,24^ However, under the initial reaction conditions, a significant background reaction was observed, with product formation occurring in the absence of the catalyst (22%, entry 3).

Modification of the nitrogen protecting group proved unproductive: replacement of PMP with 3,4-dimethoxy (entry 4) or 2,4-dimethoxy substituents (entry 5) failed to improve enantioselectivity and led to diminished yields (61% and 45%, respectively), indicating a critical role for PMP in catalyst–substrate recognition.

Variation of the ester substituent had a more pronounced impact. The isopropyl and isobutyl esters **1d** and **1e** (entries 6 and 7, respectively) afforded the corresponding products **3da** and **3ea** in yields of 75% and 64%, respectively, with improved enantioselectivity (15:85 er for both), indicating that moderate steric bulk at the imino ester enhances stereocontrol. A similar trend was observed for the cyclohexyl ester **1f** (entry 8; 20:80 er), albeit with diminished yield (34%). In contrast, the more sterically demanding *tert*-butyl ester **1g** (entry 9) provided excellent enantioselectivity (97:3 er), while delivering a reduced yield (35%).

These results suggest that the reaction performance is sensitive to the structural match between the chiral rhodium catalyst and the coordinated α-imino ester. Variations in steric and electronic properties likely affect the geometry of the catalyst–substrate complex, thereby influencing the efficiency of the stereocontrol.

Further optimization with the *tert*-butyl ester **1g** showed that reducing the catalyst loading to 5 mol % decreased both yield and enantioselectivity (16% yield, 87:13 er, entry 10). Lowering the temperature to – 20 °C (entry 11) preserved enantioselectivity (93:7 er) but resulted in a lower yield (21%). Ultimately, conducting the reaction at 25 °C with an extended residence time (τ = 90 min) afforded the target product in 75% yield and 93:7 er, thereby establishing a photoflow system that enables the enantioselective addition of a benzylic radical to α-imino esters under visible-light irradiation, using 10 mol % RhS, a slight excess of **2a** (1.1 equiv), and a 0.1 M solution in CH_2_Cl_2_ (entry 12).

Control experiments confirmed the essential role of both the photocatalyst and light: omission of the catalyst afforded only 4% of racemic product **3ga**, while in the absence of irradiation, no reaction occurred (entries 13 and 14). These findings highlight the importance of the *tert*-butyl substituent on the α-imino ester **1g** in suppressing background reactivity (consistent with reduced electrophilicity and increased steric hindrance of the *tert*-butyl α-imino ester), as well as the necessity of the catalyst for enantioselective activation and the photodependent nature of the transformation.

The addition of 3 equiv of TEMPO ((2,2,6,6-tetramethylpiperidin-1-yl)­oxyl) completely suppressed the reactivity (entry 15), consistent with a radical pathway. Performing the reaction under air significantly diminished efficiency (56% yield) but maintained stereocontrol (92:8 er, entry 16). Notably, altering the standard stoichiometry from **1g** (1.0 equiv) and **2a** (1.1 equiv) to **1g** (1.5 equiv) and **2a** (1.0 equiv) resulted in a significant erosion of enantioselectivity (14:86 er, entry 17), indicating the sensitivity of the system to reagent balance.

Finally, switching from continuous flow to batch operation also resulted in diminished stereocontrol and yield, even when employing a slightly longer reaction time of 120 min (60% yield, 14:86 er, entry 18). This effect can be attributed to the intrinsic difficulty of maintaining a homogeneous photon flux and precise temperature control under batch conditions, highlighting the importance of careful parameter regulation in suppressing background reactions and improving selectivity. In this context, enabling technologies such as continuous-flow reactors are crucial to ensure uniform photon flux and accurate temperature control, thereby enhancing both selectivity and reproducibility.^25,26^

With the optimized conditions in hand (Table 1, entry 12), we next evaluated the scope of the transformation (Figure 2). The catalytic system proved broadly applicable, delivering both natural and unnatural α-amino acid derivatives in yields of up to 75% and with enantioselectivities as high as 1:99. Remarkably, the tryptophan analogue **3gg** was obtained in 58% yield and 1:99 er, representing the highest level of stereocontrol observed in our study.

**Figure 2:**
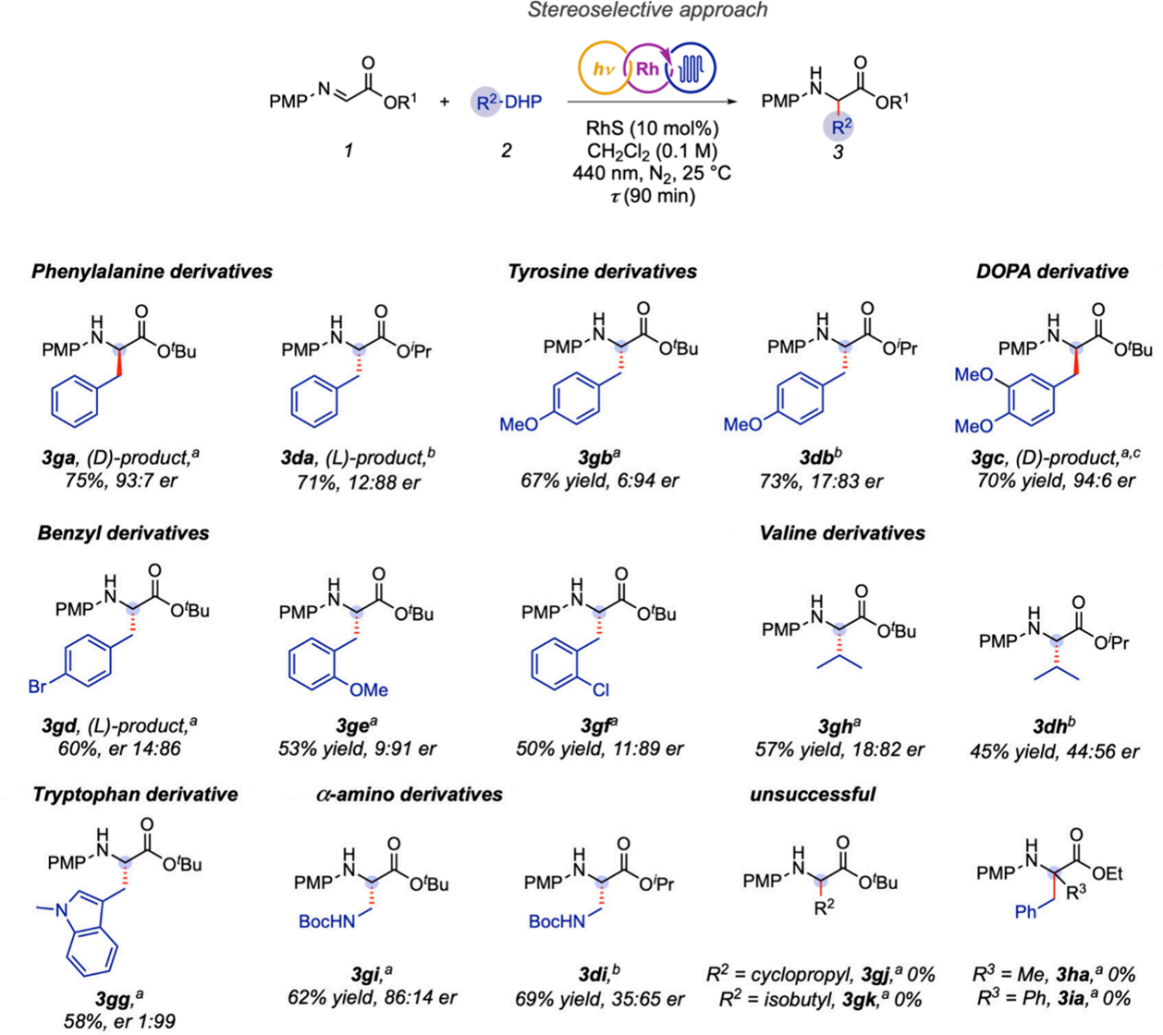
Substrate scope of the enantioselective RhS-catalyzed photochemical Giese-type alkylation of α-imino esters under continuous flow conditions. [a] Standard conditions: **1g** (or **1h**, or **1i**) (1.0 equiv., 0.1 M in CH_2_Cl_2_), **2** (1.1 equiv), chiral rhodium catalyst (Δ-RhS or Λ-RhS, 10 mol %), visible light (440 nm, 60 W, 75% power), N_2_ atmosphere, 25 °C, flow rate (0.111 mL·min^–1^), residence time (τ, 90 min). [b] Conditions: **1d** (1.0 equiv., 0.1 M in CH_2_Cl_2_), **2** (1.1 equiv), chiral rhodium catalyst (Λ-RhS, 5 mol %), visible light (440 nm, 60 W, 75% power), N_2_ atmosphere, – 20 °C, flow rate (0.333 mL·min^–1^), residence time (τ, 30 min). [c] τ, 180 min. Note: The opposite enantiomers observed for **3ga** and **3gc** result from the use of the Δ-RhS catalyst; in all other cases, the Λ-RhS enantiomer was employed. See Table 1 for the conditions and the Supporting Information for details.

Electronic effects had a decisive impact on reactivity and selectivity. Precursors generating stabilized radicals, such as 4-methoxy and 3,4-dimethoxy benzyl-DHPs, afforded the biologically relevant **tyrosine** and **DOPA** derivatives in good yields and with high enantioselectivity (**3gb**, 67% yield, 6:94 er; **3gc**, 70% yield, 94:6 er). In contrast, the electron-withdrawing 4-bromo substituent gave reduced yield and lower selectivity (**3gd**, 60% yield, 14:86 er), underscoring the difficulty of engaging less-stabilized radical intermediates.

Steric effects were equally evident. *Ortho*-substituted derivatives (**3ge**, **3gf**) furnished reduced yields (53 and 50%, respectively) and a modest decrease in enantioselectivity (9:91 er and 11:89 er, respectively), consistent with steric congestion near the radical site impairing both reactivity and stereocontrol. Similarly, the use of DHP **2h**, a precursor of the isopropyl radical, proved challenging, delivering the **valine** derivative **3gh** in 57% yield and with lower enantioselectivity (18:82 er). Notably, the Boc-protected α-amino derivative **3gi** was obtained in 62% yield with moderate enantioselectivity (86:14 er), indicating that heteroatom-substituted radicals are tolerated, albeit with partially diminished stereocontrol. These results highlight the limiting influence of steric and electronic factors on both efficiency and stereocontrol in this transformation.

We also examined an alternative set of conditions previously optimized for the isopropyl α-imino ester (**1d**) at – 20 °C (see Supporting Information). However, no improvements in yield or enantioselectivity were observed for the corresponding products (Figure 2, **3da**, **3db**, **3dh**, **3di**).

Finally, cyclopropyl- and isobutyl-derived dihydropyridines failed to undergo productive conversion (**3gj** and **3gk**), highlighting limitations with highly strained or poorly stabilized radicals. Similarly, α-substituted imino esters (Me, Ph) showed no reactivity (**3ha** and **3ia**), likely due to steric hindrance that impairs catalyst coordination and radical addition. Collectively, these results define the scope and limitations of this catalytic system and provide insight into the steric and electronic factors governing enantioselective radical–radical coupling under photoflow conditions.

In addition, the use of the PMP protecting group is strategic, as its removal from product **3ga** has been reported, enabling subsequent conversion into the peptide building block **4ga** in good yield and without loss of enantiopurity.^19^ This transformation establishes a formal synthesis of **4ga** from **3ga** (Figure 3A), thereby further highlighting the synthetic utility of the developed method.

**Figure 3:**
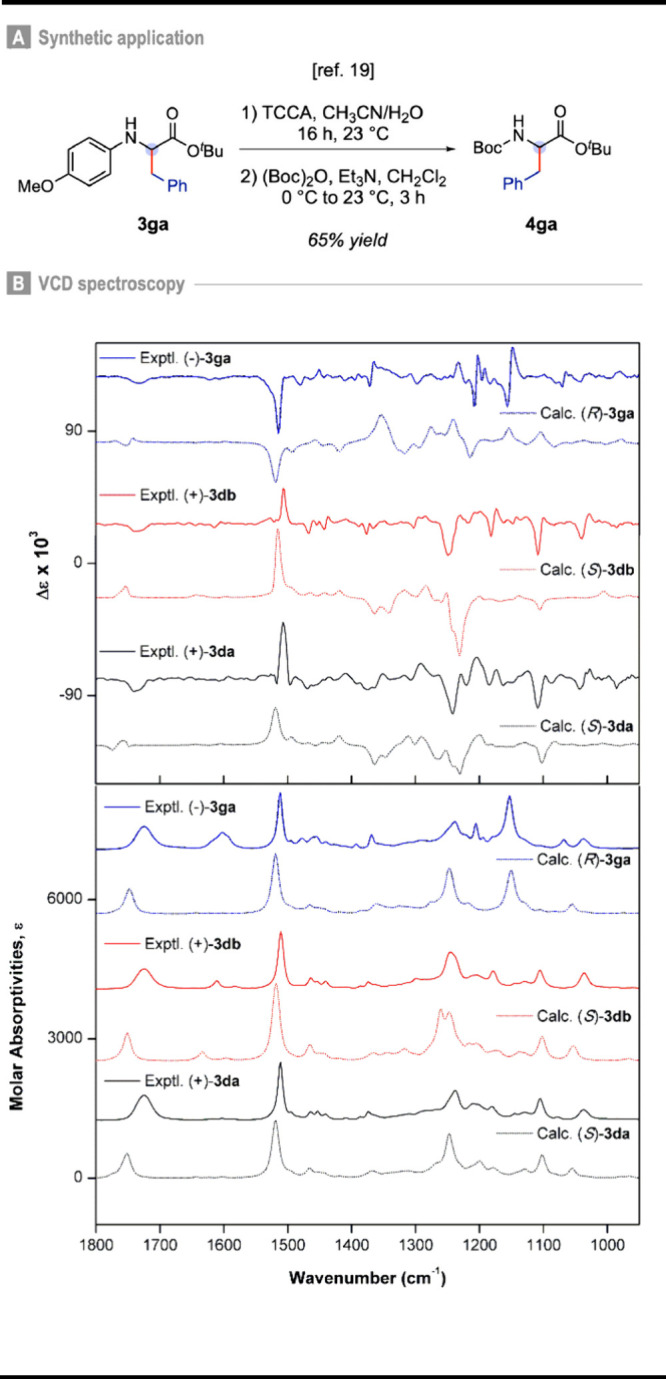
A) Synthetic application. B) Comparisons of experimental and calculated IR (lower panel) and VCD (upper panel) spectra for **3da**, **3db**, and **3ga**. Experiments recorded in CDCl_3_ and calculations performed at the B3PW91/PCM­(CHCl_3_)/6–311G­(d,p) level.

In order to assign the absolute configuration of compounds **3ga** {[α]_D_ ^24^ = – 10.1 (*c* = 0.05, CHCl_3_)}, **3da** {[α]_D_ ^23^ = +12.8 (*c* = 0.05, CHCl_3_)}, and **3db** {[α]_D_ ^22^ = +16.4 (*c* = 0.05, CHCl_3_)} and to confirm the predicted induction model (Figure 1E), a combination of vibrational circular dichroism (VCD) spectroscopy and DFT calculations was employed (Figure 3B). VCD arises from the differential absorption of left- and right-circularly polarized IR radiation by a chiral molecule during a vibrational transition. The assignment of absolute configurations by means of VCD is based mainly on the comparison of experimental data with theoretical spectra simulated for an arbitrarily chosen configuration using DFT.^27^ Over the last decades, VCD has been established as a reliable tool to determine the absolute configurations of synthetic molecular scaffolds of varying structural complexity without requiring any specific UV/vis chromophores, chemical derivatization or growth of suitable crystals.^28^ Therefore, the good correlation between the experimental IR and VCD spectra recorded in CDCl_3_ for **3da** and **3db** with the calculated [B3PW91/PCM­(CHCl_3_)/6–311G­(d,p)] data for the (*S*) enantiomer, as well as those of **3ga** with the calculated data for the (*R*) enantiomer led to the assignment of both (+)-**3da** and (+)-**3db** as (*S*) and (−)-**3ga** as (*R*). Taken together, these results corroborate the proposed induction model (Figure 1E).

In conclusion, we have developed an innovative enantioselective photocatalytic method in a continuous flow regime that enables the rapid and direct synthesis of a broad range of enantioenriched natural and unnatural α-amino acid derivatives, as well as key intermediates for active pharmaceutical ingredients (APIs). This strategy, based on the addition of alkyl radicals generated from dihydropyridines to prochiral α-imino esters mediated by bifunctional rhodium­(III) catalysts, expands the scope of asymmetric photocatalysis in flow and provides streamlined access to biologically active compounds and pharmaceutical intermediates.

## Supplementary Material



## Data Availability

The data underlying this study are available in the published article and its Supporting Information.

## Notes

Experimental procedures, experimental setup, optimization data, and characterization details for the α-amino acid derivatives (PDF)
