# Supplementary material for: Continuous-Flow Asymmetric Photocatalysis for the Synthesis of Natural and Unnatural α‑Amino Acid Derivatives
Source: ACS Org Inorg Au. 2026 May 6;6(3):282–7. doi: 10.1021/acsorginorgau.6c00024 (PMC13237600; doi:10.1021/acsorginorgau.6c00024)
Supplement: Supplementary file 1 [file gg6c00024_si_001.pdf]

Supplementary Information

Continuous Flow Asymmetric Photocatalysis for the Synthesis of Natural and Unnatural α-Amino Acid Derivatives

Marcelo Straesser Franco, Rodrigo Costa e Silva, Rafael Alan Carvalho Souza, Eric Yoshitaka Lee, João Marcos Batista Junior, Julio Cezar Pastre*

[a] Institute of Chemistry, Universidade Estadual de Campinas (UNICAMP), 13083-862, Brazil
[b] Institute of Science and Technology, Federal University of São Paulo (UNIFESP), 12231-280, Brazil

*Corresponding author. E-mail: jpastre@unicamp.br

SUPPLEMENTARY INFORMATION

Table of Contents

I. GENERAL REMARKS 3
II. VIBRATIONAL CIRCULAR DICHROISM (VCD) SPECTROSCOPY 4
III. PHOTOFLOW SETUP 5
IV. BATCH REACTION SETUP 7
V. SYNTHESIS OF SUBSTRATES 7
VI.I Procedure A: Synthesis of substrates α-imino ester 1 8
VI.II Procedure B: Synthesis of substrate α-imino ester 1g 8
VI.III Procedure C: Synthesis of substrate α-imino ester 1h 9
VI.IV Procedure D: Synthesis of substrate α-imino ester 1i 9
VI.V Procedure E: Synthesis of DHP 2 10
VI.VI General procedure F: Preparation of compound 3ga 11
VII. REACTION OPTIMIZATION TABLES AND CONTROL EXPERIMENTS 11
VIII. ROBUSTNESS EXPERIMENTAL 16
IX. CHARACTERIZATION DATA 17
X - REFERENCE 37
XI. ^{1}H and ^{13}C{^{1}H} NMR SPECTRA 38
XII. CHROMATOGRAMS 56

SUPPLEMENTARY INFORMATION

I. GENERAL REMARKS

The starting materials and reagents were obtained from commercial suppliers and used without further purification unless stated otherwise. Solvents of technical grade were purified via distillation prior to use (hexane, ethyl acetate, dichloromethane, and methanol), and solvents of PA quality were used without further purification.

Product isolation was performed on an automated Biotage® Isolera One system using Biotage® SNAP Ultra 10 g, 25 g, or 50 g flash chromatography cartridges with Merck silica gel 60 (230-400 mesh) as the stationary phase, employing an appropriate solvent system for elution. Thin-layer chromatography (TLC) analysis was performed using silica on aluminium foils TLC plates (F254, Supelco Sigma-Aldrich®) with visualization under ultraviolet light (254 nm) and/or by immersion in vanillin or potassium permanganate developing solution, followed by heating the plate to stain the spots.

NMR spectra were recorded at various field strengths, as indicated, using Bruker 300, Bruker Avance 400, Bruker Avance 500, or Bruker Avance 600 spectrometers. Data are reported as follows: chemical shift (δ), multiplicity, coupling constant (*J*) and integrated intensity. ^{1}H NMR spectra are reported in parts per million (ppm), referenced to the $CDCl_3$ residual solvent peak (7.26 ppm) or tetramethylsilane (TMS) peak (0.00 ppm) and all $^{13}C\{^{1}H\}$ NMR spectra are reported in ppm relative to $CDCl_3$ residual solvent peak (77.16 ppm), unless stated otherwise. In the ^{1}H NMR spectra, multiplicity of peaks are described as singlets (s), doublets (d), doublet of doublets (dd), doublet of doublets of doublets (ddd), triplets (t), doublet of triplets (dt), and multiplets (m). Coupling constants (*J*) were reported to the nearest 0.4 Hz. 1,3,5-Trimethoxybenzene was used as internal standard for the determination of chemical yields by ^{1}H NMR. NMR data was processed using MestReNova 14 software package. Known products were characterized by comparing to the corresponding ^{1}H NMR and $^{13}C\{^{1}H\}$ NMR from literature.

Infrared spectra (IR) were acquired using espectrometer Thermo Scientific Nicolet IS5, using module Thermo Scientific ID3 ATR (ZnSe) and are expressed in cm^{-1}.

High resolution mass spectra (HRMS) were collected on an Orbitrap Thermo QExactive Mass Spectrometer.

High-Performance Liquid Chromatography (HPLC) analysis was performed on an Agilent 1260 Infinity II LC system with a DAD detector equipped with Daicel Chiralpak® chiral columns as stationary phase and hexanes:^{i}PrOH mixtures as mobile phase.

Optical rotation (OR) was measured in chloroform, at 589 nm, on a Jasco P2000 polarimeter equipped with a sodium lamp using a cylindrical glass sample cell of 1 dm pathlength and 10 mm ID. OR values were reported as a mean of 3–5 measurement blocks of 5 repetitions each.

IR and VCD experimental spectra were recorded simultaneously with a BioTools dual-PEM ChiralIR-2X FT-VCD spectrometer using a resolution of 4 cm^{-1} and a collection time of 15 h. The

optimum retardation of the ZnSe photoelastic modulators (PEMs) was set at 1400 cm^{-1}. The IR and VCD spectra of **3da**, **3db** and **3ga** were recorded in $CDCl_3$ solutions (7.0–9.0 mg in 180 μL) in a BaF_2 cell with 100 μm path length. Minor instrumental baseline offsets were eliminated by subtracting the VCD spectra of the solvent recorded under identical conditions.

II. VIBRATIONAL CIRCULAR DICHROISM (VCD) SPECTROSCOPY

To assign the absolute configuration of compounds **3da** {$[a]_D^{23}$ = +12.8 (*c* = 0.05, $CHCl_3$)}, **3db** {$[a]_D^{22}$ = +16.4 (*c* = 0.05, $CHCl_3$)}, and **3ga** {$[a]_D^{24}$ = –10.1 (*c* = 0.05, $CHCl_3$)} a combination of VCD spectroscopy and DFT calculations was used.[1] The good correlation between the experimental IR and VCD spectra recorded in $CDCl_3$ for **3da** and **3db** with the calculated data for the (*S*) enantiomer, as well as between IR and VCD spectra recorded in $CDCl_3$ for **3ga** with the calculated data for the (*R*) enantiomer led to the assignment of both (+)-**3da** and (+)-**3db** as (*S*) and (–)-**3ga** as (*R*) (**Figure S1**).

The conformational searches of compounds **3da**, **3db** and **3ga** were carried out at the molecular mechanics level of theory employing the MMFF94 force field (7-8 kcal mol^{-1} energy window) incorporated in ComputeVOA software (BioTools, Inc.). The DFT calculations were carried out at 298 K in chloroform solutions using the polarizable continuum model (PCM) in its integral equation formalism version (IEFPCM), incorporated in Gaussian 09 software.[2] The (*S*) configuration was arbitrarily chosen for **3da** and **3ga**, while the (*R*) configuration was chosen for **3db**. The VCD properties of their enantiomers were obtained by multiplying the calculated data by (-1). Initially, 156, 334, and 60 conformers were identified for **3da**, **3db** and **3ga**, respectively. The conformers of **3da** and **3db** were first geometry optimized at the B3LYP/PCM($CHCl_3$)/6-31G(d) level. After the removal of duplicates, 137 and 147 conformers of **3da** and **3db**, respectively, were reoptimized at the B3PW91/PCM($CHCl_3$)/6-311G(d,p) level. As for **3ga**, the 60 conformers identified in the conformational search were geometry optimized directly at the B3PW91/PCM($CHCl_3$)/6-311G(d,p) level. The lowest-energy conformers contributing with ≥1% of the Boltzmann distribution (29 conformers, rel E. ≤ 1.0 kcal mol^{-1} for **3da**; 31 conformers, rel E. ≤ 1.39 kcal mol^{-1} for **3db;** 21 conformers, rel E. ≤ 1.27 kcal mol^{-1} for **3ga**) were selected for IR and VCD spectral calculations, which were carried out at the B3PW91/PCM($CHCl_3$)/6-311G(d,p) level. IR and VCD spectra were created using dipole and rotational strengths from Gaussian, which were converted into molar absorptivity (M^{-1} cm^{-1}). Each spectrum was plotted as a sum of Lorentzian bands with half-widths at half-maximum (HWHM) of 6 cm^{-1}. The calculated wavenumbers were multiplied with a scaling factor of 0.98 and the Boltzmann-average-composite IR and VCD spectra were plotted using Origin software.

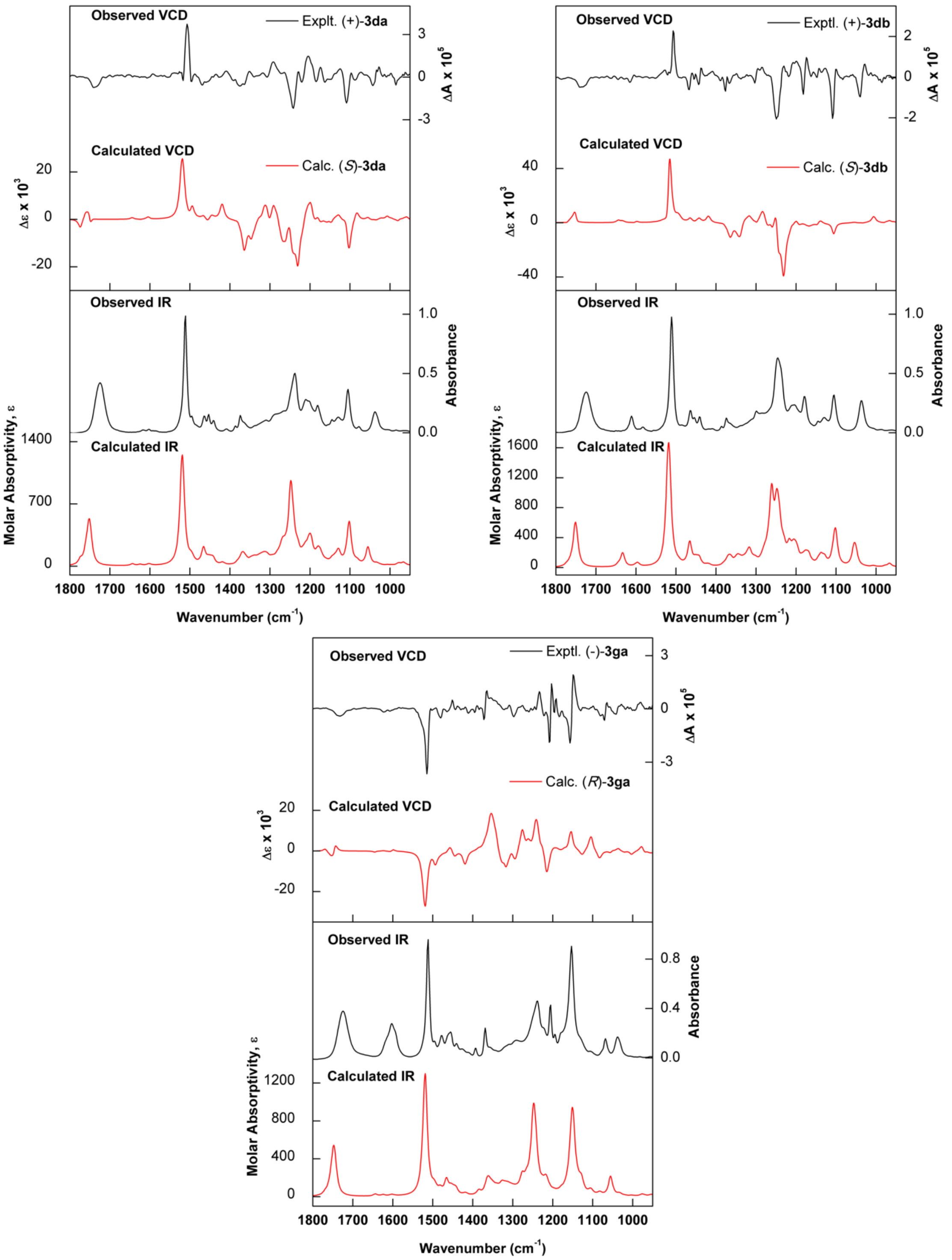

Figure S1. Comparisons of experimental (black traces) and calculated (red traces) IR and VCD spectra for **3da**, **3db** and **3ga**.

III. PHOTOFLOW SETUP

The continuous-flow experiment was carried out using a commercially available Vapourtec R-series device equipped with a UV-150 photoreactor (**Figure S2**).

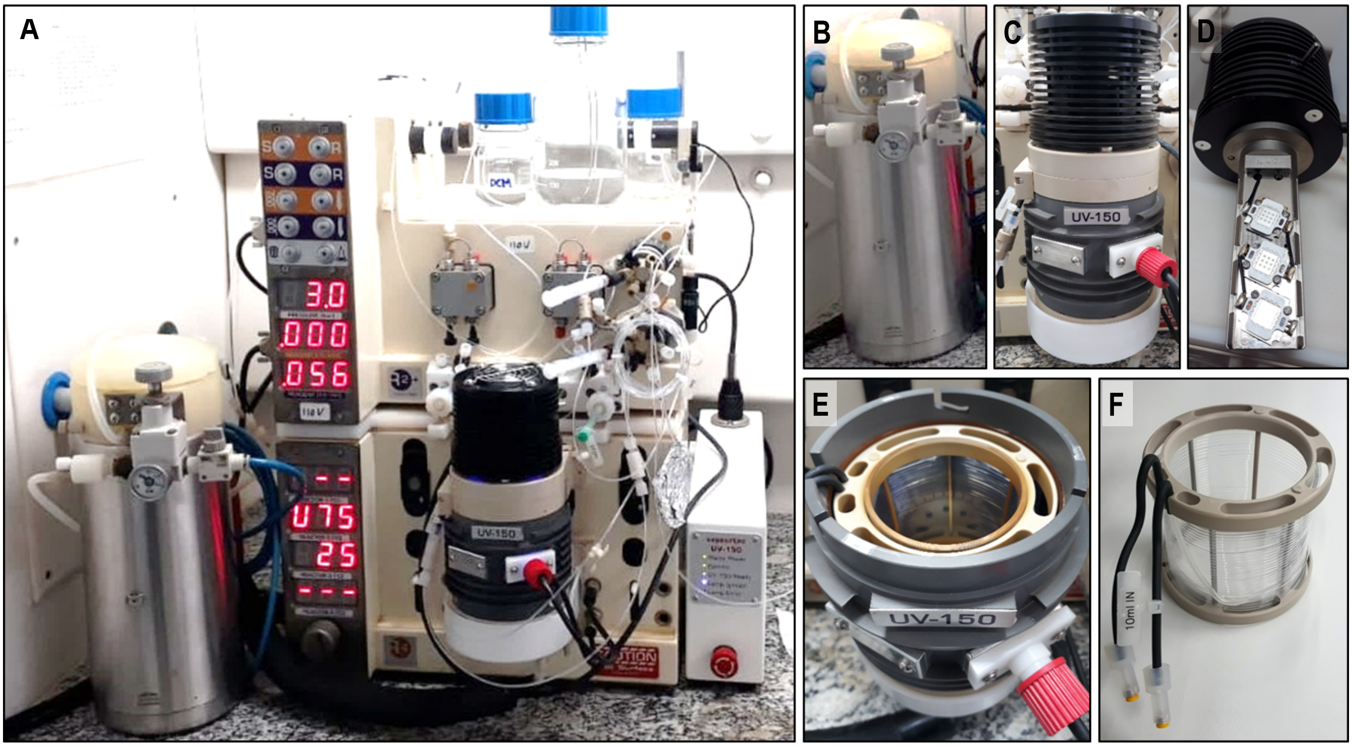

Figure S2. (A) A commercially available Vapourtec R-series flow system equipped with a UV-150 photoreactor in operation. (B) Cooling module. (C) UV-150 photoreactor. (D) LED lamp (440 nm, 60 W) separated from the reactor, with three additional LEDs installed on the opposite side of the module. (E) Internal view of the reactor housing after removal of the LED lamp (The LED shown in (D) sits in the centre and irradiates the tubing in 360°). (F) A 10 mL fluoropolymer reactor coil (inner diameter: 1.3 mm, wall: 0.15 mm).

In the system shown (**Fig. S2.A**), the reactor temperature is controlled by a thermocouple, identified by the red adaptor on the side of the reactor, which regulates Vapourtec's cooling system. This system consists of a Dewar (**Fig. S2.B**) containing dry ice and an insulated gas supply tube. Cooling is achieved by passing dry nitrogen or dry compressed air, at low pressure, through a bed of dry ice. The resulting mixture of nitrogen (or air) and carbon dioxide is chilled and intermittently fed into the cooled reactor assembly (**Fig. S2.C**) via a pinch valve. Inside the assembly, the chilled gas mixture circulates around the reactor (**Fig. S2.F**), ensuring efficient heat transfer between the gas and the reactor, with an operational temperature range of –20 °C to 40 °C.

The reaction at −65 to −60 °C was carried out in a 1 mL PFA coil reactor (i.d. 0.79 mm), immersed in an acetone/dry ice bath, and irradiated with blue light (Kessil, λ = 440 nm) positioned 15 cm from the reactor. The reaction mixture was pumped at a flow rate of 0.033 mL·min^{-1}, using a 100 psi back-pressure regulator (BPR; ca. 6.9 bar) at the reactor outlet (**Figure S3**).

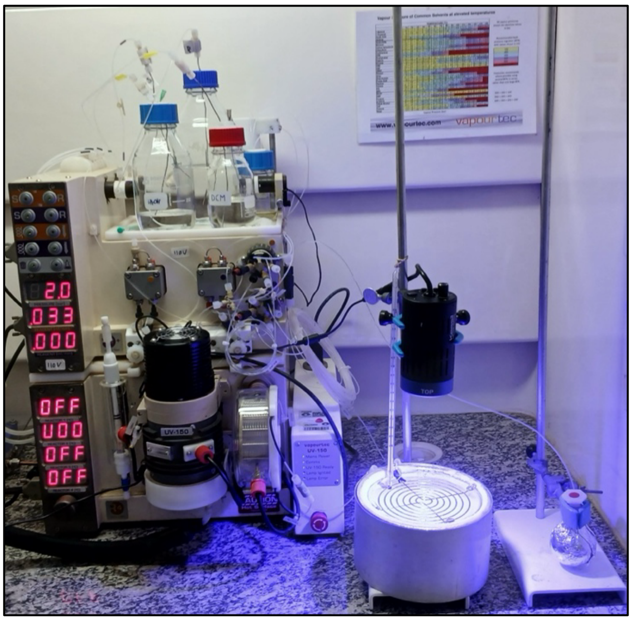

Figure S3. Setup for reaction performed at – 65-60 °C.

IV. BATCH REACTION SETUP

Batch reactions were carried out in a 10 mL Schlenk tube equipped with a stir bar. The tube, containing the reaction mixture, was sealed under a nitrogen atmosphere and positioned 5 cm away from the specified light source (Kessil lamp). The reaction mixture was stirred at room temperature for a determined period.

V. SYNTHESIS OF SUBSTRATES

The substrates α-imino esters (**1**),[3] dihydropyridines (**DHP**, **2**),[4,5] and the **RhS** catalyst were synthesized according to previously reported procedures.[6]

VI.I Procedure A: Synthesis of substrates α-imino ester 1

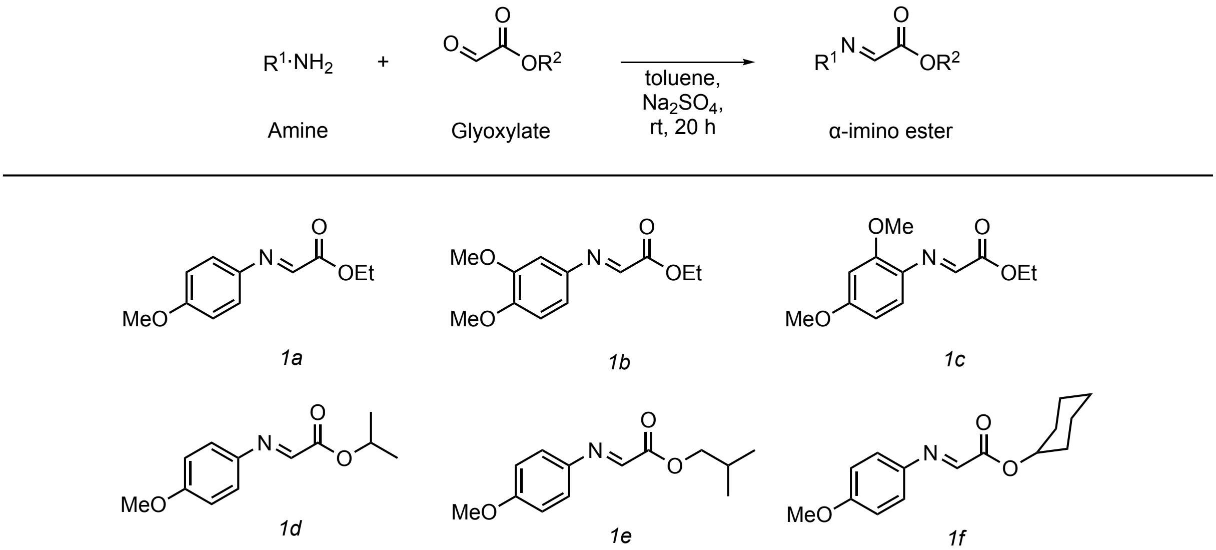

Scheme S1. General procedure for the synthesis of substrates 1.

To a solution of the corresponding amine (1.0 equiv.) in anhydrous toluene (1 mL/mmol of amine), the corresponding glyoxylate (1.5 equiv.) and sodium sulfate (~3 g) were added. The reaction mixture was stirred at room temperature for 20 hours. After this period, the mixture was concentrated using a rotary evaporator under reduced pressure at 40 °C. The crude product was then purified by flash column chromatography on a Biotage® Isolera One system (25 g cartridge) using isocratic elution with dichloromethane (CH_2Cl_2), affording the desired product α-imino ester.

VI.II Procedure B: Synthesis of substrate α-imino ester 1g

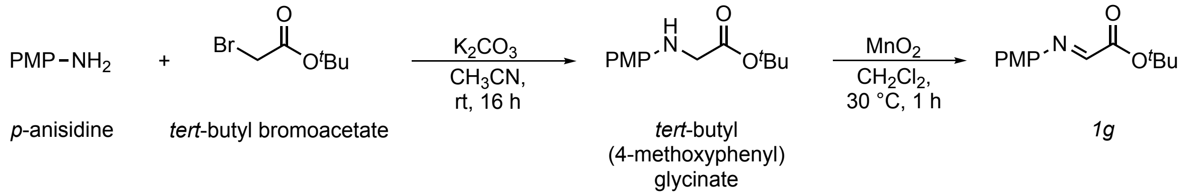

Scheme S2. General procedure for the synthesis of substrate 1g.

To a solution of *p*-anisidine (1 equiv.) in CH_3CN (100 mL) were added K_2CO_3 (1.1 equiv.) and *tert*-butyl bromoacetate (1 equiv.) at room temperature. The mixture was stirred at this temperature for 16 h. The insoluble material was removed by filtration, and the filtrate was concentrated under reduced pressure at 40 °C. The crude residue was purified by flash column chromatography on a Biotage® Isolera One system (25 g cartridge) using a gradient

elution system (hexane:EtOAc, 4:1–2:1) to afford *tert*-butyl (4-methoxyphenyl)glycinate in 89% yield.

In the next step, a solution of tert-butyl (4-methoxyphenyl)glycinate (2.00 mmol) in CH_2Cl_2 (0.01 M) was treated with MnO_2 (20 equiv.) at room temperature, and the mixture was stirred for 1 h. The reaction mixture was then filtered through Celite, and the filtrate was concentrated *in vacuo*. The crude product was purified by flash column chromatography on a Biotage® Isolera One system (25 g cartridge) using isocratic elution with CH_2Cl_2 to give the corresponding α-imino ester **1g** (183 mg, 39% yield) as a red oil. This procedure follows a reported protocol.[7]

VI.III Procedure C: Synthesis of substrate α-imino ester 1h

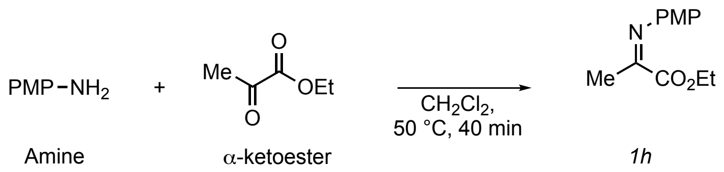

Scheme S3. General procedure for the synthesis of substrate 1h.

To a solution of *p*-anisidine (1.0 equiv.) in CH_2Cl_2 (0.5 M) was added α-ketoester (1.0 equiv.) at room temperature. The resulting reaction mixture was stirred at 50 °C for 40 min. After this period, the mixture was concentrated using a rotary evaporator under reduced pressure at 40 °C. The crude product was purified by flash column chromatography on a Biotage® Isolera One system (25 g cartridge) using isocratic elution system (hexane:ethyl acetate, 90:10) to give the corresponding α-imino ester **1h** (50% yield) as a yellow oil.[8]

VI.IV Procedure D: Synthesis of substrate α-imino ester 1i

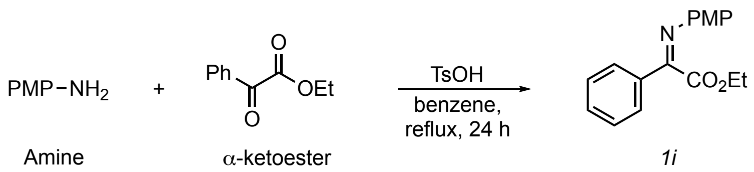

Scheme S4. General procedure for the synthesis of substrate 1i.

In a one-neck round-bottom flask equipped with a magnetic stir bar, a condenser, and a Dean–Stark apparatus were added *p*-anisidine (1.05 equiv.), benzene (0.3 M), α-ketoester (1.0 equiv.), and *p*-toluenesulfonic acid (5 mol%). The reaction mixture was stirred under reflux for 24 h. After this period, the mixture was cooled to room temperature and concentrated using a rotary evaporator under reduced pressure at 40 °C. The crude product was purified by flash

column chromatography on a Biotage® Isolera One system (25 g cartridge) using an isocratic elution system (hexanes/ethyl acetate, 90:10) to afford the corresponding α-imino ester **1i** (62% yield) as a yellow oil.[9]

VI.V Procedure E: Synthesis of DHP 2

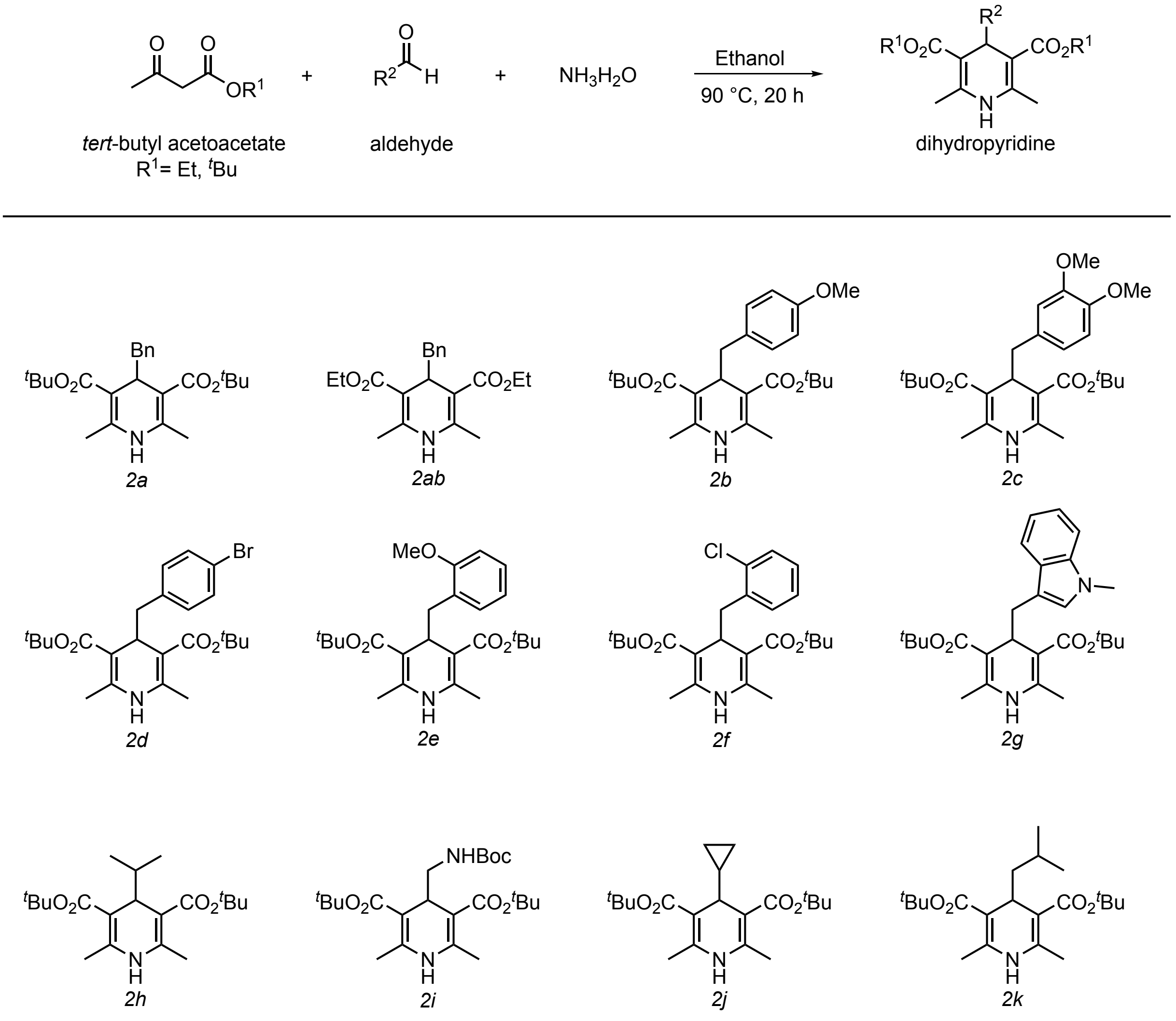

Scheme S5. General procedure for the synthesis of DHP 2.

In an appropriately sized round-bottom flask equipped with a magnetic stirring bar and an air-cooled condenser, ethyl or *tert*-butyl acetoacetate (2.0 equiv.), the corresponding aldehyde (1.0 equiv.), ethanol (2 mL/mmol of aldehyde), and aqueous ammonia solution (10 equiv.) were added in this order. The mixture was stirred at 90 °C for 20 hours. After this period, the reaction mixture was cooled to room temperature, and the solvent was removed using a rotary evaporator at 40 °C under reduced pressure. Water (10 mL) was added to the concentrated residue, and the aqueous layer was extracted with CH_2Cl_2 (3×). The combined organic layers were dried over Na_2SO_4, filtered, and concentrated under reduced pressure. The crude product was purified by automated flash chromatography (50 g cartridge, using an appropriate

hexanes:ethyl acetate gradient elution system) to yield the desired compound. This method, with some modifications, was based on a previously reported protocols.[4,5]

VI.VI General procedure F: Preparation of compound 3ga

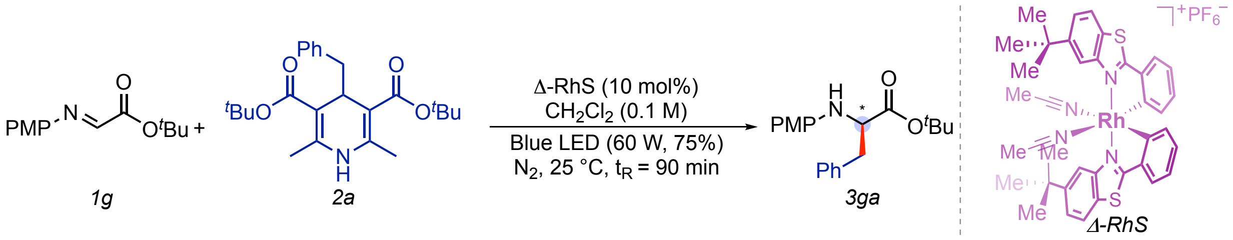

Scheme S6. General procedure for the synthesis of product 3ga.

In a 10 mL Schlenk tube, α-imino ester **1g** (1 equiv.), dihydropyridine **2a** (1.1 equiv.), chiral catalyst **Δ-RhS** (10 mol%), and anhydrous CH_2Cl_2 (0.1 M with respect to **1g**) were combined. The mixture was degassed by three freeze–pump–thaw cycles and then maintained under a nitrogen atmosphere. The solution was transferred to a PFA injection loop and pumped through a 10 mL PFA coil (i.d. 1.3 mm) housed in a UV-150 reactor at a flow rate of 0.111 mL·min^{-1} (residence time (t_R) τ, 90 min) under irradiation with a blue LED (440 nm, 60 W, 75% power) at 25 °C, using a 100 psi back-pressure regulator (BPR; ca. 6.9 bar) at the reactor outlet. After completion, the solvent was removed under reduced pressure (40 °C). The crude product was purified by automated flash chromatography (25 g cartridge, using a gradient elution system hexanes:ethyl acetate, 98:02–95:05) to provide the title compound as a light yellow liquid (75% yield, er 93:07).

VII. REACTION OPTIMIZATION TABLES AND CONTROL EXPERIMENTS

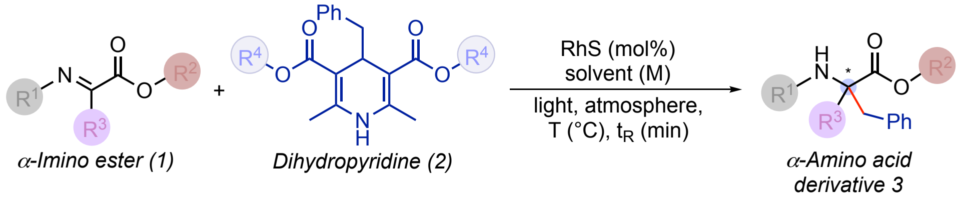

Scheme S7. Overview of the reaction optimization.

The reaction optimization was performed under continuous flow conditions using a commercially available Vapourtec R-series device equipped with a UV-150 photoreactor, unless otherwise indicated. Variations in the equipment's operational parameters, reaction conditions, and substrates were explored, as illustrated in the general **Scheme S7** above and presented in the tables below.

In the preliminary reactions, a screening of dihydropyridines was performed.

Table S1. Preliminary reaction and screening of dihydropyridines.

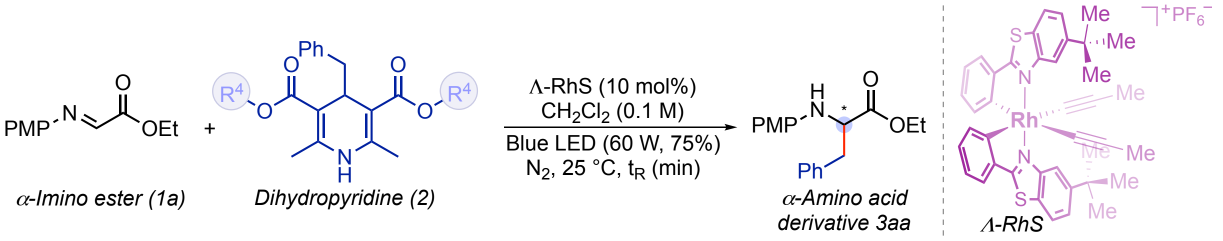

Entry[a]	2 (R)	Residence time (t_R, min)	Yield 3aa (%)[b]	er[d]
1	2a (^{t}Bu)	30	80	22:78
2	2b (Et)	180	26[c]	23:77

All reactions were performed in a commercially available Vapourtec R-series device equipped with a UV-150 photoreactor (Item II. PhotoFlow setup).
[a] Conditions: **1a** (0.05 mmol, 1.0 equiv., 0.1 M in anhydrous CH_2Cl_2), **2** (1.1 equiv.), chiral rhodium catalyst (**Λ-RhS**, 10 mol%), visible light (440 nm, 60 W, 75% power), under N_2 atmosphere, 25 °C, flow rate 0.333 mL·min^{-1} (entry 1: t_R (τ), 30 min) or 0.056 mL·min^{-1} (entry 2: t_R (τ), 180 min).
[b] Isolated yield based on **1a**.
[c] Yield was determined via ^{1}H-NMR by using 1,3,5-Trimethoxybenzene as internal standard.
[d] er: Enantiomeric ratio – Determined by chiral HPLC analysis.

Table S2. Evaluation of residence time and RhS catalyst loading for the reaction studied.

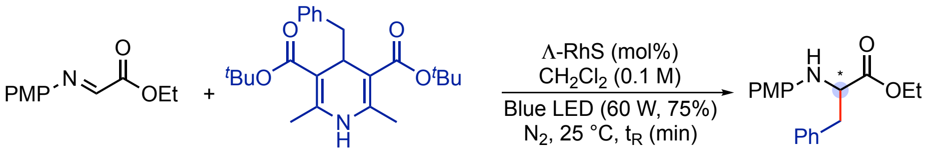

Entry	Residence time (t_R, min)	Λ-RhS (mol%)	Yield 3aa (%)[a]	er[b]
1	180	10	60	21:79
2	60	10	80	22:78
3	15	10	59	22:78
4	30	15	78	22:78
5	30	5	81	25:75

All reactions were performed in a commercially available Vapourtec R-series device equipped with a UV-150 photoreactor (Item II. PhotoFlow setup).
Conditions: **1a** (0.05 mmol, 1.0 equiv., 0.1 M in CH_2Cl_2), **2a** (1.1 equiv.), chiral rhodium catalyst (**Λ-RhS**, 5 to 15 mol%), visible light (440 nm, 60 W, 75% power), under N_2 atmosphere, 25 °C, flow rate (0.667 to 0.056 mL·min^{-1}), residence time (t_R (τ), 15 to 180 min).
[a] Isolated yield based on **1a**.
[b] er: Enantiomeric ratio – Determined by chiral HPLC analysis.

Table S3. Evaluation of the influence of LED power and reaction temperature on the studied reaction.

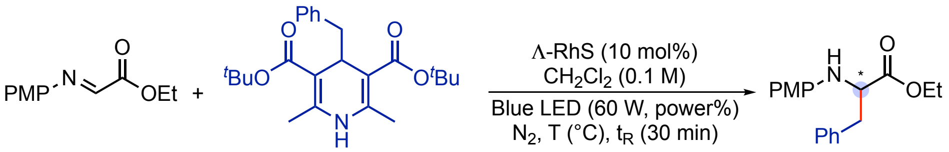

1a 2a 3aa

Entry	LED power (%)	T (°C)	Yield 3a (%)[a]	er[b]
1	50	25	79	22:78
2	75	40	77	25:75
3	75	10	75	25:75

All reactions were performed in a commercially available Vapourtec R-series device equipped with a UV-150 photoreactor (Item II. PhotoFlow setup).
Conditions: **1a** (0.05 mmol, 1.0 equiv., 0.1 M in CH_2Cl_2), **2a** (1.1 equiv.), chiral rhodium catalyst (**Λ-RhS**, 10 mol%), visible light (440 nm, 60 W, power%), under N_2 atmosphere, temperature (10 to 40 °C), flow rate (0.333 mL·min^{-1}), residence time (t_R (τ), 30 min).
[a] Isolated yield based on **1a**.
[b] er: Enantiomeric ratio – Determined by chiral HPLC analysis.

Table S4. Evaluation of substrate α-imino ester.

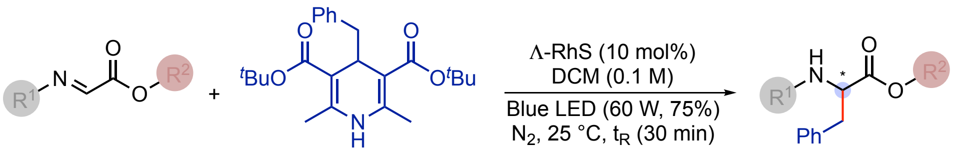

1 2a 3

Entry	α-Imino ester	(R^1)	(R^2)	Yield 3 (%)[a]	er[b]
1	(1b)	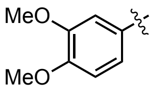	Et	61 (3ba)	29:71
2	(1c)	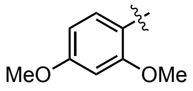	Et	45 (3ca)	47:53
3	(1d)	PMP	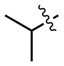	75 (3da)	15:85
4	(1e)	PMP	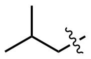	64 (3ea)	15:85
5	(1f)	PMP	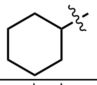	34 (3fa)	20:80

All reactions were performed in a commercially available Vapourtec R-series device equipped with a UV-150 photoreactor (Item II. PhotoFlow setup).
Conditions: **1** (0.05 mmol, 1.0 equiv., 0.1 M in CH_2Cl_2), **2a** (1.1 equiv.), chiral rhodium catalyst (**Λ-RhS**, 10 mol%), visible light (440 nm, 60 W, 75% power), under N_2 atmosphere, temperature (25 °C), flow rate (0.333 mL·min^{-1}), residence time (t_R (τ), 30 min).
[a] Isolated yield based on **1**.
[b] er: Enantiomeric ratio – Determined by chiral HPLC analysis.

Table S5. Evaluation of the influence of LED power and reaction temperature on the studied reaction.

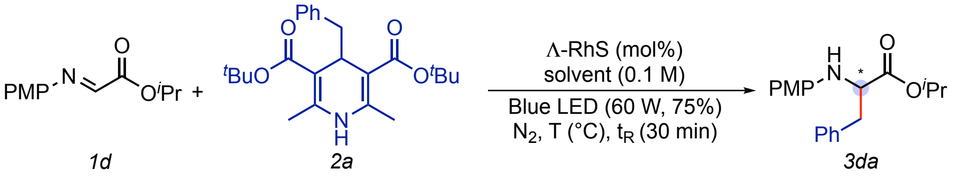

Entry	RhS (mol%)	T (°C)	Solvent	Yield 3da (%)[a]	er[b]
1[c]	10	25	CH_2Cl_2	75	15:85
2	5	25	CH_2Cl_2	70	20:80
3	2.5	25	CH_2Cl_2	69	23:77
4	20	–20	CH_2Cl_2	73	88:12[d]
5	15	–20	CH_2Cl_2	74	88:12[d]
6	10	–20	CH_2Cl_2	74	88:12[d]
7	5	–20	CH_2Cl_2	71	87:13[d]
8	2.5	–20	CH_2Cl_2	69	18:82
9	5	–60	CH_2Cl_2	13	24:76
10[e]	5	–20	CH_2Cl_2	60	23:77
11	5	–20	Toluene	68	81:19[d]
12	5	–20	PhCl	62	17:83
13	5	–20	$PhCF_3$	61	16:84
14	5	–20	DCE	36	69:31[d]
15	5	–20	MeCN	0	-

All reactions were performed in a commercially available Vapourtec R-series device equipped with a UV-150 photoreactor (Item II. PhotoFlow setup).
Conditions: **1d** (1.0 equiv., 0.1 M in CH_2Cl_2), **2a** (1.1 equiv.), chiral rhodium catalyst (**Λ-RhS** or **Δ-RhS**, 1.25 to 20 mol%), visible light (440 nm, 60 W, 75% power), under N_2 atmosphere, temperature (25 °C or –20 °C), flow rate (0.333 mL·min^{-1}), residence time (t_R (τ), 30 min).
[a] Isolated yield based on **1d**.
[b] er: Enantiomeric ratio – Determined by chiral HPLC analysis.
[c] Result from Table S4, entry 3.
[d] The **Δ-RhS** catalyst was used.
[e] Was used **1d** (1.5 equiv.), **2a** (1.0 equiv., 0.1 M in CH_2Cl_2).

Table 6. Control reactions.

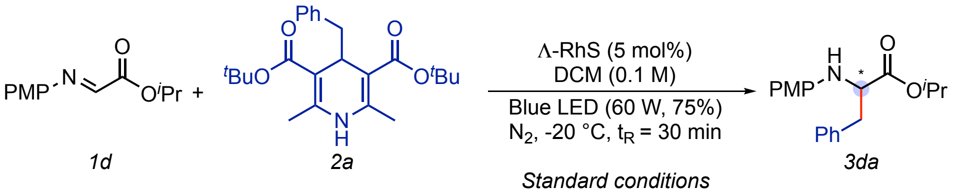

Entry	Deviation from the standard conditions	Yield 3da (%)[a]	er[b]
1	no light	-	-
2	LED 380 nm instead of LED 440 nm	34	84:16
3	no catalyst	4[c]	50:50
4	no RhS catalyst at 25 °C	16[c]	50:50
5	under air[d]	65	84:16
6	addition of 3 equiv of TEMPO	2[c]	-

All reactions were performed in a commercially available Vapourtec R-series device equipped with a UV-150 photoreactor (Item II. PhotoFlow setup).
Conditions: **1d** (1.0 equiv., 0.1 M in CH_2Cl_2), **2a** (1.1 equiv.), chiral rhodium catalyst (**Λ-RhS**, 5 mol%), visible light (440 nm, 60 W, 75% power), under N_2 atmosphere, temperature (–20 °C), flow rate (0.333 mL·min^{-1}), residence time (t_R (τ), 30 min).
[a] Isolated yield based on **1d**.
[b] er: Enantiomeric ratio – Determined by chiral HPLC analysis.
[c] The yield was determined by ^{1}H-NMR using 1,3,5-Trimethoxybenzene as internal standard.
[d] Non-degassed solvent.

Initially, we proposed the use of glyoxylates (commercially available and also readily accessible from tartrate esters) as precursors for the preparation of α-imino esters. However, upon observing low enantiomeric ratios under the optimized conditions employing α-imino ester **1d**, we reconsidered our strategy. The results (**Tables S1** to **S5**) suggested that structural variations in the ester group were the factor exerting the greatest influence on enantioinduction. Consequently, we opted for the synthesis of the α-imino ester derived from the *tert*-butyl ester. Product **1g** was obtained from the reaction between *p*-anisidine and *tert*-butyl bromoacetate, followed by oxidation with MnO_2 to afford the desired α-imino ester (**Procedure B, Scheme S2**).

The reaction optimization using substrate **1g** is presented in the tables below.

Table S7. Optimization of the reaction employing α-imino ester 1g: Evaluation of catalyst loading, temperature, and residence time.

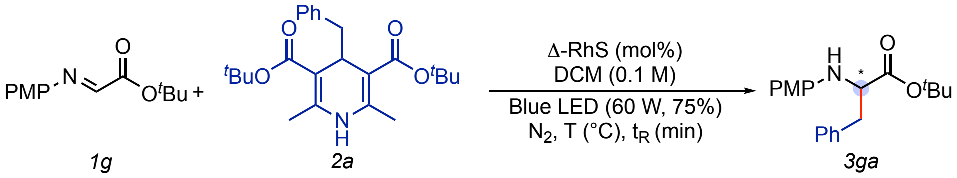

Entry	RhS (mol%)	T (°C)	t_R (min)	Yield 3ga (%)[a]	er[b]
1	15	–20	30	28	93:07
2	10	–20	30	21	93:07
3	5	–20	30	20	88:12
4	10	25	30	35[c]	93:07
5	5	25	30	16	13:87[d]
6	10	25	60	44	93:07
7	10	25	90	75(79)[c]	93:07
8	10	25	90	74	14:86[e, d]

All reactions were performed in a commercially available Vapourtec R-series device equipped with a UV-150 photoreactor (Item II. PhotoFlow setup).
Conditions: **1g** (1.0 equiv., 0.1 M in CH_2Cl_2), **2a** (1.1 equiv.), chiral rhodium catalyst (**Λ-RhS** or **Δ-RhS**, 5 to 15 mol%), visible light (440 nm, 60 W, 75% power), under N_2 atmosphere, temperature (25 °C or –20 °C), flow rate (0.111 to 333 mL·min^{-1}), residence time (t_R (τ), 30 min to 90 min).
[a] Isolated yield based on **1g**.
[b] er: Enantiomeric ratio – Determined by chiral HPLC analysis.
[c] Yield determined by ^{1}H NMR using 1,3,5-Trimethoxybenzene as internal standard.
[d] Reaction with **Λ-RhS** catalyst
[e] Conditions: **1g** (1.5 equiv.), **2a** (1.0 equiv., 0.1 M in CH_2Cl_2).

Table S8. Control experiments.

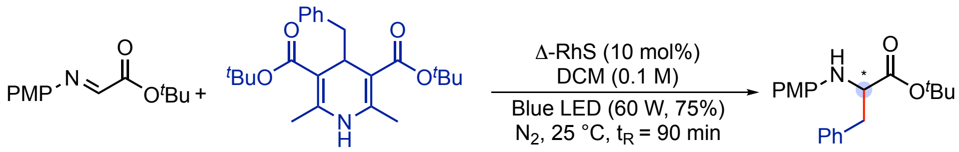

1g *2a* *Standard conditions* *3ga*

Entry	Deviation from the standard conditions	Yield 3ga (%)[a]	er[b]
1	no light	0	-
2	no catalyst	4[c]	50:50
3	under air[d]	56	92:8
4	addition of 3 equiv of TEMPO	<1[c]	-
5	batch reaction, 2 h	60	14:86

All reactions were performed in a commercially available Vapourtec R-series device equipped with a UV-150 photoreactor (Item II. PhotoFlow setup).
Standard conditions: **1g** (1.0 equiv., 0.1 M in CH_2Cl_2), **2a** (1.1 equiv.), chiral rhodium catalyst (**Λ-RhS**, 10 mol%), visible light (440 nm, 60 W, 75% power), under N_2 atmosphere, temperature (25 °C), flow rate (0.111 mL·min^{-1}), residence time (t_R (τ), 90 min).
[a] Isolated yield based on **1g**.
[b] er: Enantiomeric ratio – Determined by chiral HPLC analysis.
[c] The yield was determined by ^{1}H-NMR using 1,3,5-Trimethoxybenzene as internal standard.
[d] Non-degassed solvent.

VIII. ROBUSTNESS EXPERIMENTS

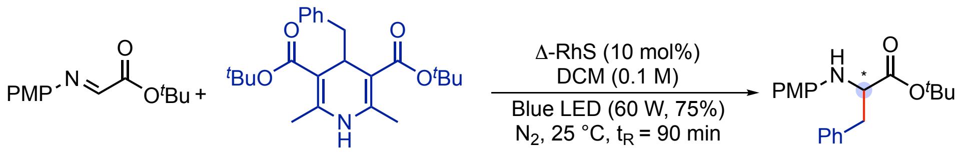

1g *2a* *Standard conditions* *3ga*

To assess the reproducibility and robustness of the optimized conditions, the reaction between α-imino ester **1g** and dihydropyridine **2a** was performed in triplicate under standard conditions on a 0.05 mmol scale (General Procedure F). The desired product **3ga** was obtained in 73%, 75%, and 76% isolated yield, corresponding to an average yield of 74.7% with a standard deviation of 1.53%. The enantiomeric ratios were determined to be 92.8:7.2 (±0.3). The low variability observed across independent experiments highlights the robustness and reliability of the developed flow protocol in terms of both yield and enantioselectivity.

IX. CHARACTERIZATION DATA

Compound 2g

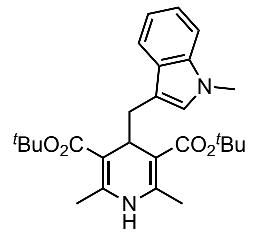

di-*tert*-butyl 2,6-dimethyl-4-((1-methyl-1*H*-indol-3-yl)methyl)-1,4-dihydropyridine-3,5-dicarboxylate
Chemical Formula: $C_{27}H_{36}N_2O_4$
Exact Mass: 452,2675
Molecular Weight: 452,5950

Following General Procedure (E), in a scale of 2.25 mmol afforded 460 mg (45% yield) of the title compound after purification by automated flash chromatography (50 g cartridge, using a gradient elution system hexanes:ethyl acetate, 95:5–80:20).

Physical properties: light yellow solid.

^{1}H NMR (600 MHz, $CDCl_3$) δ (ppm): 7.63 (dt, *J* = 7.9, 1.0 Hz, 1H), 7.21 (dt, *J* = 8.2, 0.9 Hz, 1H), 7.16 – 7.13 (m, 1H), 7.09 – 7.03 (m, 1H), 6.64 (s, 1H), 5.29 (s, 1H), 4.16 (t, *J* = 6.1 Hz, 1H), 3.69 (s, 3H), 2.67 (d, *J* = 6.2 Hz, 2H), 2.15 (s, 6H), 1.31 (s, 18H).

^{13}C{^{1}H} NMR (151 MHz, $CDCl_3$) δ (ppm): 167.6, 144.0, 137.0, 129.2, 127.6, 121.1, 119.7, 118.4, 112.2, 108.8, 104.5, 79.2, 35.2, 32.6, 31.1, 28.2, 19.4.

HRMS (ESI-TOF) m/z calcd. for $C_{27}H_{37}N_2O_4^+$ (M+H) 453.2748, found 453.2741.

Compound 2f

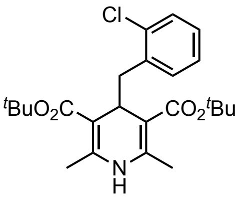

di-*tert*-butyl 4-(2-chlorobenzyl)-2,6-dimethyl-1,4-dihydropyridine-3,5-dicarboxylate
Chemical Formula: $C_{24}H_{32}ClNO_4$
Exact Mass: 433,2020
Molecular Weight: 433,9730

Following General Procedure (E), in a scale of 4.37 mmol afforded 389 mg (21% yield) of the title compound after purification by automated flash chromatography (50 g cartridge, using a gradient elution system hexanes:ethyl acetate, 95:5–80:20).

Physical properties: white solid.

^{1}H NMR (300 MHz, $CDCl_3$) δ (ppm): 7.30 – 7.23 (m, 1H), 7.12 – 7.05 (m, 2H), 7.04 – 6.98 (m, 1H), 5.48 (s, 1H), 4.29 (t, *J* = 6.4 Hz, 1H), 2.70 (d, *J* = 6.4 Hz, 2H), 2.22 (s, 6H), 1.40 (s, 18H).

^{13}C{^{1}H} NMR (151 MHz, $CDCl_3$) δ (ppm): 167.1, 144.8, 137.2, 135.1, 132.7, 129.1, 127.3, 125.9, 103.4, 79.4, 38.3, 34.5, 28.4, 19.5.

HRMS (ESI-TOF) m/z calcd. for $C_{24}H_{33}ClNO_4^+$ (M+H) 434.2093, found 434.2088.

Compound 3aa[10]

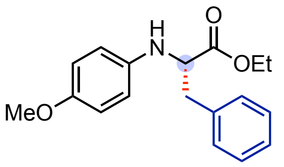

ethyl (4-methoxyphenyl)-*L*-phenylalaninate
Chemical Formula: $C_{18}H_{21}NO_3$
Exact Mass: 299,1521
Molecular Weight: 299,3700

Following General Procedure (F) with **1b** (1.0 equiv., 0.05 mmol), **2a** (1.1 equiv., 0.055 mmol), and Λ-RhS (5 mol%), flow rate of 0.333 mL·min^{-1} (τ = 30 min), blue LED (440 nm, 60 W, 75% power), at 25 °C, afforded 12 mg (80% yield) of the title compound after purification by automated flash chromatography (25 g cartridge, using a gradient elution system hexanes:ethyl acetate, 95:5–90:10).

Physical properties: light yellow liquid.

TLC, R_f : 0.33 (hexanes:ethyl acetate, 90:10; UV active).

^{1}H NMR (300 MHz, $CDCl_3$) δ (ppm): 7.35 – 7.13 (m, 5H), 6.81 – 6.70 (m, 2H), 6.63 – 6.53 (m, 2H), 4.25 (s, 1H), 4.10 (q, *J* = 7.1 Hz, 2H), 3.89 (s, 1H), 3.73 (s, 3H), 3.10 (d, *J* = 6.4 Hz, 2H), 1.15 (t, *J* = 7.1 Hz, 3H).

^{13}C{^{1}H} NMR (151 MHz, $CDCl_3$) δ (ppm): 173.6, 152.9, 140.7, 136.7, 129.5, 128.6, 127.1, 115.4, 115.0, 61.1, 59.2, 55.8, 39.1, 14.3.

IR (film), $\bar{\nu}_{max}$ (cm^{-1}): 2984, 2917, 2849, 1723, 1514, 1455, 1370, 1240, 1154, 1119, 1032, 846, 822, 740, 701.

Enantiomeric ratio, er: 22:78 (HPLC: Daicel Chiralpak IA column, 4.6 i.d. x 250 mm, λ 260 nm, hexane/^{i}PrOH = 80:20, flow rate 1.0 mL·min^{-1}, 25 °C, tr (major) = 9.7 min, tr (minor) = 6.7 min).

Specific rotation, $[\alpha]_D^{24}$: = +9.3 (*c* 0.05, $CHCl_3$).

Compound 3ba

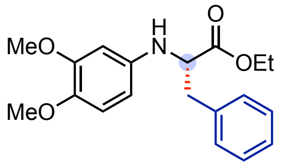

ethyl (3,4-dimethoxyphenyl)-*L*-phenylalaninate
Chemical Formula: $C_{19}H_{23}NO_4$
Exact Mass: 329,1627
Molecular Weight: 329,3960

Following General Procedure (F) with **1b** (1.0 equiv., 0.05 mmol), **2a** (1.1 equiv., 0.055 mmol), and Λ-RhS (10 mol%), flow rate of 0.333 mL·min^{-1} (τ = 30 min), blue LED (440 nm, 60 W, 75% power), at 25 °C, afforded 10.0 mg (61% yield) of the title compound after purification by automated flash chromatography (25 g cartridge, using a gradient elution system hexanes:ethyl acetate, 95:05–70:30).

Physical properties: light yellow liquid.

TLC, R_f: 0.28 (hexanes:ethyl acetate, 85:15; UV active).

^{1}H NMR (300 MHz, $CDCl_3$) δ (ppm): 7.36 – 7.14 (m, 5H), 6.71 (d, J = 8.6 Hz, 1H), 6.22 (d, J = 2.6 Hz, 1H), 6.14 (dd, J = 8.5, 2.7 Hz, 1H), 4.26 (t, J = 6.4 Hz, 1H), 4.11 (qd, J = 7.1, 1.0 Hz, 2H), 3.94 (s, 1H), 3.80 (s, 3H), 3.79 (s, 3H), 3.11 (d, J = 6.3 Hz, 2H), 1.16 (t, J = 7.2 Hz, 3H).

$^{13}C\{^1H\}$ NMR (75 MHz, $CDCl_3$) δ (ppm): 173.6, 150.1, 142.4, 141.4, 136.7, 129.5, 128.7, 127.1, 113.1, 104.8, 100.1, 61.2, 59.1, 56.7, 55.9, 39.2, 14.3.

IR (film), $\bar{\nu}_{max}$ (cm^{-1}): 3370, 2929, 2829, 1733, 1619, 1517, 1454, 1235, 1209, 1169, 1139, 1026, 956, 833, 764, 701, 633.

HRMS (ESI-TOF) m/z calcd. for $C_{19}H_{24}NO_4^+$ (M+H) 330.1700, found 330.1696.

Enantiomeric ratio, er: 29:71 (HPLC: Daicel Chiralpak IB column, 4.6 i.d. x 250 mm, λ 300 nm, hexane/^{i}PrOH = 90:10, flow rate 1.0 mL·min^{-1}, 25 °C, tr (major) = 15 min, tr (minor) = 14 min).

Specific rotation, $[\alpha]_D^{24}$: −4.5 (0.01, $CHCl_3$).

Compound 3ca

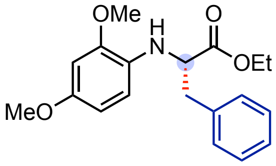

ethyl (2,4-dimethoxyphenyl)-*L*-phenylalaninate
Chemical Formula: $C_{19}H_{23}NO_4$
Exact Mass: 329,1627
Molecular Weight: 329,3960

Following General Procedure (F) with **1c** (1.0 equiv., 0.05 mmol), **2a** (1.1 equiv., 0.055 mmol), and Λ-RhS (10 mol%), flow rate of 0.333 mL·min^{-1} (τ = 30 min), blue LED (440 nm, 60 W, 75% power), at 25 °C, afforded 7.4 mg (45% yield) of the title compound after purification by automated flash chromatography (25 g cartridge, using a gradient elution system hexanes:ethyl acetate, 95:5–90:10).

Physical properties: light yellow liquid.

TLC, R_f: 0.52 (hexanes:ethyl acetate, 85:15; UV active).

^{1}H NMR (300 MHz, $CDCl_3$) δ (ppm): 7.34 – 7.14 (m, 5H), 6.48 (d, *J* = 8.6 Hz, 1H), 6.44 (d, *J* = 2.6 Hz, 1H), 6.35 (dd, *J* = 8.5, 2.7 Hz, 1H), 4.44 (d, *J* = 8.4 Hz, 1H), 4.32 – 4.18 (m, 1H), 4.07 (q, *J* = 7.2 Hz, 2H), 3.80 (s, 3H), 3.74 (s, 3H), 3.12 (dd, *J* = 6.7, 2.3 Hz, 2H), 1.12 (t, *J* = 7.1 Hz, 3H).

^{13}C{^{1}H} NMR (75 MHz, $CDCl_3$) δ (ppm): 173.7, 152.8, 148.6, 137.0, 130.8, 129.4, 128.6, 127.0, 111.5, 103.9, 99.6, 61.0, 58.9, 55.9, 55.7, 39.2, 14.2.

IR (film), $\bar{\nu}_{max}$ (cm^{-1}): 3381, 2980, 2930, 2840, 1736, 1601, 1519, 1456, 1290, 1259, 1205, 1156, 1033, 930, 835, 790, 742, 701.

HRMS (ESI-TOF) m/z calcd. for $C_{19}H_{24}NO_4^+$ (M+H) 330.1700, found 330.1695.

Enantiomeric ratio, er: 47:53 [HPLC: Daicel Chiralpak IA chiral analytical column (250 × 4.6 mm i.d.), λ 300 nm, hexane/^{i}PrOH = 80:20, flow rate 1.0 mL·min^{-1}, 25 °C, tr (major) = 14.3 min, tr (minor) = 8.1 min].

Specific rotation, $[\alpha]_D^{24}$: +4.7 (*c* 0.03, $CHCl_3$).

Compound 3da

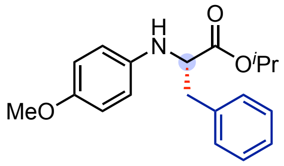

isopropyl (4-methoxyphenyl)-*L*-phenylalaninate
Chemical Formula: $C_{19}H_{23}NO_3$
Exact Mass: 313,1678
Molecular Weight: 313,3970

Following General Procedure (F) with **1d** (1.0 equiv., 0.05 mmol), **2a** (1.1 equiv., 0.055 mmol), and Λ-RhS (5 mol%), flow rate of 0.333 mL·min^{-1} (τ = 30 min), blue LED (440 nm, 60 W, 75% power), at –20 °C, afforded 11.2 mg (71% yield) of the title compound after purification by automated flash chromatography (25 g cartridge, using a gradient elution system hexanes:ethyl acetate, 95:5–90:10).

Physical properties: light yellow liquid.

TLC, R_f: 0.32 (hexanes:ethyl acetate, 90:10; UV active).

^{1}H NMR (500 MHz, $CDCl_3$) δ (ppm): 7.30 – 7.26 (m, 2H), 7.25 – 7.18 (m, 3H), 6.77 – 6.73 (m, 2H), 6.60 – 6.56 (m, 2H), 4.95 (hept, *J* = 6.3 Hz, 1H), 4.22 (t, *J* = 6.5 Hz, 1H), 3.73 (s, 3H), 3.10 – 3.07 (m, 2H), 1.16 (d, *J* = 6.3 Hz, 3H), 1.06 (d, *J* = 6.3 Hz, 3H).

^{13}C{^{1}H} NMR (126 MHz, $CDCl_3$): 173.2, 152.9, 140.8, 136.7, 129.5, 128.6, 127.0, 115.4, 115.0, 68.7, 59.24, 55.9, 39.0, 21.9, 21.7.

IR (film), $\bar{\nu}_{max}$ (cm^{-1}): 2981, 2917, 2849, 1731, 1514, 1455, 1374, 1239, 1200, 1106, 1039, 821, 742, 701.

HRMS (ESI-TOF) m/z calcd. for $C_{19}H_{24}NO_3^+$ ([M+H) 314.1751, found 314.1747.

Enantiomeric ratio, er: 12:88 [HPLC: Daicel Chiralpak IA chiral analytical (250 x 4.6 mm i.d.), λ 310 nm, hexane/^{i}PrOH = 80:20, flow rate 1.0 mL·min^{-1}, 25 °C, tr (major) = 7.1 min, tr (minor) = 5.2 min].

Specific rotation, $[\alpha]_D^{23}$: +12.8 (*c* 0.05, $CHCl_3$).

Compound 3db

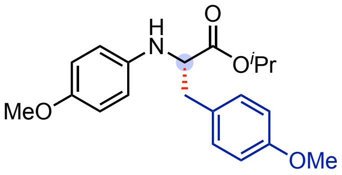

isopropyl (*S*)-3-(4-methoxyphenyl)-2-((4-methoxyphenyl)amino)propanoate
Chemical Formula: $C_{20}H_{25}NO_4$
Exact Mass: 343,1784
Molecular Weight: 343,4230

Following General Procedure (F) with **1d** (1.0 equiv., 0.05 mmol), **2b** (1.1 equiv., 0.055 mmol), and Λ-RhS (5 mol%), flow rate of 0.333 mL·min^{-1} (τ = 30 min), blue LED (440 nm, 60 W, 75% power), at –20 °C, afforded 12.5 mg (73% yield) of the title compound after purification by automated flash chromatography (25 g cartridge, using a gradient elution system hexanes:ethyl acetate, 95:05–85:15).

Physical properties: light yellow liquid.

TLC, R_f: 0.40 (hexanes:ethyl acetate, 85:15; UV active).

^{1}H NMR (500 MHz, $CDCl_3$) δ (ppm): 7.11 (d, *J* = 8.6 Hz, 2H), 6.82 (d, *J* = 8.6 Hz, 2H), 6.77 – 6.71 (m, 2H), 6.60 – 6.55 (m, 2H), 4.96 (hept, *J* = 6.3 Hz, 1H), 4.17 (t, *J* = 6.4 Hz, 1H), 3.78 (s, 3H), 3.73 (s, 3H), 3.03 (d, *J* = 6.4 Hz, 2H), 1.18 (d, *J* = 6.3 Hz, 3H), 1.09 (d, *J* = 6.3 Hz, 3H).

^{13}C{^{1}H} NMR (126 MHz, $CDCl_3$) δ (ppm): 173.2, 158.7, 152.8, 140.8, 130.5, 128.7, 115.4, 115.0, 114.0, 68.7, 59.3, 55.8, 55.4, 38.1, 22.0, 21.8.

IR (film), $\bar{\nu}_{max}$ (cm^{-1}): 2948, 2924, 2854, 1735, 1614, 1515, 1460, 1375, 1300, 1251, 1182, 1109, 1039, 914, 824, 740, 695.

HRMS (ESI-TOF) m/z calcd. for $C_{20}H_{26}NO_4^+$ (M+H) 344.1856, found 344.1854.

Enantiomeric ratio, er: 17:83 [HPLC: Daicel Chiralpak IA chiral analytical column (250 × 4.6 mm i.d.), λ 254 nm, hexane/^{i}PrOH = 80:20, flow rate 1.0 mL·min^{-1}, 25 °C, tr (major) = 8.3 min, tr (minor) = 6.0 min].

Specific rotation, $[\alpha]_D^{22}$: +16.4 (*c* 0.05, $CHCl_3$).

Compound 3dh[11]

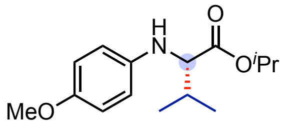

isopropyl (4-methoxyphenyl)-*L*-valinate
Chemical Formula: $C_{15}H_{23}NO_3$
Exact Mass: 265,1678
Molecular Weight: 265,3530

Following General Procedure (F) with **1d** (1.0 equiv., 0.05 mmol), **2h** (1.1 equiv., 0.055 mmol), and Λ-RhS (5 mol%), flow rate of 0.333 mL·min^{-1} (τ = 30 min), blue LED (440 nm, 60 W, 75% power), at –20 °C, afforded 6.0 mg (45% yield) of the title compound after purification by automated flash chromatography (25 g cartridge, using a gradient elution system hexanes:ethyl acetate, 95:05–90:10).

Physical properties: light yellow liquid.

TLC, R_f: 0.58 (hexanes:ethyl acetate, 90:10; UV active).

^{1}H NMR (500 MHz, $CDCl_3$) δ (ppm): 6.78 – 6.73 (m, 2H), 6.64 – 6.60 (m, 2H), 5.03 (hept, J = 6.3 Hz, 1H), 3.73 (s, 3H), 3.71 (d, J = 5.9 Hz, 1H), 2.13 – 2.03 (m, 1H), 1.20 (dd, J = 11.1, 6.3 Hz, 6H), 1.03 (dd, J = 9.7, 6.8 Hz, 6H).

^{13}C{^{1}H} NMR (126 MHz, $CDCl_3$) δ (ppm): 173.5, 152.8, 141.6, 115.5, 114.9, 68.5, 64.1, 55.9, 31.6, 22.04, 21.97, 19.2, 18.9.

IR (film), $\bar{\nu}_{max}$ (cm^{-1}): 3380, 2966, 2930, 1727, 1513, 1467, 1373, 1236, 1195, 1150, 1107, 1039, 983, 933, 821, 755, 669.

Enantiomeric ratio, er: 44:56 [HPLC: Daicel Chiralpak IA chiral analytical column (250 × 4.6 mm i.d.), λ 254 nm, hexane/^{i}PrOH = 80:20, flow rate 1.0 mL·min^{-1}, 25 °C, tr (major) = 4.7 min, tr (minor) = 4.1 min].

Specific rotation, $[\alpha]_D^{20}$: not determined.

Compound 3di

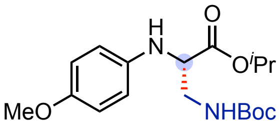

isopropyl (*S*)-3-((*tert*-butoxycarbonyl)amino)-2-((4-methoxyphenyl)amino)propanoate
Chemical Formula: $C_{18}H_{28}N_2O_5$
Exact Mass: 352,1998
Molecular Weight: 352,4310

Following General Procedure (F) with **1d** (1.0 equiv., 0.05 mmol), **2i** (1.1 equiv., 0.055 mmol), and Λ-RhS (5 mol%), flow rate of 0.333 mL·min^{-1} (τ = 30 min), blue LED (440 nm, 60 W, 75% power), at -20 °C, afforded 12.1 mg (69% yield) of the title compound after purification by automated flash chromatography (25 g cartridge, using a gradient elution system hexanes:ethyl acetate, 95:5–70:30).

Physical properties: light yellow liquid.

TLC, R_f: 0.13 (hexanes:ethyl acetate, 90:10; UV active).

^{1}H NMR (500 MHz, $CDCl_3$) δ (ppm): 6.79 – 6.74 (m, 2H), 6.65 – 6.60 (m, 2H), 5.09 – 4.99 (m, 1H), 4.91 (s, 1H), 4.16 (s, 1H), 4.06 (t, *J* = 5.3 Hz, 1H), 3.74 (s, 3H), 3.60 – 3.44 (m, 2H), 1.44 (s, 9H), 1.24 (d, *J* = 3.1 Hz, 3H), 1.23 (d, *J* = 3.1 Hz, 3H).

^{13}C{^{1}H} NMR (126 MHz, $CDCl_3$) δ (ppm): 172.1, 156.1, 153.1, 140.9, 115.5, 115.0, 79.8, 69.5, 58.4, 55.9, 42.7, 28.5, 21.9, 21.8.

IR (film), $\bar{\nu}_{max}$ (cm^{-1}): 3361, 2979, 2929, 2914, 2855, 1719, 1514, 1368, 1242, 1166, 1106, 1036, 8219.

HRMS (ESI-TOF) m/z calcd. for $C_{18}H_{29}N_2O_5^+$ (M+H) 353.2071, found 353.2067.

Enantiomeric ratio, er: 35:65 [HPLC: Daicel Chiralpak IA chiral analytical column (250 × 4.6 mm i.d.), λ 210 nm, hexane/^{i}PrOH = 80:20, flow rate 1.0 mL·min^{-1}, 25 °C, tr (major) = 7.4 min, tr (minor) = 6.1 min].

Specific rotation, $[\alpha]_D^{22}$: +5.8 (*c* 0.03, $CHCl_3$).

Compound 3ea

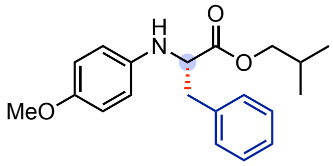

isobutyl (4-methoxyphenyl)-*L*-phenylalaninate
Chemical Formula: $C_{20}H_{25}NO_3$
Exact Mass: 327,1834
Molecular Weight: 327,4240

Following General Procedure (F) with **1e** (1.0 equiv., 0.05 mmol), **2a** (1.1 equiv., 0.055 mmol), and Λ-RhS (10 mol%), flow rate of 0.333 mL·min^{-1} (τ = 30 min), blue LED (440 nm, 60 W, 75% power), at 25 °C, afforded 10.5 mg (64% yield) of the title compound after purification by automated flash chromatography (25 g cartridge, using a gradient elution system hexanes:ethyl acetate, 95:5–90:10).

Physical properties: colorless liquid.

TLC, R_f : 0.45 (hexanes:ethyl acetate, 90:10; UV active).

^{1}H NMR (500 MHz, CD_3CN) δ (ppm): 7.33 (s, 2H), 7.28 – 7.23 (m, 1H), 7.23 – 7.19 (m, 2H), 6.80 – 6.76 (m, 2H), 6.65 – 6.58 (m, 2H), 4.30 (t, *J* = 6.5 Hz, 1H), 3.83 (ddd, *J* = 25.9, 10.5, 6.6 Hz, 2H), 3.76 (s, 3H), 3.13 (d, *J* = 6.5 Hz, 2H), 1.90 – 1.78 (m, 1H), 0.84 (dd, *J* = 6.7, 0.6 Hz, 6H).

^{13}C{^{1}H} NMR (126 MHz, CD_3CN) δ (ppm): 173.7, 153.0, 140.6, 136.7, 129.4, 128.6, 127.1, 115.4, 115.0, 71.3, 59.3, 55.8, 39.1, 27.7, 19.1.

IR (film), $\bar{\nu}_{max}$ (cm^{-1}): 3375, 2950, 1734, 1513, 1459, 1239, 1180, 1036, 995, 821, 739, 700, 669.

HRMS (ESI-TOF) m/z calcd. for $C_{20}H_{26}NO_3^+$ (M+H) 328.1907, found 328.1903.

Enantiomeric ratio, er: 15:85 [HPLC: Daicel Chiralpak IA chiral analytical column (250 × 4.6 mm i.d.), λ 254 nm, hexane/^{i}PrOH = 80:20, flow rate 1.0 mL·min^{-1}, 25 °C, tr (major) = 8.7 min, tr (minor) = 6.5 min].

Specific rotation, $[\alpha]_D^{24}$: –8.9 (*c* 0.03, $CHCl_3$).

Compound 3fa

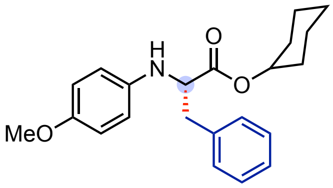

cyclohexyl (4-methoxyphenyl)-*L*-phenylalaninate
Chemical Formula: $C_{22}H_{27}NO_3$
Exact Mass: 353,1991
Molecular Weight: 353,4620

Following General Procedure (F) with **1f** (1.0 equiv., 0.05 mmol), **2a** (1.1 equiv., 0.055 mmol), and Λ-RhS (10 mol%), flow rate of 0.333 mL·min^{-1} (τ = 30 min), blue LED (440 nm, 60 W, 75% power), at 25 °C, afforded 6.0 mg (34% yield) of the title compound after purification by automated flash chromatography (25 g cartridge, using a gradient elution system hexanes:ethyl acetate, 95:5–90:10).

Physical properties: light yellow liquid.

TLC, R_f: 0.64 (hexanes:ethyl acetate, 80:20; UV active).

^{1}H NMR (500 MHz, CD_3CN) δ (ppm): 7.32 – 7.21 (m, 5H), 6.75 – 6.70 (m, 2H), 6.60 – 6.56 (m, 2H), 4.68 – 4.61 (m, 1H), 4.37 (d, *J* = 8.9 Hz, 1H), 4.23 – 4.16 (m, 1H), 3.67 (s, 3H), 3.04 (dd, *J* = 7.0, 3.2 Hz, 2H), 1.73 – 1.63 (m, 2H), 1.61 – 1.53 (m, 2H), 1.50 – 1.43 (m, 1H), 1.39 – 1.20 (m, 5H);

^{13}C{^{1}H} NMR (126 MHz, CD_3CN) δ (ppm): 173.9, 153.3, 142.3, 138.4, 130.3, 129.2, 127.6, 115.6, 73.6, 60,0, 56.1, 39.4, 32.1, 31.9, 26.0, 24.1, 24.0;

IR (film), $\bar{\nu}_{max}$ (cm^{-1}): 3373, 2931, 2855, 1732, 1515, 1457, 1241, 1195, 1039, 823, 739, 701;

HRMS (ESI-TOF) m/z calcd. for $C_{22}H_{28}NO_3^+$ (M+H) 354,2064, found 354,2059.

Enantiomeric ratio, er: 20:80 [HPLC: Daicel Chiralpak IA chiral analytical column (250 × 4.6 mm i.d.), λ 280 nm, hexane/^{i}PrOH = 80:20, flow rate 1.0 mL·min^{-1}, 25 °C, tr (major) = 9.4 min, tr (minor) = 7.2 min].

Specific rotation, $[\alpha]_D^{24}$: +9.9 (*c* 0.05, $CHCl_3$).

Compound 3ga

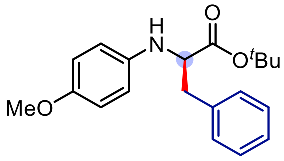

tert-butyl (4-methoxyphenyl)-*D*-phenylalaninate
Chemical Formula: $C_{20}H_{25}NO_3$
Exact Mass: 327,18344
Molecular Weight: 327,42400

Following General Procedure (F) with **1g** (1.0 equiv., 0.05 mmol), **2a** (1.1 equiv.), and Δ-RhS (10 mol%), flow rate of 0.111 mL·min^{-1} (τ = 90 min), blue LED (440 nm, 60 W, 75% power), at 25 °C, afforded 12.3 mg (75% yield) of the title compound after purification by automated flash chromatography (25 g cartridge, using a gradient elution system hexanes:ethyl acetate, 98:02–95:05).

Physical properties: light yellow liquid.

TLC, R_f: 0.60 (hexanes:ethyl acetate, 85:15; UV active).

^{1}H NMR (500 MHz, $CDCl_3$) δ (ppm): 7.32 – 7.26 (m, 2H), 7.25 – 7.19 (m, 3H), 6.79 – 6.72 (m, 2H), 6.61 – 6.55 (m, 2H), 4.15 (t, *J* = 6.5 Hz, 1H), 3.73 (s, 3H), 3.06 (h, *J* = 7.1 Hz, 2H).

^{13}C{^{1}H} NMR (126 MHz, $CDCl_3$) δ (ppm): 172.8, 152.8, 140.9, 137.0, 129.6, 128.5, 126.9, 115.4, 115.0, 81.7, 59.5, 55.9, 39.0, 28.1.

IR (film), $\bar{\nu}_{max}$ (cm^{-1}): 3380, 2985, 2925, 1729, 1514, 1456, 1368, 1240, 1151, 1036, 981, 820, 739, 701.

Enantiomeric ratio, er: 93:07 [HPLC: Daicel Chiralpak IA chiral analytical column (250 × 4.6 mm i.d.), λ 310 nm, hexane/^{i}PrOH = 80:20, flow rate 1.0 mL·min^{-1}, 25 °C, tr (major) = 4.8 min, tr (minor) = 6.7 min].

Specific rotation, $[\alpha]_D^{24}$: –12.1 (*c* 0.05, $CHCl_3$).

Compound 3gb

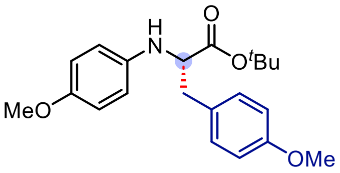

tert-butyl (*S*)-3-(4-methoxyphenyl)-2-((4-methoxyphenyl)amino)propanoate
Chemical Formula: $C_{21}H_{27}NO_4$
Exact Mass: 357,19401
Molecular Weight: 357,45000

Following General Procedure (F) with **1g** (1.0 equiv., 0.025 mmol), **2b** (1.1 equiv.), and Λ-RhS (10 mol%), flow rate of 0.111 mL·min^{-1} (τ = 90 min), blue LED (440 nm, 60 W, 75% power), at 25 °C, afforded 6.0 mg (67% yield) of the title compound after purification by automated flash chromatography (25 g cartridge, using a gradient elution system hexanes:ethyl acetate, 95:05–90:10).

Physical properties: light yellow liquid.

TLC, R_f : 0.60 (hexanes:ethyl acetate, 80:20; UV active).

^{1}H NMR (600 MHz, $CDCl_3$) δ (ppm): δ 7.14 – 7.11 (m, 2H), 6.84 – 6.80 (m, 2H), 6.77 – 6.73 (m, 2H), 6.59 – 6.55 (m, 2H), 4.11 (t, *J* = 6.4 Hz, 1H), 3.79 (s, 3H), 3.73 (s, 3H), 3.01 (d, *J* = 6.4 Hz, 2H), 1.34 (s, 9H).

$^{13}C\{^1H\}$ NMR (151 MHz, $CDCl_3$) δ (ppm): 172.8, 158.6, 152.8, 141.0, 130.6, 128.9, 115.4, 114.9, 113.9, 81.7, 59.6, 55.9, 55.4, 38.0, 28.1.

IR (film), $\bar{\nu}_{max}$ (cm^{-1}): 3895, 2923, 2825, 1728, 1613, 1512, 1466, 1368, 1304, 1245, 1153, 1037, 822.

HRMS (ESI-TOF) m/z calcd. for $C_{21}H_{28}NO_4^+$ (M+H) 358.2013, found 358.2008.

Enantiomeric ratio, er: 06:94 [HPLC: Daicel Chiralpak IB chiral analytical column (250 × 4.6 mm i.d.), λ 228 nm, hexane/^{i}PrOH = 90:10, flow rate 1.0 mL·min^{-1}, 25 °C, tr (major) = 5.6 min, tr (minor) = 5.4 min].

Specific rotation, $[\alpha]_D^{22}$: –11.4 (*c* 0.03, $CHCl_3$).

Compound 3gc

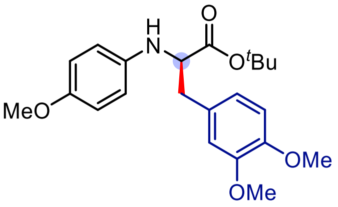

tert-butyl (*R*)-3-(3,4-dimethoxyphenyl)-2-((4-methoxyphenyl)amino)propanoate
Chemical Formula: $C_{22}H_{29}NO_5$
Exact Mass: 387,20457
Molecular Weight: 387,47600

Following General Procedure (F) with **1g** (1.0 equiv., 0.020 mmol), **2c** (1.1 equiv.), and Δ-RhS (10 mol%), flow rate of 0.056 mL·min^{-1} (τ = 180 min, a longer residence time was required for this transformation, attributed to the darker coloration and higher optical density of the reaction mixture), blue LED (440 nm, 60 W, 75% power), at 25 °C, afforded 5.4 mg (70% yield) of the title compound after purification by automated flash chromatography (25 g cartridge, using a gradient elution system hexanes:ethyl acetate, 95:05–85:15).

Physical properties: light yellow liquid.

TLC, R_f: 0.39 (hexanes:ethyl acetate, 80:20; UV active).

^{1}H NMR (300 MHz, $CDCl_3$) δ (ppm): 6.86 – 6.68 (m, 5H), 6.58 (d, *J* = 8.9 Hz, 2H), 4.13 (t, *J* = 6.3 Hz, 1H), 3.86 (s, 3H), 3.84 (s, 3H), 3.74 (d, *J* = 0.9 Hz, 3H), 3.03 (d, *J* = 6.3 Hz, 2H), 1.35 (s, 9H).

^{13}C{^{1}H} NMR (126 MHz, $CDCl_3$) δ (ppm): 172.8, 152.8, 148.9, 148.1, 140.9, 129.4, 121.8, 115.4, 115.0, 112.9, 111.2, 81.7, 59.5, 56.1, 56.0, 55.9, 38.4, 28.1.

IR (film), $\bar{\nu}_{max}$ (cm^{-1}): 3380, 2931, 2831, 1728, 1592, 1513, 1463, 1368, 1261, 1238, 1154, 1030, 822, 736.

HRMS (ESI-TOF) m/z calcd. for $C_{22}H_{30}NO_5^+$ (M+H) 388.2119, found 388.2114.

Enantiomeric ratio, er: 94:06 [HPLC: Daicel Chiralpak IB chiral analytical column (250 × 4.6 mm i.d.), λ 315 nm, hexane/^{i}PrOH = 90:10, flow rate 1.0 mL·min^{-1}, 25 °C, tr (major) = 8.1 min, tr (minor) = 9.8 min].

Specific rotation, $[\alpha]_D^{24}$: –2.3 (*c* 0.03, $CHCl_3$).

Compound 3gd

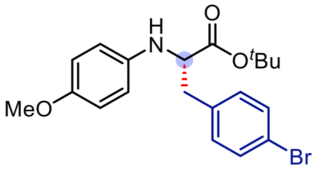

tert-butyl (*S*)-3-(4-bromophenyl)-2-((4-methoxyphenyl)amino)propanoate
Chemical Formula: $C_{20}H_{24}BrNO_3$
Exact Mass: 405,09396
Molecular Weight: 406,32000

Following General Procedure (F) with **1g** (1.0 equiv., 0.025 mmol), **2d** (1.1 equiv.), and Λ-RhS (10 mol%), flow rate of 0.111 mL·min^{-1} (τ = 90 min), blue LED (440 nm, 60 W, 75% power), at 25 °C, afforded 6.1 mg (60% yield) of the title compound after purification by automated flash chromatography (25 g cartridge, using a gradient elution system hexanes:ethyl acetate, 93:03–90:10 then hexanes:dichloromethane, 30:70–10:90).

Physical properties: light yellow liquid.

TLC, R_f : 0.50 (hexanes:dichloromethane, 50:50; UV active).

^{1}H NMR (300 MHz, $CDCl_3$) δ (ppm): 7.46 – 7.34 (m, 2H), 7.19 – 7.04 (m, 2H), 6.82 – 6.70 (m, 2H), 6.70 – 6.52 (m, 2H), 4.17 – 4.06 (m, 1H), 3.85 (s, 1H), 3.74 (s, 3H), 3.02 (d, *J* = 6.2 Hz, 2H), 1.34 (s, 9H).

^{13}C{^{1}H} NMR (126 MHz, $CDCl_3$) δ (ppm): 172.4, 152.9, 140.7, 136.0, 131.5, 131.4, 120.9, 115.4, 115.0, 82.0, 59.3, 55.9, 38.3, 28.1.

IR (film), $\bar{\nu}_{max}$ (cm^{-1}): 3400, 2979, 2924, 1730, 1513, 1487, 1368, 1240, 1151, 1072, 1039, 1011, 820, 744.

HRMS (ESI-TOF) m/z calcd. for $C_{20}H_{25}BrNO_4^+$ (M+H) 406.1012, found 406.1009.

Enantiomeric ratio, er: 14:86 [HPLC: Daicel Chiralpak IB chiral analytical column (250 × 4.6 mm i.d.), λ 310 nm, hexane/^{i}PrOH = 90:10, flow rate 1.0 mL·min^{-1}, 25 °C, tr (major) = 5.4 min, tr (minor) = 4.9 min].

Specific rotation, $[\alpha]_D^{24}$: –0.1 (*c* 0.05, $CHCl_3$).

Compound 3ge

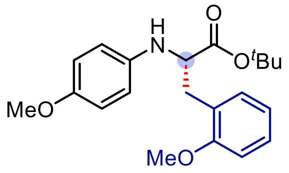

tert-butyl (*S*)-3-(2-methoxyphenyl)-2-((4-methoxyphenyl)amino)propanoate
Chemical Formula: $C_{21}H_{27}NO_4$
Exact Mass: 357,19401
Molecular Weight: 357,45000

Following General Procedure (F) with **1g** (1.0 equiv., 0.025 mmol), **2e** (1.1 equiv.), and Λ-RhS (10 mol%), flow rate of 0.111 mL·min^{-1} (τ = 90 min), blue LED (440 nm, 60 W, 75% power), at 25 °C, afforded 4.7 mg (53% yield) of the title compound after purification by automated flash chromatography (25 g cartridge, using a gradient elution system hexanes:ethyl acetate, 97:03–90:10).

Physical properties: light yellow liquid.

TLC, R_f: 0.30 (hexanes:ethyl acetate, 85:15; UV active).

^{1}H NMR (500 MHz, $CDCl_3$) δ (ppm): 7.21 (td, *J* = 7.9, 1.7 Hz, 1H), 7.13 (dd, *J* = 7.4, 1.2 Hz, 1H), 6.87 (t, *J* = 7.4 Hz, 2H), 6.77 – 6.72 (m, 2H), 6.61 – 6.56 (m, 2H), 4.22 (t, *J* = 7.3 Hz, 1H), 4.07 (s, 1H), 3.87 (s, 3H), 3.73 (s, 3H), 3.22 (dd, *J* = 13.4, 7.1 Hz, 1H), 2.88 (dd, *J* = 13.4, 7.4 Hz, 1H), 1.28 (s, 9H).

^{13}C{^{1}H} NMR (126 MHz, $CDCl_3$) δ (ppm): 173.4, 157.9, 152.5, 141.3, 131.3, 128.2, 125.9, 120.6, 115.0, 114.9, 110.4, 81.3, 58.4, 55.9, 55.4, 34.6, 28.0.

IR (film), $\bar{\nu}_{max}$ (cm^{-1}): 3381, 2929, 1726, 1595, 1514, 1496, 1464, 1368, 1295, 1243, 1154, 1033, 822, 754, 665.

HRMS (ESI-TOF) m/z calcd. for $C_{21}H_{28}NO_4^+$ (M+H) 358.2013, found 358.2009.

Enantiomeric ratio, er: 09:91 [HPLC: Daicel Chiralpak IB chiral analytical column (250 × 4.6 mm i.d.), λ 310 nm, hexane/^{i}PrOH = 90:10, flow rate 1.0 mL·min^{-1}, 25 °C, tr (major) = 7.4 min, tr (minor) = 6.3 min].

Specific rotation, $[\alpha]_D^{23}$: –25.8 (*c* 0.04, $CHCl_3$).

Compound 3gf

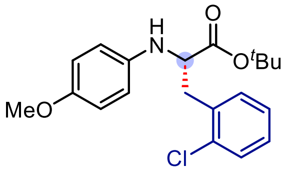

tert-butyl (*S*)-3-(2-chlorophenyl)-2-((4-methoxyphenyl)amino)propanoate
Chemical Formula: $C_{20}H_{24}ClNO_3$
Exact Mass: 361,14447
Molecular Weight: 361,86600

Following General Procedure (F) with **1g** (1.0 equiv., 0.025 mmol), **2f** (1.1 equiv.), and Λ-RhS (10 mol%), flow rate of 0.111 mL·min^{-1} (τ = 90 min), blue LED (440 nm, 60 W, 75% power), at 25 °C, afforded 4.5 mg (50% yield) of the title compound after purification by automated flash chromatography (25 g cartridge, using a gradient elution system hexanes:ethyl acetate, 95:05–90:10).

Physical properties: light yellow liquid.

TLC, R_f: 0.34 (hexanes:ethyl acetate, 85:15; UV active).

^{1}H NMR (300 MHz, $CDCl_3$) δ (ppm): 7.41 – 7.33 (m, 1H), 7.25 (s, 1H), 7.22 – 7.14 (m, 2H), 6.77 – 6.71 (m, 2H), 6.62 – 6.56 (m, 2H), 4.26 (t, *J* = 7.4 Hz, 1H), 3.95 (s, 1H), 3.73 (s, 3H), 3.28 – 3.04 (m, 2H), 1.30 (s, 9H).

^{13}C{^{1}H} NMR (151 MHz, $CDCl_3$) δ (ppm): 172.9, 152.9, 141.0, 135.3, 134.5, 131.8, 129.7, 128.4, 126.9, 115.5, 114.9, 81.8, 58.4, 55.9, 37.5, 28.0.

IR (film), $\bar{\upsilon}_{max}$ (cm^{-1}): 2926, 2854, 1734, 1513, 1449, 1369, 1246, 1151, 1036, 973, 837, 815, 751, 669.

HRMS (ESI-TOF) m/z calcd. for $C_{20}H_{25}ClNO_3^+$ (M+H) 362.1518, found 362.1512.

Enantiomeric ratio, er: 11:89 [HPLC: Daicel Chiralpak IB chiral analytical column (250 × 4.6 mm i.d.), λ 310 nm, hexane/^{i}PrOH = 90:10, flow rate 1.0 mL·min^{-1}, 25 °C, tr (major) = 5.0 min, tr (minor) = 4.7 min].

Specific rotation, $[\alpha]_D^{23}$: –7.2 (*c* 0.04, $CHCl_3$).

Compound 3gg

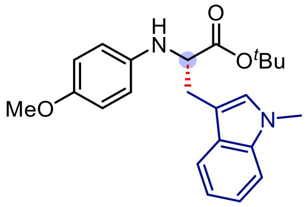

tert-butyl N^{α}-(4-methoxyphenyl)-1-methyl-*L*-tryptophanate
Chemical Formula: $C_{23}H_{28}N_2O_3$
Exact Mass: 380,20999
Molecular Weight: 380,48800

Following General Procedure (F) with **1g** (1.0 equiv., 0.025 mmol), **2g** (1.1 equiv.), and Λ-RhS (10 mol%), flow rate of 0.111 mL·min^{-1} (τ = 90 min), blue LED (440 nm, 60 W, 75% power), at 25 °C, afforded 5.5 mg (58% yield) of the title compound after purification by automated flash chromatography (25 g cartridge, using a gradient elution system hexanes:ethyl acetate, 95:05–90:10).

Physical properties: light yellow liquid.

TLC, R_f: 0.18 (hexanes:ethyl acetate, 85:15; UV active).

^{1}H NMR (500 MHz, $CDCl_3$) δ (ppm): 7.61 (d, *J* = 7.9 Hz, 1H), 7.28 (d, *J* = 8.2 Hz, 1H), 7.24 – 7.20 (m, 1H), 7.13 – 7.08 (m, 1H), 6.92 (s, 1H), 6.76 – 6.71 (m, 2H), 6.58 – 6.54 (m, 2H), 4.21 (t, *J* = 6.2 Hz, 1H), 3.96 (s, 1H), 3.74 (s, 3H), 3.73 (s, 3H), 3.24 (qd, *J* = 14.6, 6.2 Hz, 2H), 1.31 (s, 9H).

$^{13}C\{^1H\}$ NMR (126 MHz, $CDCl_3$) δ (ppm): 173.3, 152.7, 141.3, 137.0, 128.4, 127.7, 121.8, 119.2, 119.1, 115.2, 114.9, 109.6, 109.3, 81.5, 59.0, 55.9, 32.8, 28.6, 28.1.

IR (film), $\bar{\nu}_{max}$ (cm^{-1}): 3400, 2921, 2849, 1726, 1620, 1513, 1473, 1368, 1325, 1240, 1153, 1037, 822, 741.

HRMS (ESI-TOF) m/z calcd. for $C_{23}H_{29}N_2O_3^+$ (M+H) 381.2173, found 381.2168.

Enantiomeric ratio, er: 01:99 [HPLC: Daicel Chiralpak IB chiral analytical column (250 × 4.6 mm i.d.), λ 290 nm, hexane/^{i}PrOH = 90:10, flow rate 1.0 mL·min^{-1}, 25 °C, tr (major) = 9.5 min, tr (minor) = 8.2 min].

Specific rotation, $[\alpha]_D^{23}$: +0.6 (*c* 0.03, $CHCl_3$).

Compound 3gh

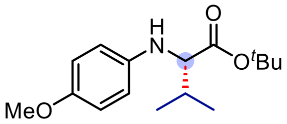

tert-butyl (4-methoxyphenyl)-*L*-valinate
Chemical Formula: $C_{16}H_{25}NO_3$
Exact Mass: 279,18344
Molecular Weight: 279,38000

Following General Procedure (F) with **1g** (1.0 equiv., 0.025 mmol), **2h** (1.1 equiv.), and Λ-RhS (10 mol%), flow rate of 0.111 mL·min^{-1} (τ = 90 min), blue LED (440 nm, 60 W, 75% power), at 25 °C, afforded 4.0 mg (57% yield) of the title compound after purification by automated flash chromatography (25 g cartridge, using a gradient elution system hexanes:ethyl acetate, 99:01–90:10).

Physical properties: light yellow liquid.

TLC, R_f: 0.39 (hexanes:ethyl acetate, 95:05; UV active).

^{1}H NMR (500 MHz, $CDCl_3$) δ (ppm): δ 6.79 – 6.72 (m, 2H), 6.65 – 6.59 (m, 2H), 3.74 (s, 3H), 3.64 (d, J = 5.8 Hz, 1H), 2.07 (dq, J = 13.5, 6.8 Hz, 1H), 1.41 (s, 9H), 1.03 (t, J = 7.2 Hz, 6H).

^{13}C{^{1}H} NMR (126 MHz, $CDCl_3$) δ (ppm): 173.2, 152.8, 115.6, 114.9, 81.5, 64.5, 55.9, 31.6, 28.2, 19.2, 18.9.

IR (film), $\bar{\upsilon}_{max}$ (cm^{-1}): 3390, 2967, 2930, 1726, 1513, 1466, 1368, 1237, 1147, 1040, 972, 821, 736.

HRMS (ESI-TOF) m/z calcd. for $C_{16}H_{26}NO_3^+$ (M+H) 280.1907, found 280.1905.

Enantiomeric ratio, er: 18:82 [HPLC: Daicel Chiralpak IA chiral analytical column (250 × 4.6 mm i.d.), λ 310 nm, hexane/^{i}PrOH = 80:20, flow rate 1.0 mL·min^{-1}, 25 °C, tr (major) = 4.3 min, tr (minor) = 3.8 min].

Specific rotation, $[\alpha]_D^{24}$: –110.2 (c 0.03, $CHCl_3$).

Compound 3gi

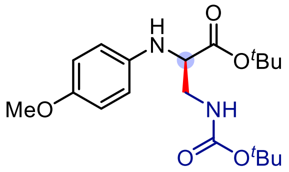

tert-butyl (*R*)-3-((*tert*-butoxycarbonyl)amino)-2-((4-methoxyphenyl)amino)propanoate
Chemical Formula: $C_{19}H_{30}N_2O_5$
Exact Mass: 366,21547
Molecular Weight: 366,45800

Following General Procedure (F) with **1g** (1.0 equiv., 0.02 mmol), **2i** (1.1 equiv.), and Δ-RhS (10 mol%), flow rate of 0.111 mL·min^{-1} (τ = 90 min), blue LED (440 nm, 60 W, 75% power), at 25 °C, afforded 4.5 mg (62% yield) of the title compound after purification by automated flash chromatography (25 g cartridge, using a gradient elution system hexanes:ethyl acetate, 95:05–85:15).

Physical properties: yellow liquid.

TLC, R_f : 0.15 (hexanes:ethyl acetate, 90:10; UV active).

^{1}H NMR (500 MHz, $CDCl_3$) δ (ppm): 6.80 – 6.74 (m, 2H), 6.65 – 6.59 (m, 2H), 4.90 (s, 1H), 3.99 (t, *J* = 5.6 Hz, 1H), 3.74 (s, 3H), 3.54 – 3.47 (m, 2H), 1.44 (s, 18H).

^{13}C{^{1}H} NMR (126 MHz, $CDCl_3$) δ (ppm): 171.7, 156.1, 153.1, 141.0, 115.4, 115.0, 82.6, 79.7, 58.7, 55.9, 42.7, 28.5, 28.1.

IR (film), $\bar{v}_{max}$ (cm^{-1}): 3401, 2926, 1717, 1520, 1457, 1369, 1243, 1162, 1065, 1030, 956, 868, 824, 741, 668.

HRMS (ESI-TOF) m/z calcd. for $C_{19}H_{31}N_2O_5^+$ (M+H) 367.2228, found 367.2224.

Enantiomeric ratio, er: 86:14 [HPLC: Daicel Chiralpak IA chiral analytical column (250 × 4.6 mm i.d.), λ 308 nm, hexane/^{i}PrOH = 80:20, flow rate 1.0 mL·min^{-1}, 25 °C, tr (major) = 5.4 min, tr (minor) = 6.7 min].

Specific rotation, $[\alpha]_D^{23}$: –17.6 (*c* 0.03, $CHCl_3$).

X - REFERENCE

(1) Batista, A. N. L.; Valverde, A. L.; Nafie, L. A.; Batista Jr, J. M. Stereochemistry of Natural Products from Vibrational Circular Dichroism. *Chemical Communications* **2024**, 60 (76), 10439–10450. https://doi.org/10.1039/D4CC02481H.

(2) Frisch, M. J.; Trucks, G. W.; Schlegel, H. B.; Scuseria, G. E.; Robb, M. A.; Cheeseman, J. R.; Scalmani, G.; Barone, V.; Mennucci, B.; Petersson, G. A. Gaussian 09, Revision A. 02; Gaussian, Inc: Wallingford, CT, 2009. *J. Mater. Chem*. **2009**.

(3) Hatano, M.; Yamashita, K.; Ishihara, K. C- and N-Selective Grignard Addition Reactions of α-Aldimino Esters in the Presence or Absence of Zinc(II) Chloride: Synthetic Applications to Optically Active Azacycles. *Org. Lett*. **2015**, 17 (10), 2412–2415. https://doi.org/10.1021/acs.orglett.5b00927.

(4) Li, G.; Chen, R.; Wu, L.; Fu, Q.; Zhang, X.; Tang, Z. Alkyl Transfer from C–C Cleavage. *Angew. Chem. Int. Ed*. **2013**, 52 (32), 8432–8436. https://doi.org/10.1002/anie.201303696.

(5) Chen, W.; Liu, Z.; Tian, J.; Li, J.; Ma, J.; Cheng, X.; Li, G. Building Congested Ketone: Substituted Hantzsch Ester and Nitrile as Alkylation Reagents in Photoredox Catalysis. *J. Am. Chem. Soc*. **2016**, 138 (38), 12312–12315. https://doi.org/10.1021/jacs.6b06379.

(6) Ma, J.; Zhang, X.; Huang, X.; Luo, S.; Meggers, E. Preparation of Chiral-at-Metal Catalysts and Their Use in Asymmetric Photoredox Chemistry. *Nat. Protoc*. **2018**, 13 (4), 605–632. https://doi.org/10.1038/nprot.2017.138.

(7) Inokuma, T.; Jichu, T.; Nishida, K.; Shigenaga, A.; Otaka, A. A Convenient Method for Preparation of α-Imino Carboxylic Acid Derivatives and Application to the Asymmetric Synthesis of Unnatural α-Amino Acid Derivative. *Chem. Pharm. Bull*. (Tokyo). **2017**, 65 (6), 573–581. https://doi.org/10.1248/cpb.c17-00158.

(8) Wu, X.; Xia, H.; Gao, C.; Luan, B.; Wu, L.; Zhang, C.; Yang, D.; Hou, L.; Liu, N.; Xia, T.; Li, H.; Qu, J.; Chen, Y. Modular α-Tertiary Amino Ester Synthesis through Cobalt-Catalysed Asymmetric Aza-Barbier Reaction. *Nat. Chem*. **2024**, 16 (3), 398–407. https://doi.org/10.1038/s41557-023-01378-9.

(9) Niwa, Y.; Shimizu, M. Tandem N-Alkylation−C-Allylation Reaction of α-Imino Esters with Organoaluminums and Allyltributyltin. *J. Am. Chem. Soc*. **2003**, 125 (13), 3720–3721. https://doi.org/10.1021/ja029639o.

(10) Li, Q.; Sun, H.; Yan, F.; Zhao, Y.; Zhang, Y.; Zhou, C.; Han, M.; Li, H.; Sui, X. Modular Construction of N -Arylated Amino Acid Esters Enabled by a Photoredox-Catalyzed Multicomponent Reaction. *Green Chemistry* **2023**, 25 (16), 6226–6230. https://doi.org/10.1039/D3GC01633A.

(11) Hatano, M.; Yamashita, K.; Ishihara, K. C- and N-Selective Grignard Addition Reactions of α-Aldimino Esters in the Presence or Absence of Zinc(II) Chloride: Synthetic Applications to Optically Active Azacycles. *Org. Lett*. **2015**, 17 (10). https://doi.org/10.1021/acs.orglett.5b00927.

XI. ^{1}H and $^{13}C\{^{1}H\}$ NMR SPECTRA

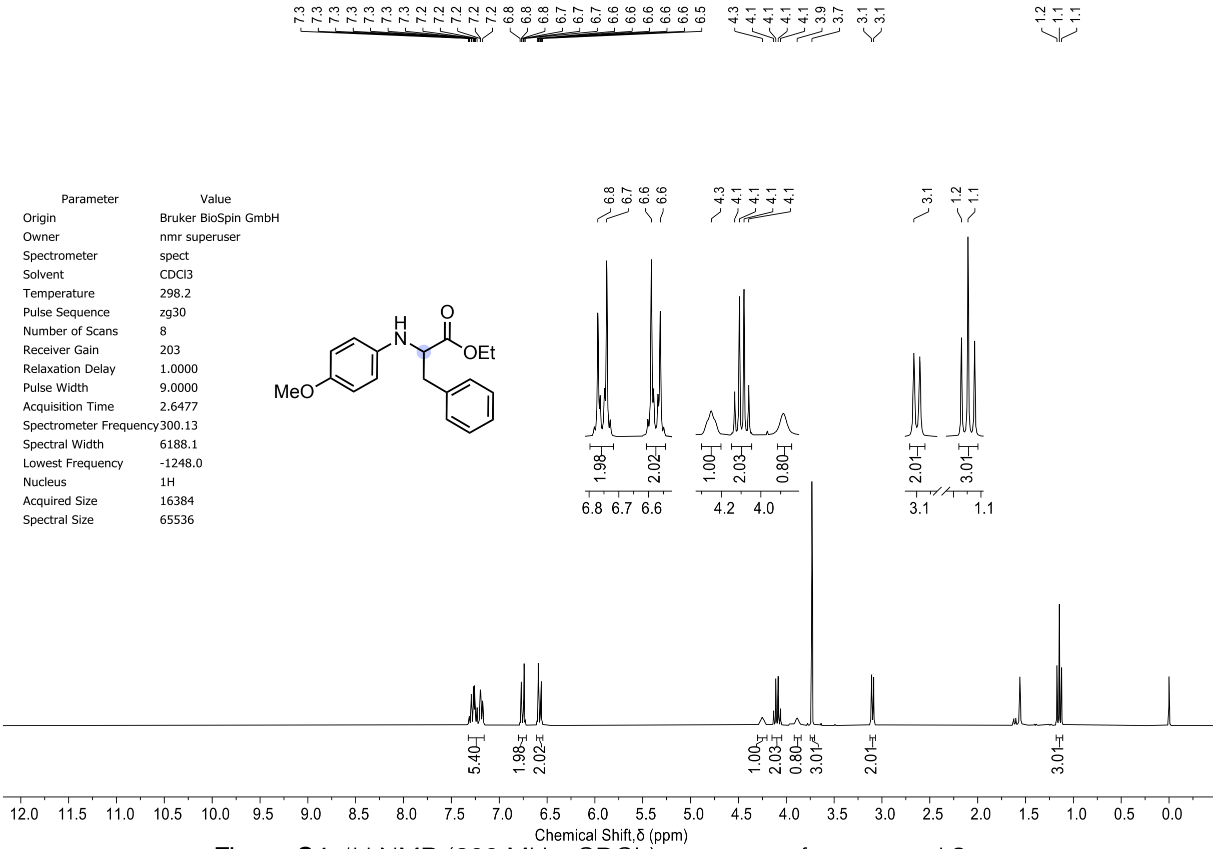

Figure S4. ^{1}H NMR (300 MHz, $CDCl_3$) spectrum of compound **3aa**.

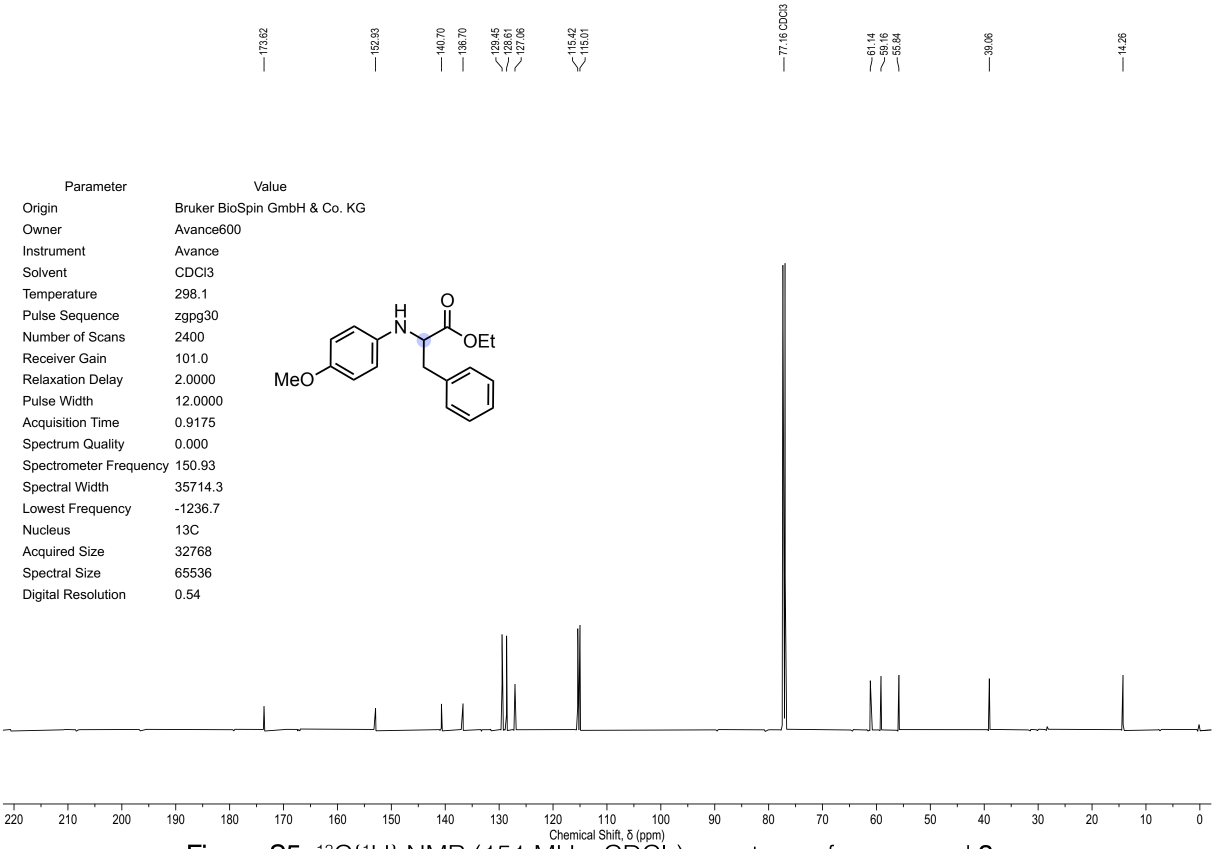

Figure S5. $^{13}C\{^{1}H\}$ NMR (151 MHz, $CDCl_3$) spectrum of compound **3aa**.

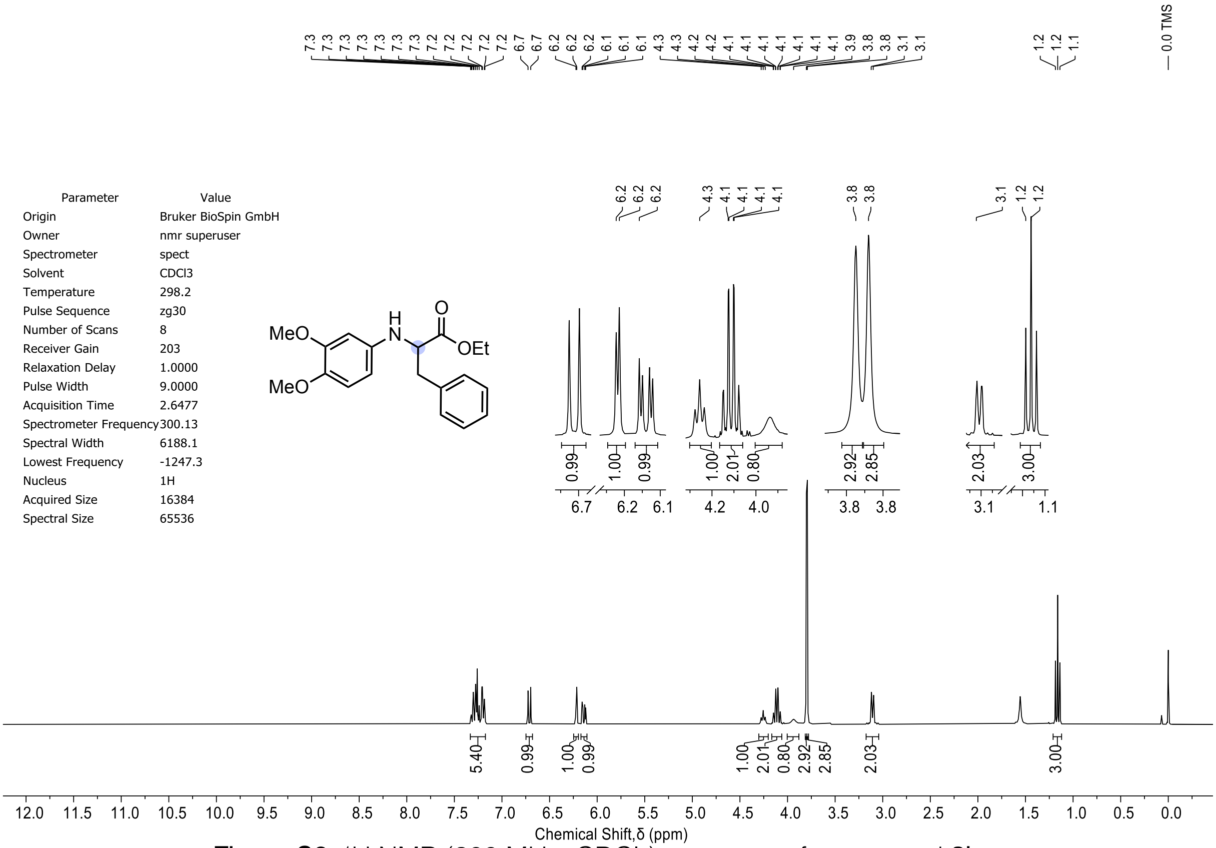

Figure S6. ^{1}H NMR (300 MHz, $CDCl_3$) spectrum of compound **3ba**.

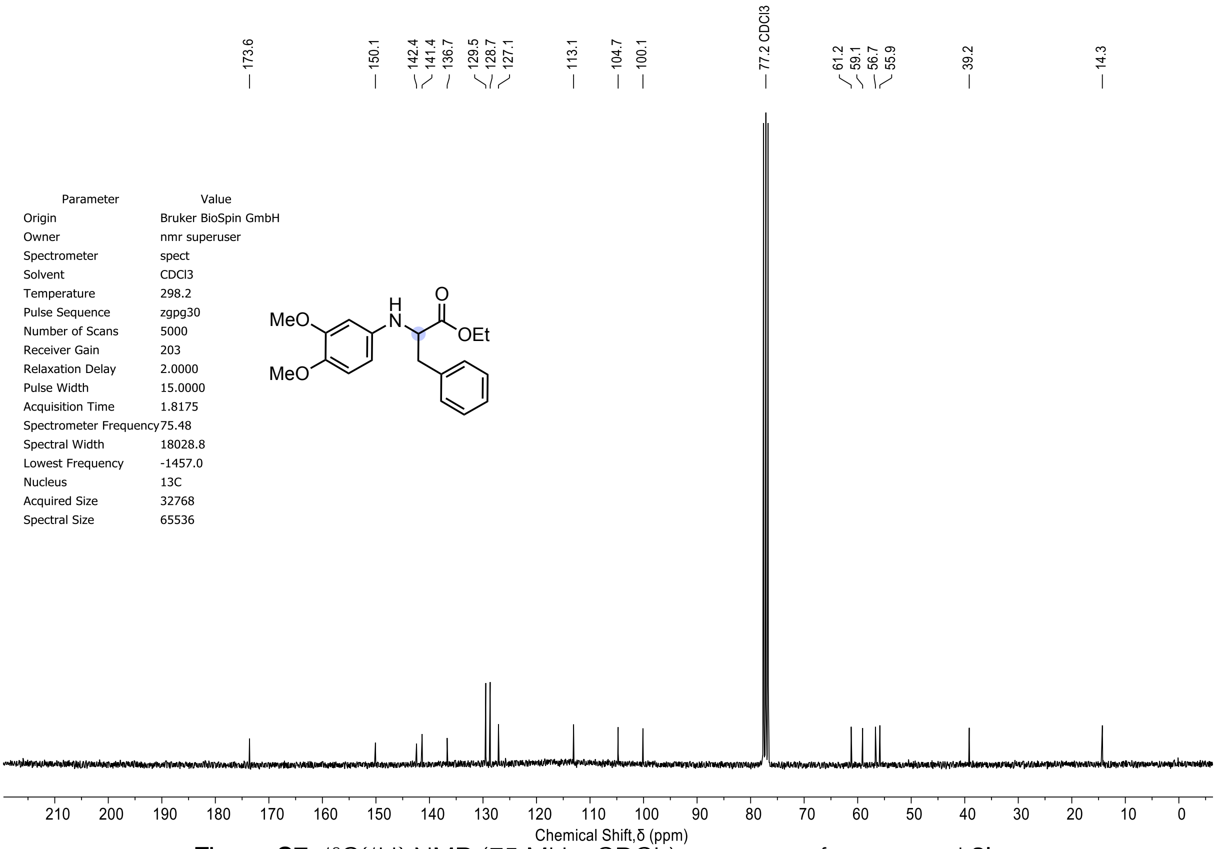

Figure S7. $^{13}C\{^{1}H\}$ NMR (75 MHz, $CDCl_3$) spectrum of compound **3ba**.

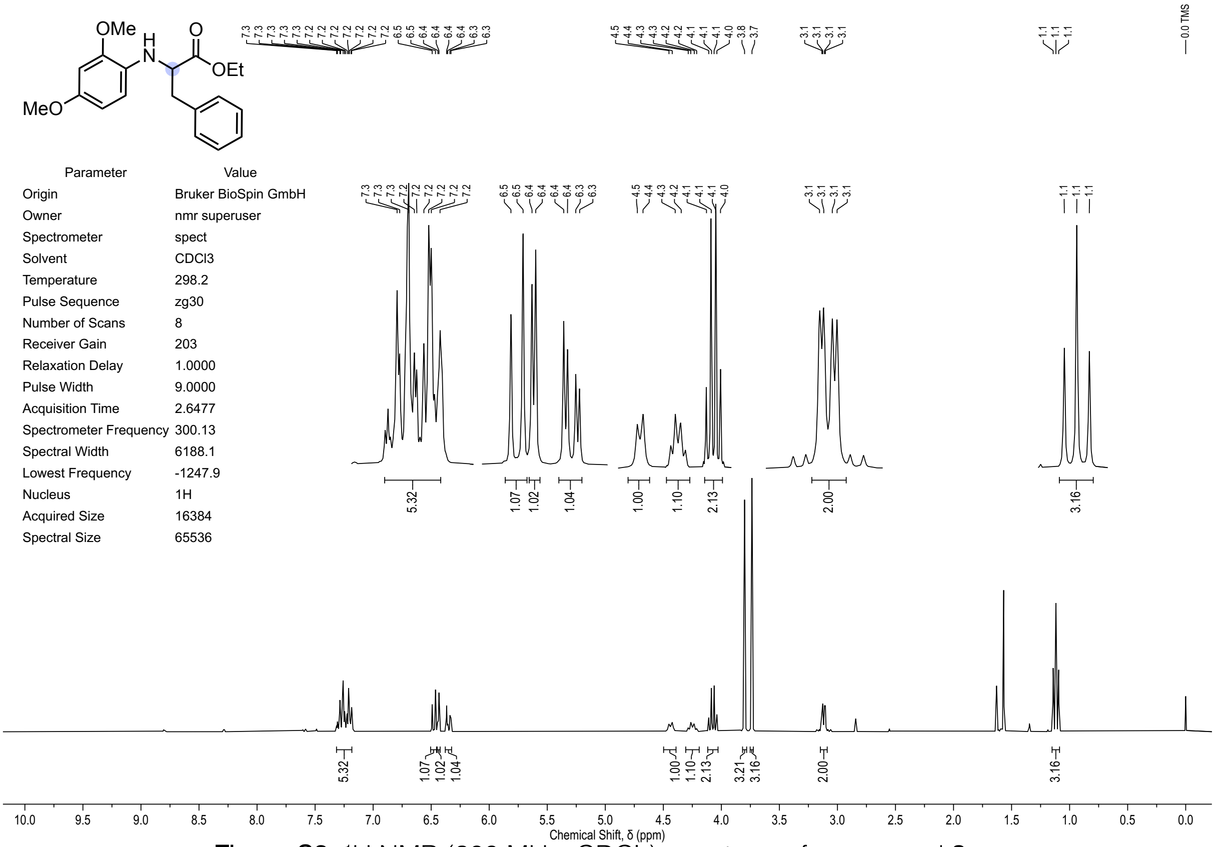

Parameter	Value
Origin	Bruker BioSpin GmbH
Owner	nmr superuser
Spectrometer	spect
Solvent	CDCl3
Temperature	298.2
Pulse Sequence	zg30
Number of Scans	8
Receiver Gain	203
Relaxation Delay	1.0000
Pulse Width	9.0000
Acquisition Time	2.6477
Spectrometer Frequency	300.13
Spectral Width	6188.1
Lowest Frequency	-1247.9
Nucleus	1H
Acquired Size	16384
Spectral Size	65536

Figure S8. ^{1}H NMR (300 MHz, $CDCl_3$) spectrum of compound **3ca**.

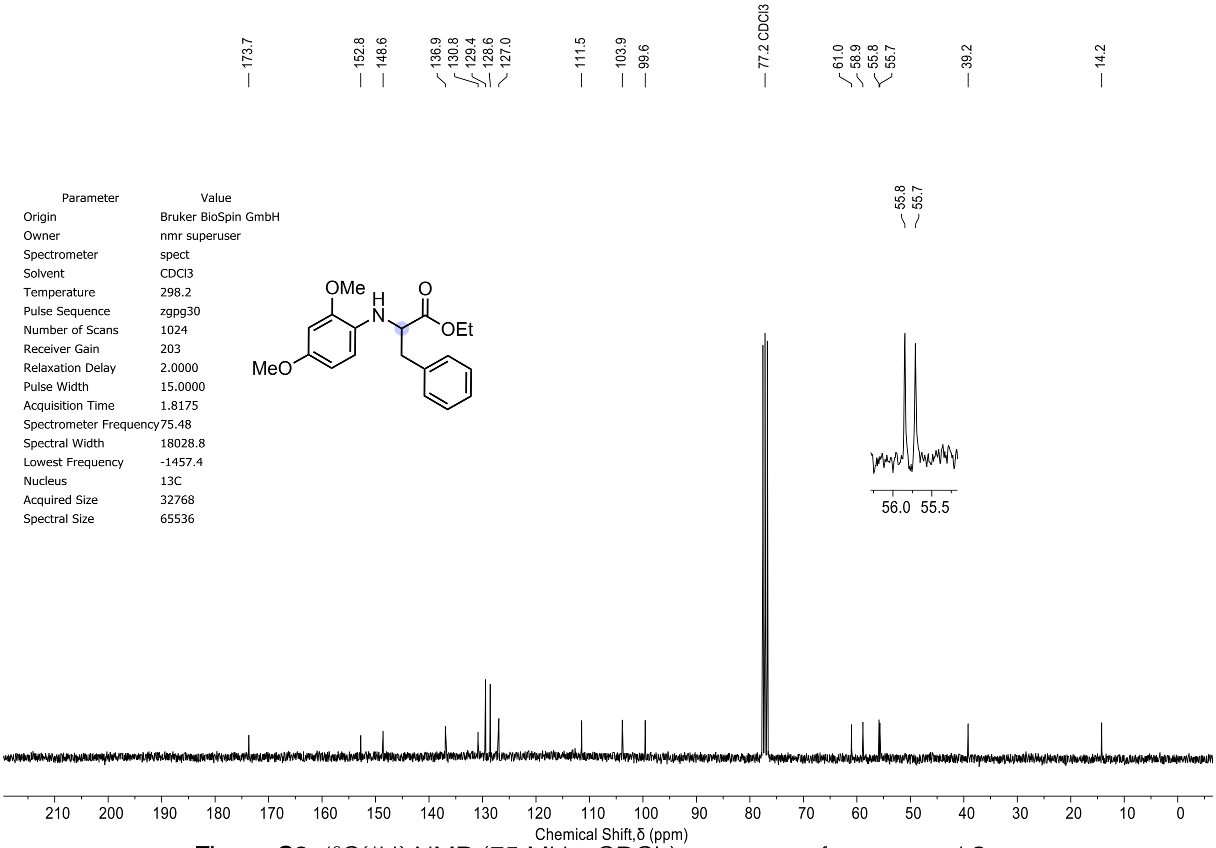

Parameter	Value
Origin	Bruker BioSpin GmbH
Owner	nmr superuser
Spectrometer	spect
Solvent	CDCl3
Temperature	298.2
Pulse Sequence	zgpg30
Number of Scans	1024
Receiver Gain	203
Relaxation Delay	2.0000
Pulse Width	15.0000
Acquisition Time	1.8175
Spectrometer Frequency	75.48
Spectral Width	18028.8
Lowest Frequency	-1457.4
Nucleus	13C
Acquired Size	32768
Spectral Size	65536

Figure S9. $^{13}C\{^{1}H\}$ NMR (75 MHz, $CDCl_3$) spectrum of compound **3ca**.

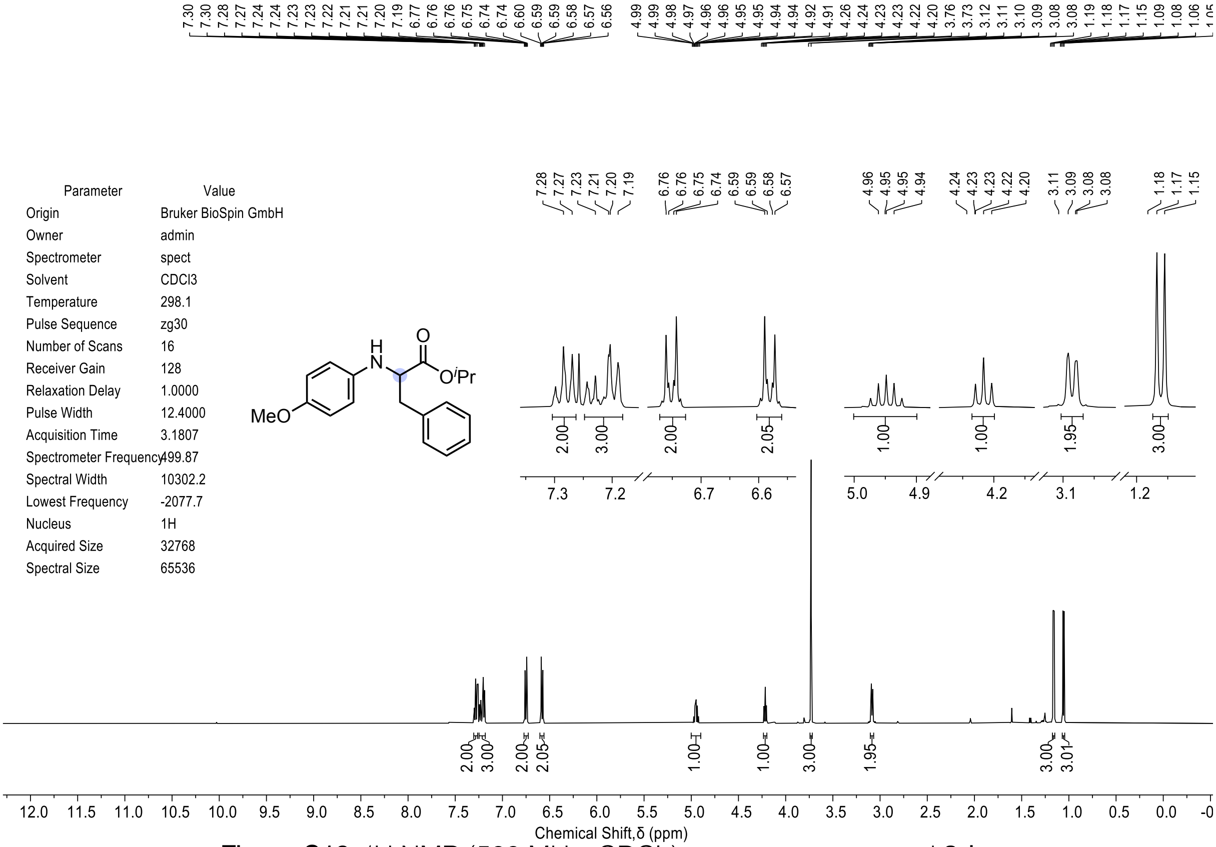

Figure S10. ^{1}H NMR (500 MHz, $CDCl_3$) spectrum compound **3da**.

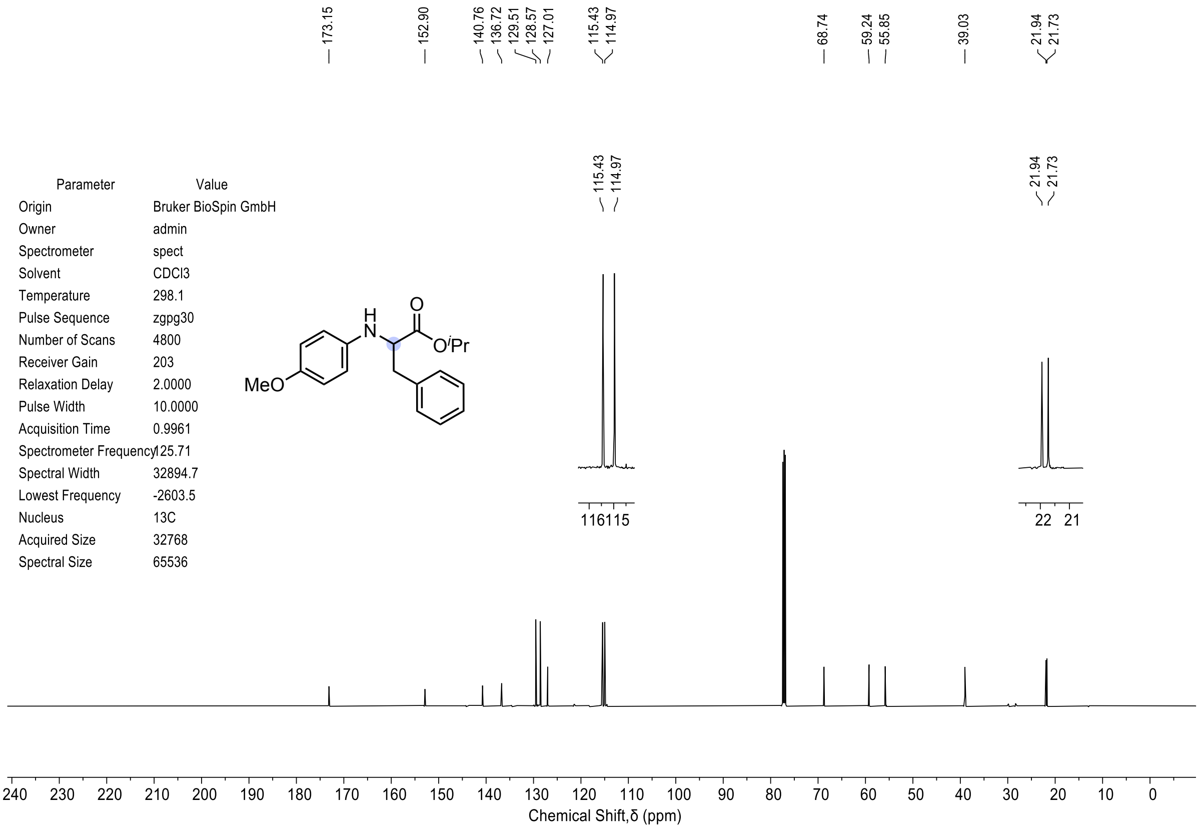

Figure S11. $^{13}C\{^{1}H\}$ NMR (126 MHz, $CDCl_3$) spectrum compound **3da**.

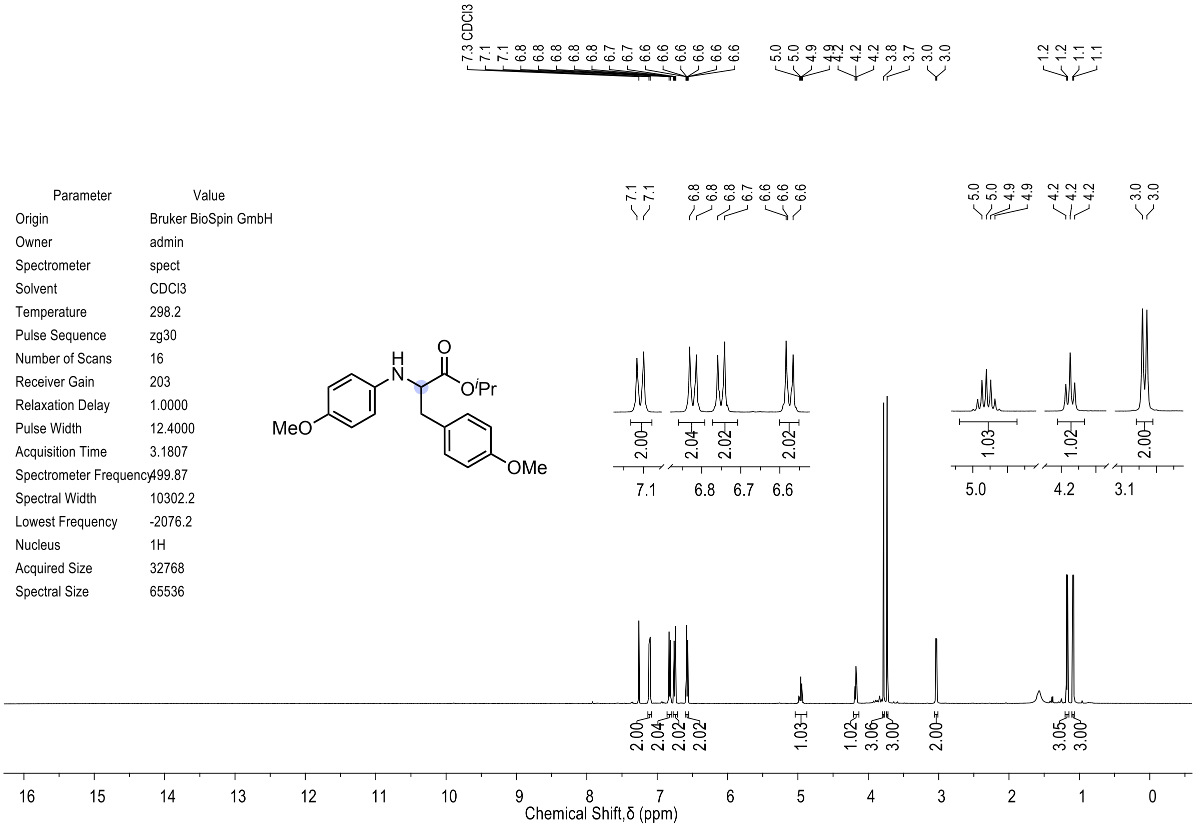

Figure S12. ^{1}H NMR (500 MHz, $CDCl_3$) spectrum of compound **3db**.

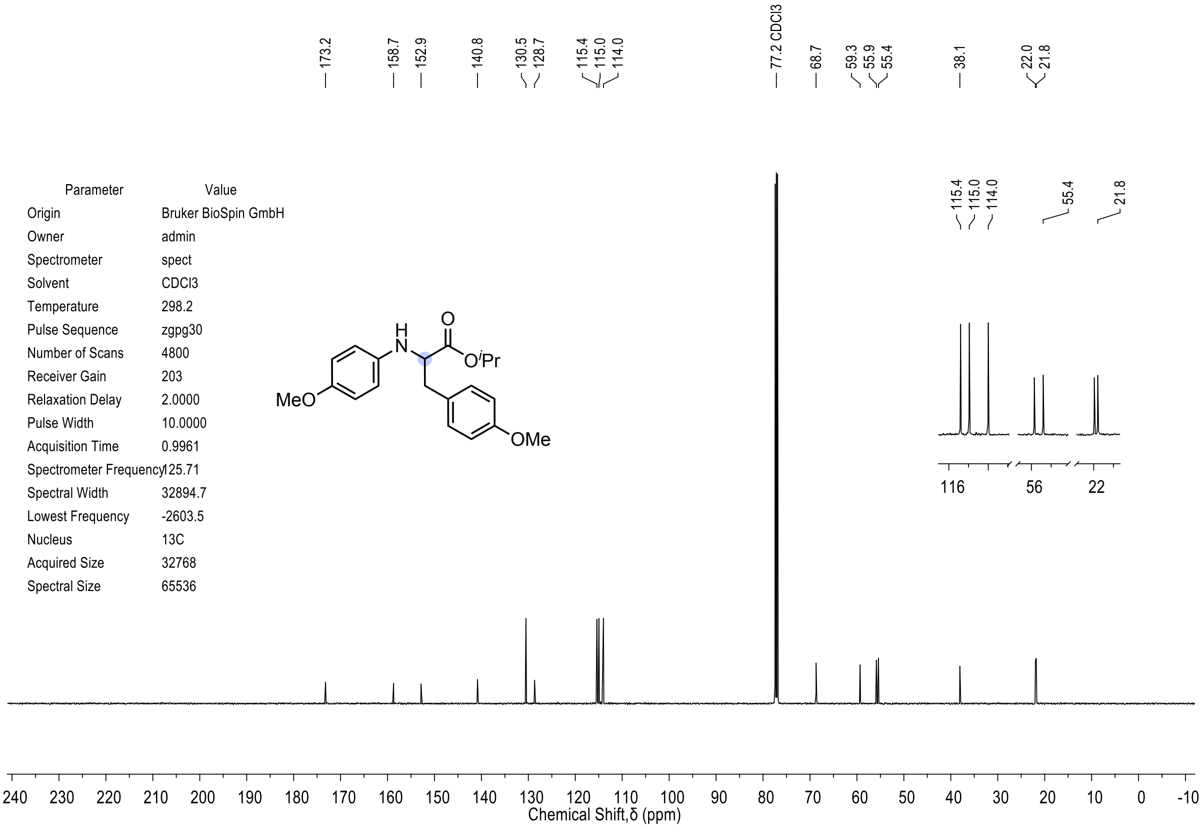

Figure S13. $^{13}C\{^1H\}$ NMR (126 MHz, $CDCl_3$) spectrum of compound **3db**.

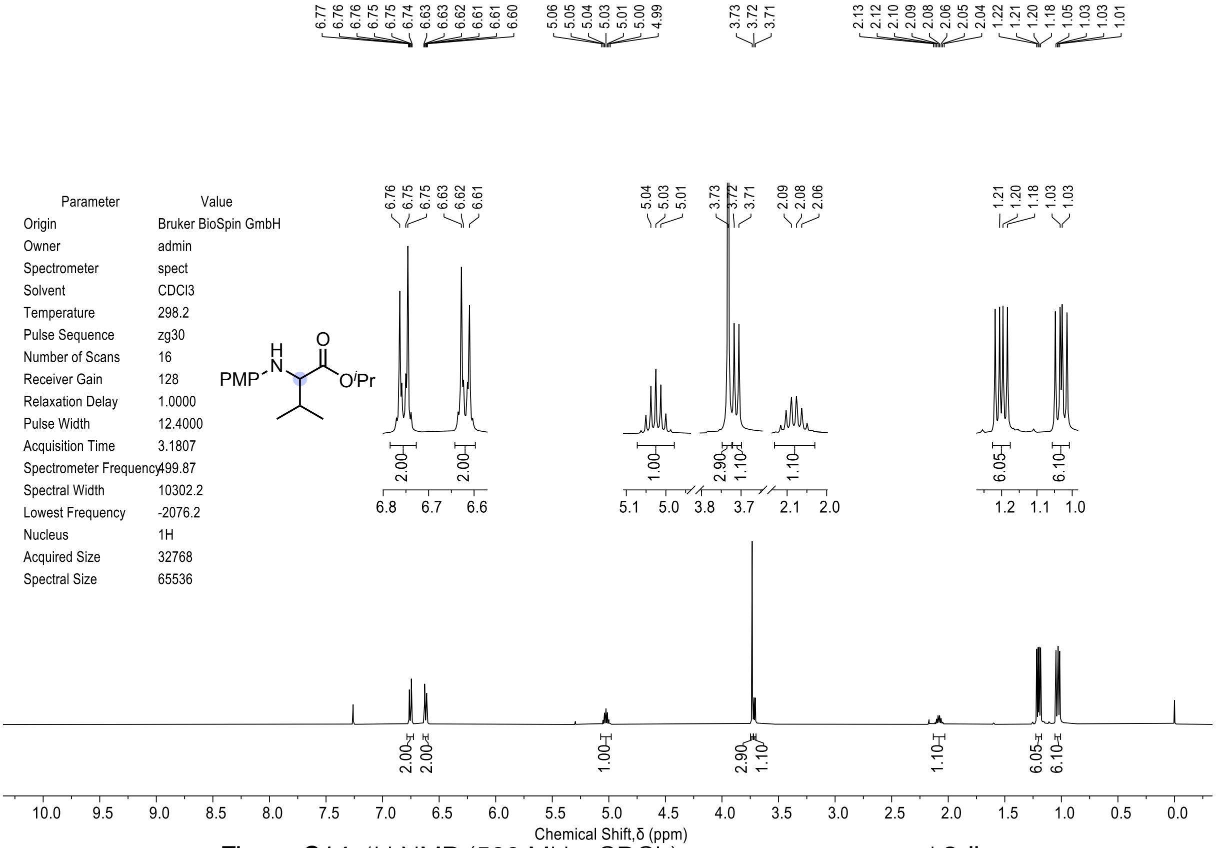

Figure S14. ^{1}H NMR (500 MHz, $CDCl_3$) spectrum compound **3dh**.

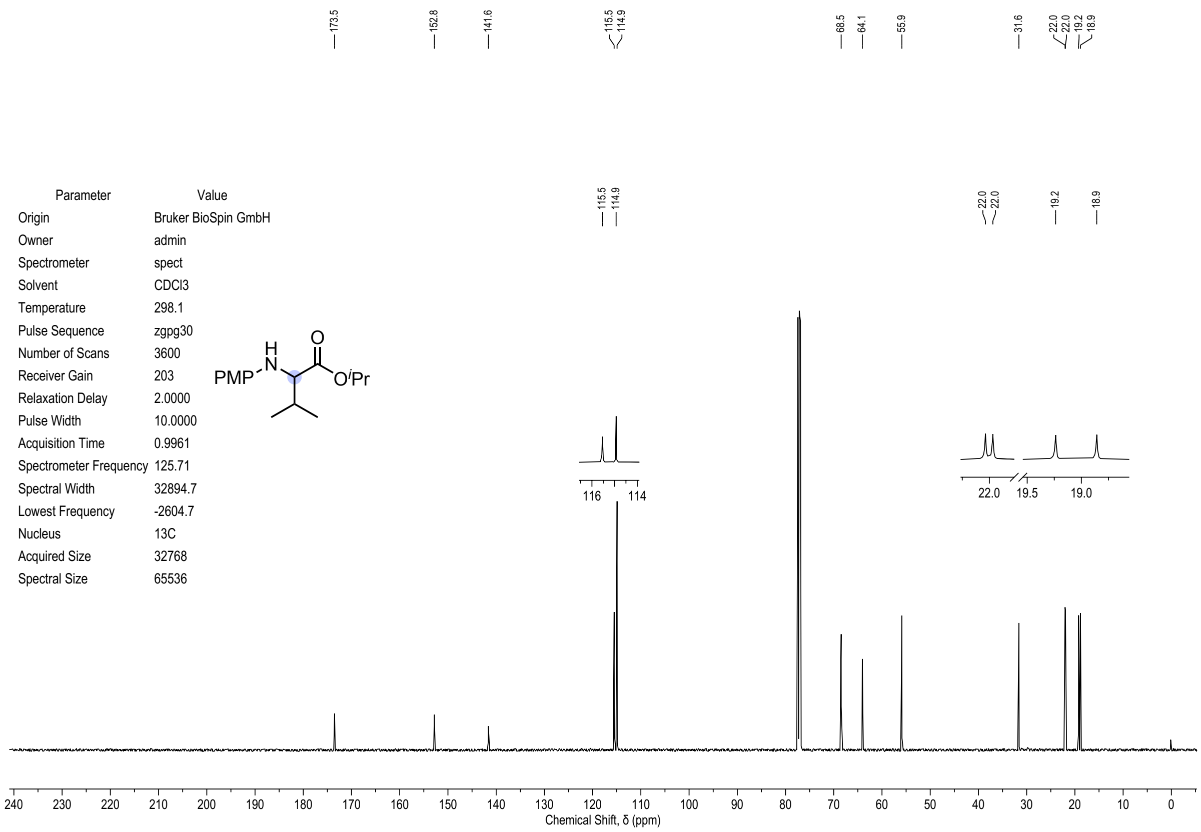

Figure S15. $^{13}C\{^1H\}$ NMR (126 MHz, $CDCl_3$) spectrum compound **3dh**.

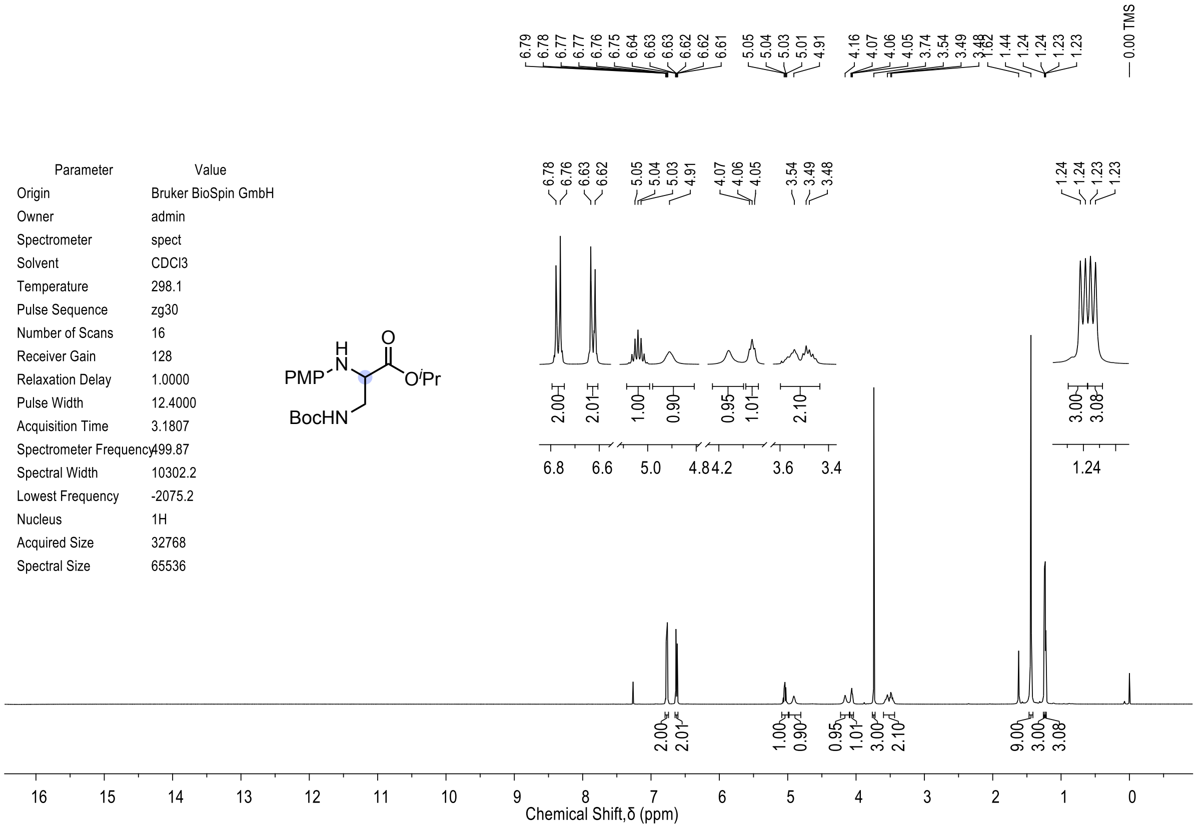

Figure S16. ^{1}H NMR (500 MHz, $CDCl_3$) spectrum compound **3di**.

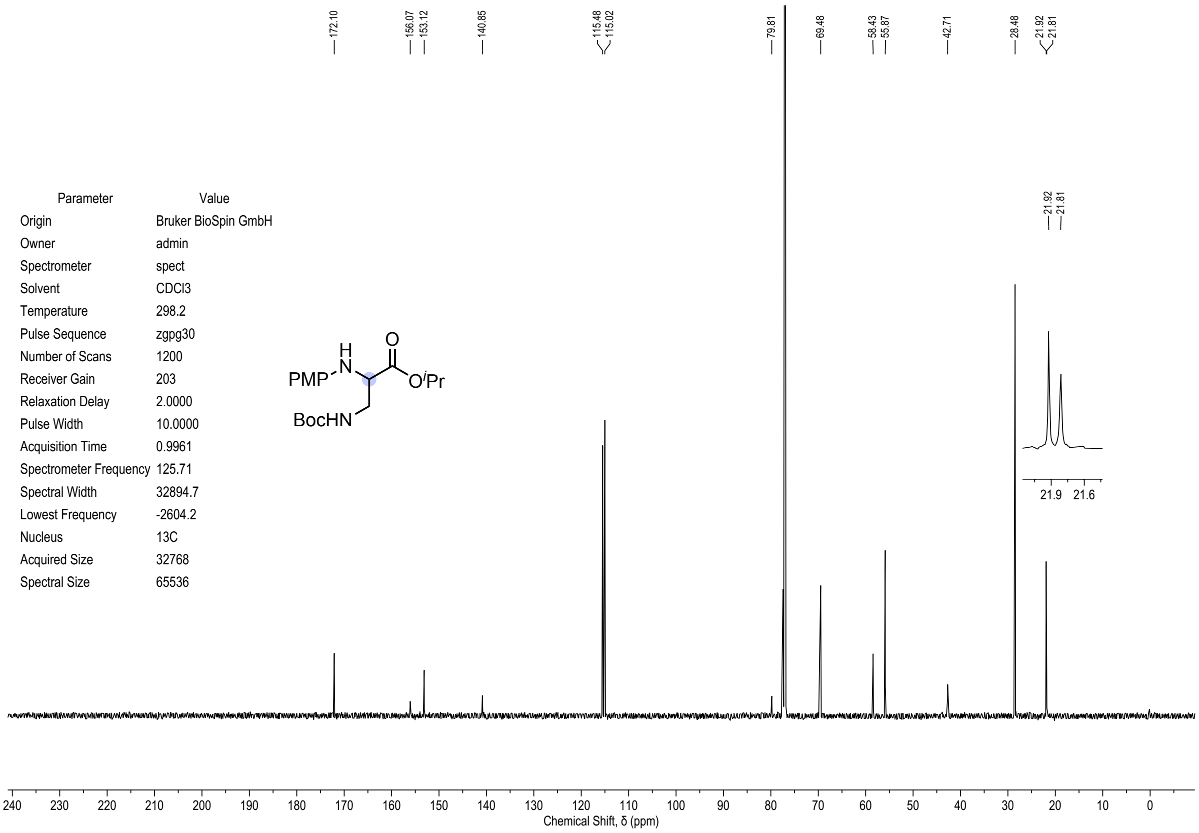

Figure S17. ^{13}C{^{1}H} NMR (126 MHz, $CDCl_3$) spectrum compound **3di**.

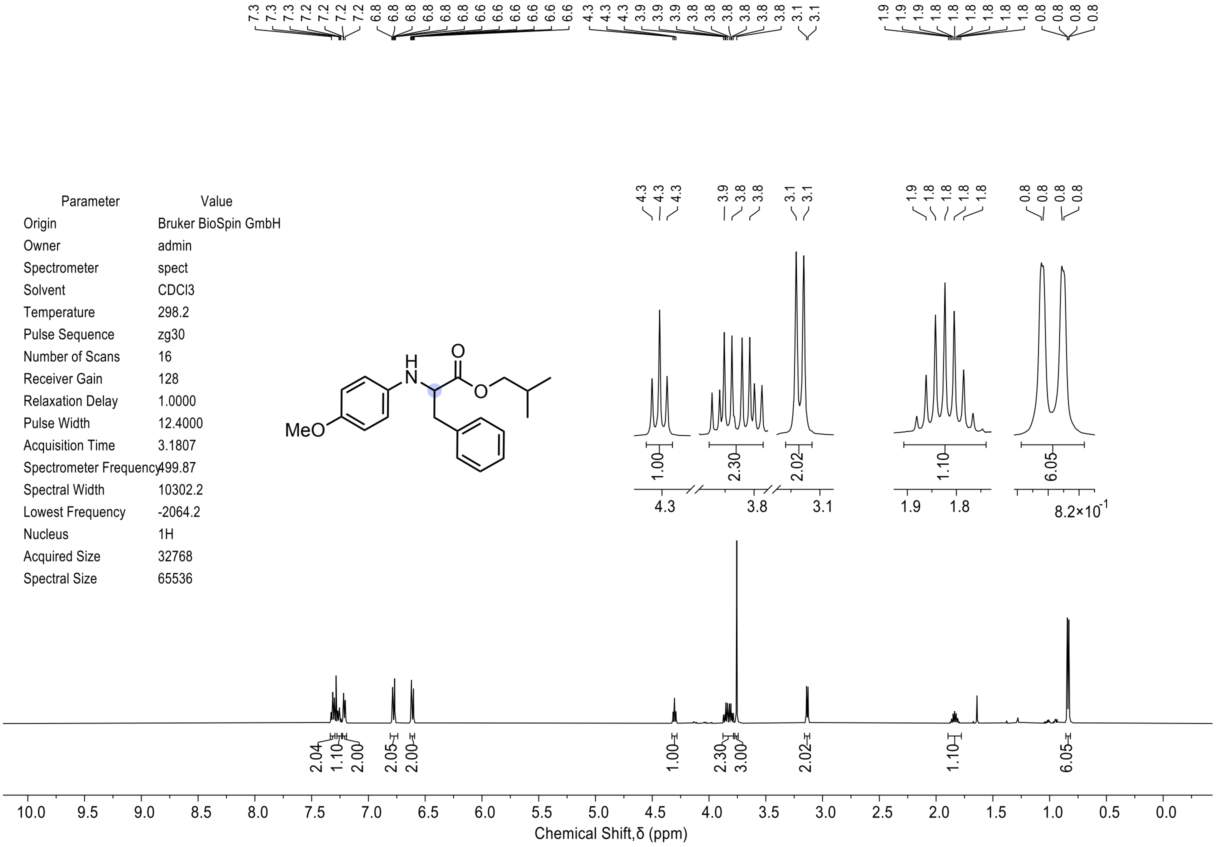

Figure S18. ^{1}H NMR (500 MHz, $CDCl_3$) spectrum of compound **3ea.**

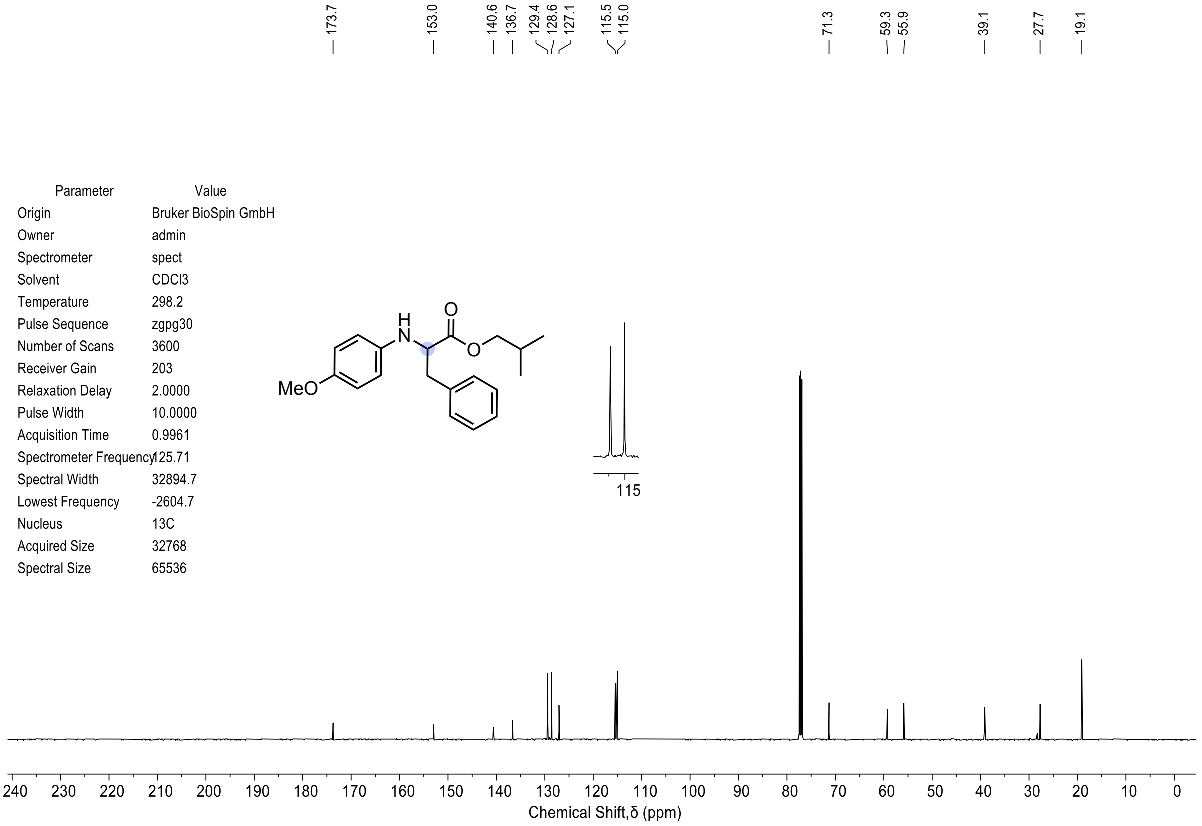

Figure S19. $^{13}C\{^{1}H\}$ NMR (126 MHz, $CDCl_3$) spectrum of compound **3ea**.

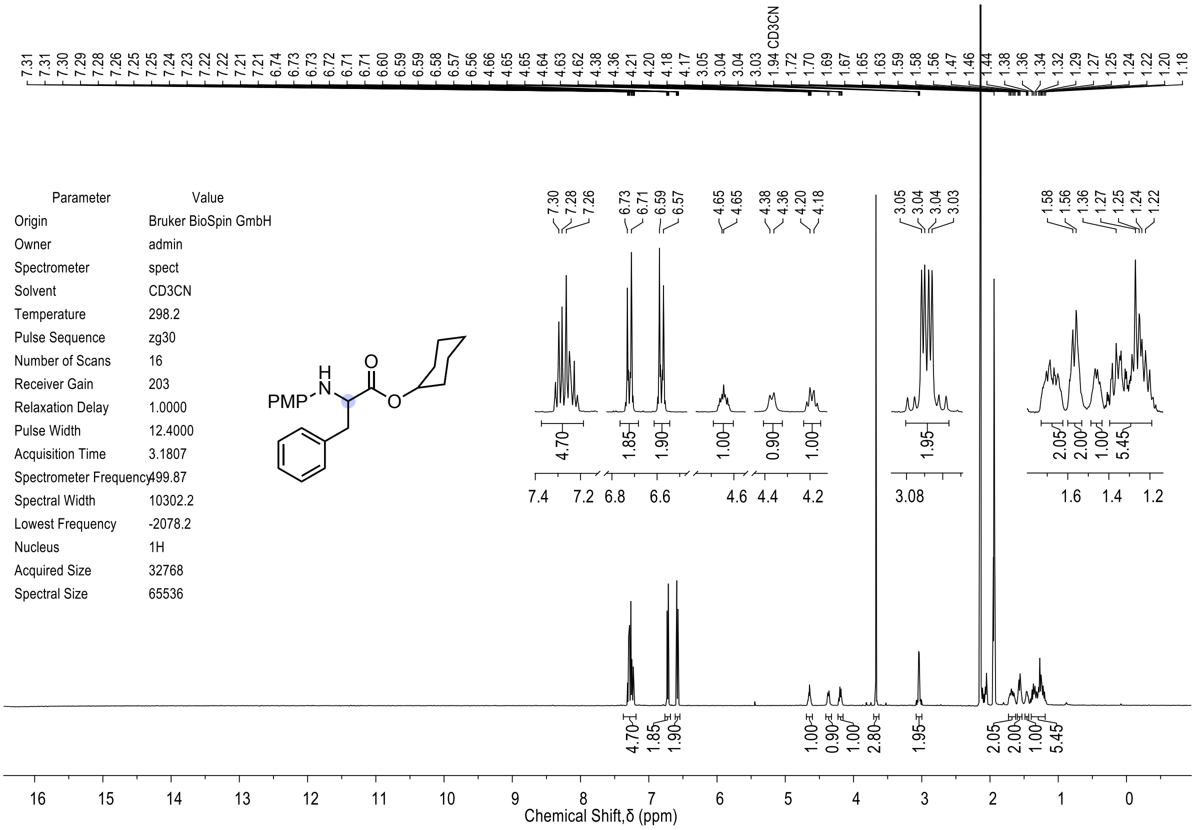

Figure S20. ^{1}H NMR (500 MHz, CD_3CN) spectrum of compound **3fa**.

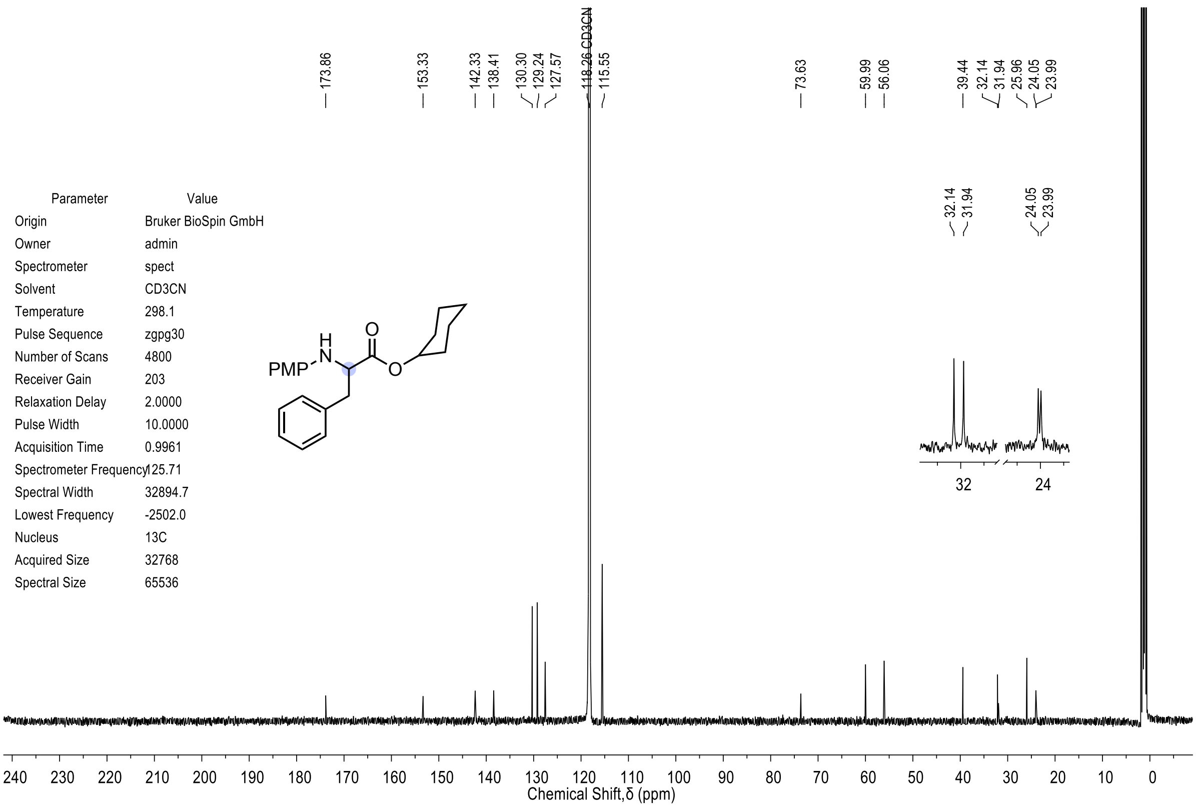

Figure S21. $^{13}C\{^{1}H\}$ NMR (126 MHz, CD_3CN) spectrum of compound **3fa**.

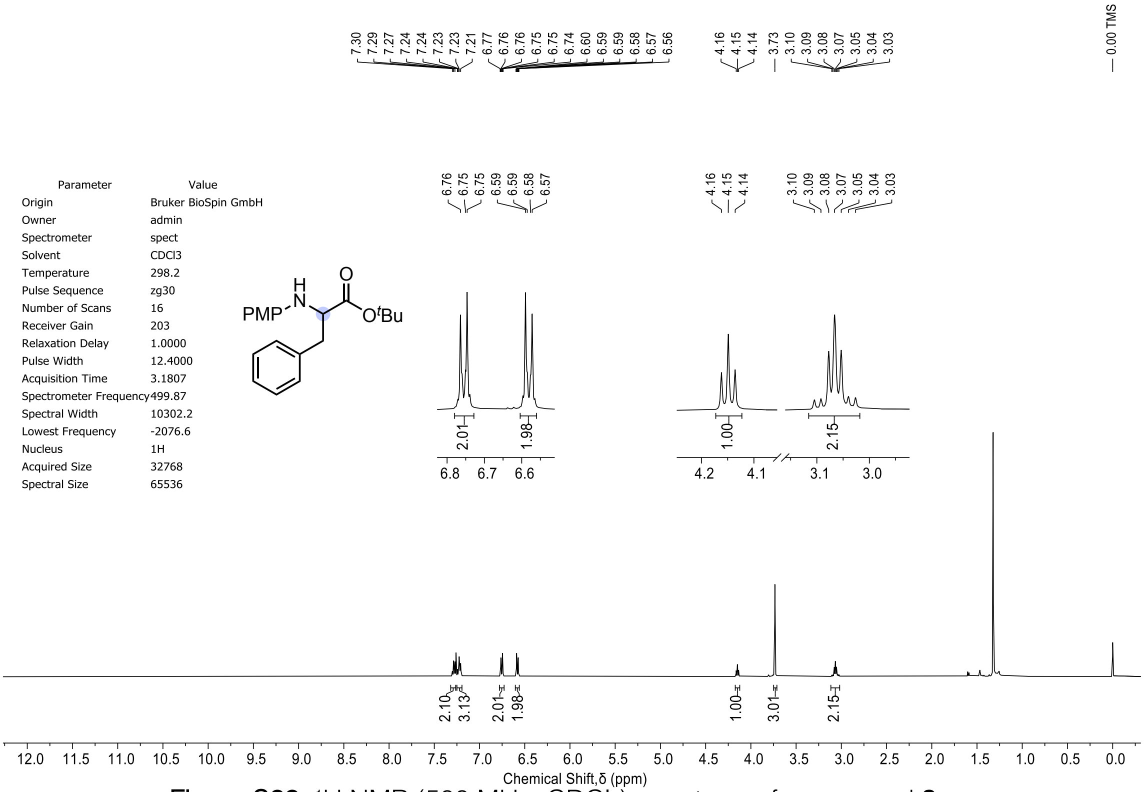

Figure S22. ^{1}H NMR (500 MHz, $CDCl_3$) spectrum of compound **3ga**.

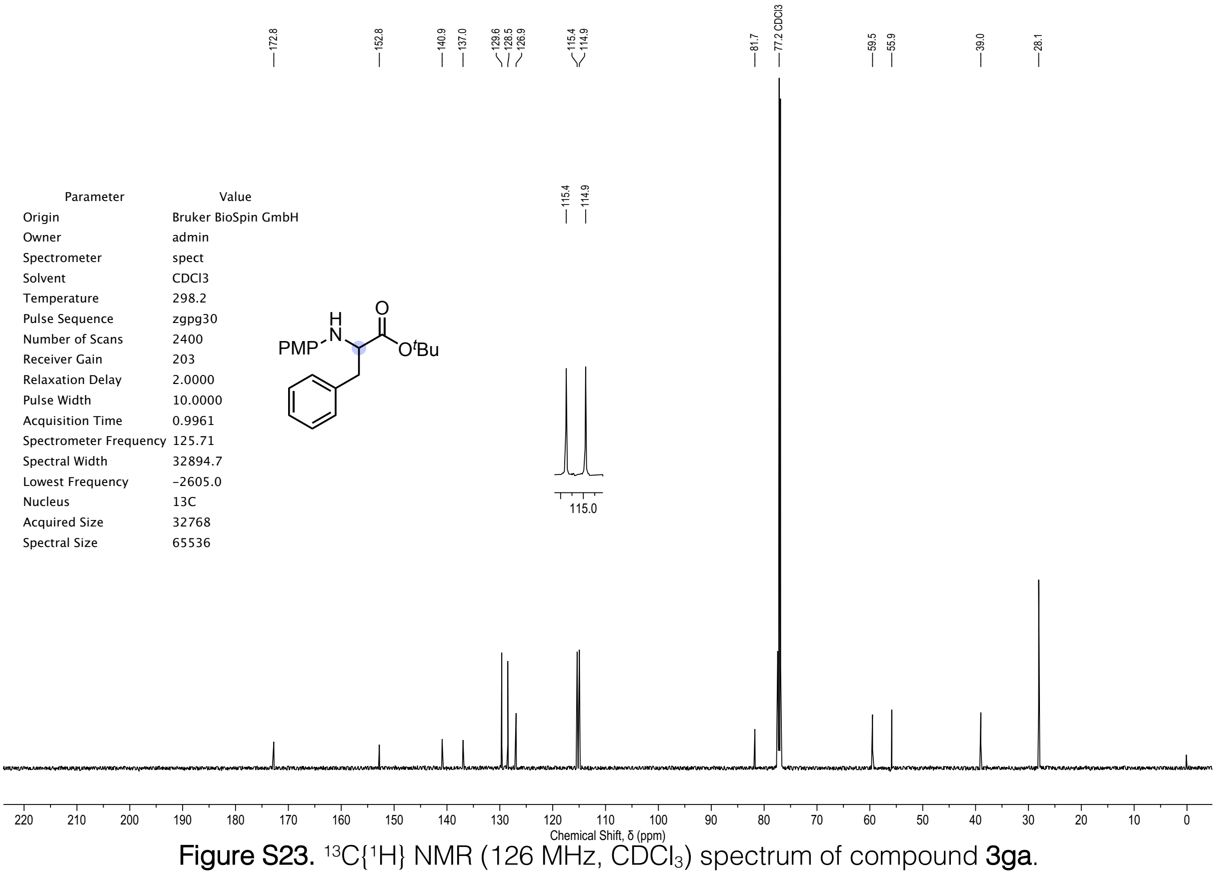

Figure S23. $^{13}C\{^{1}H\}$ NMR (126 MHz, $CDCl_3$) spectrum of compound **3ga**.

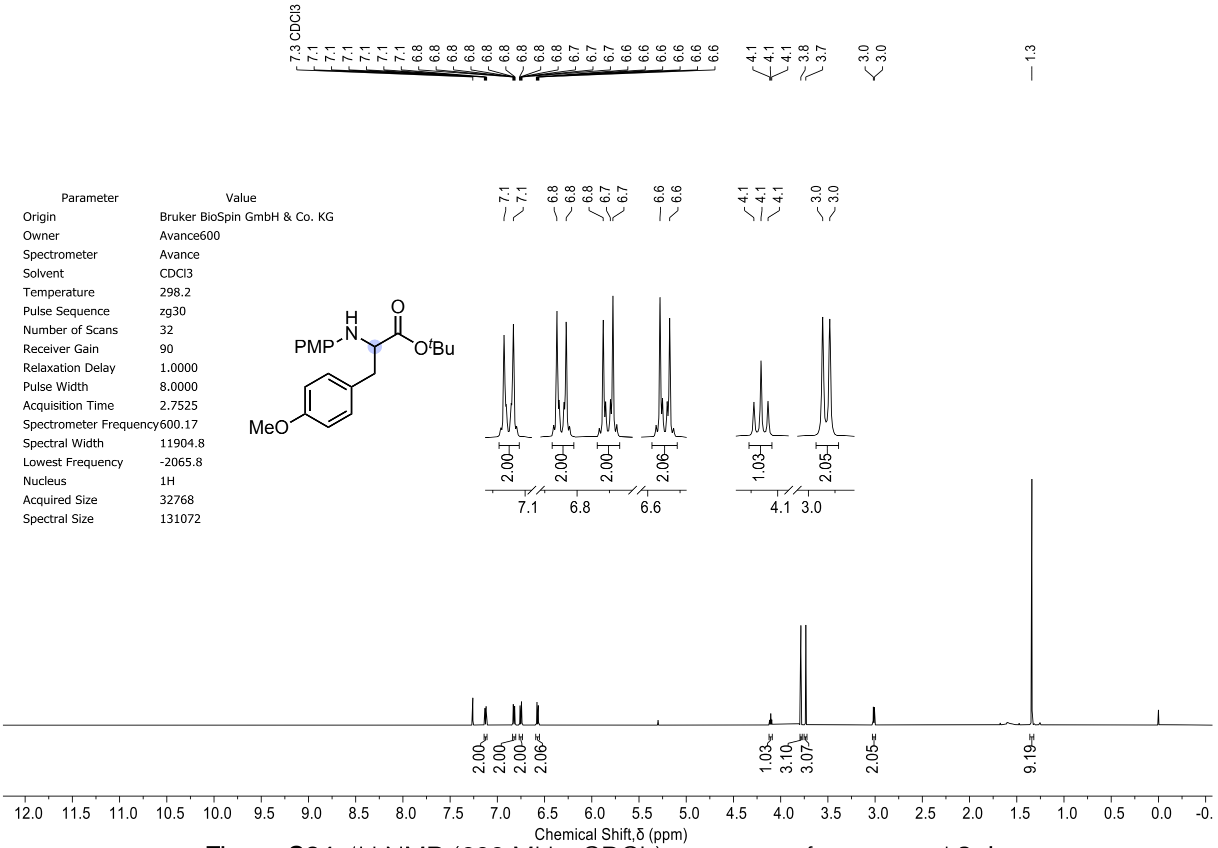

Figure S24. ^{1}H NMR (600 MHz, $CDCl_3$) spectrum of compound **3gb**.

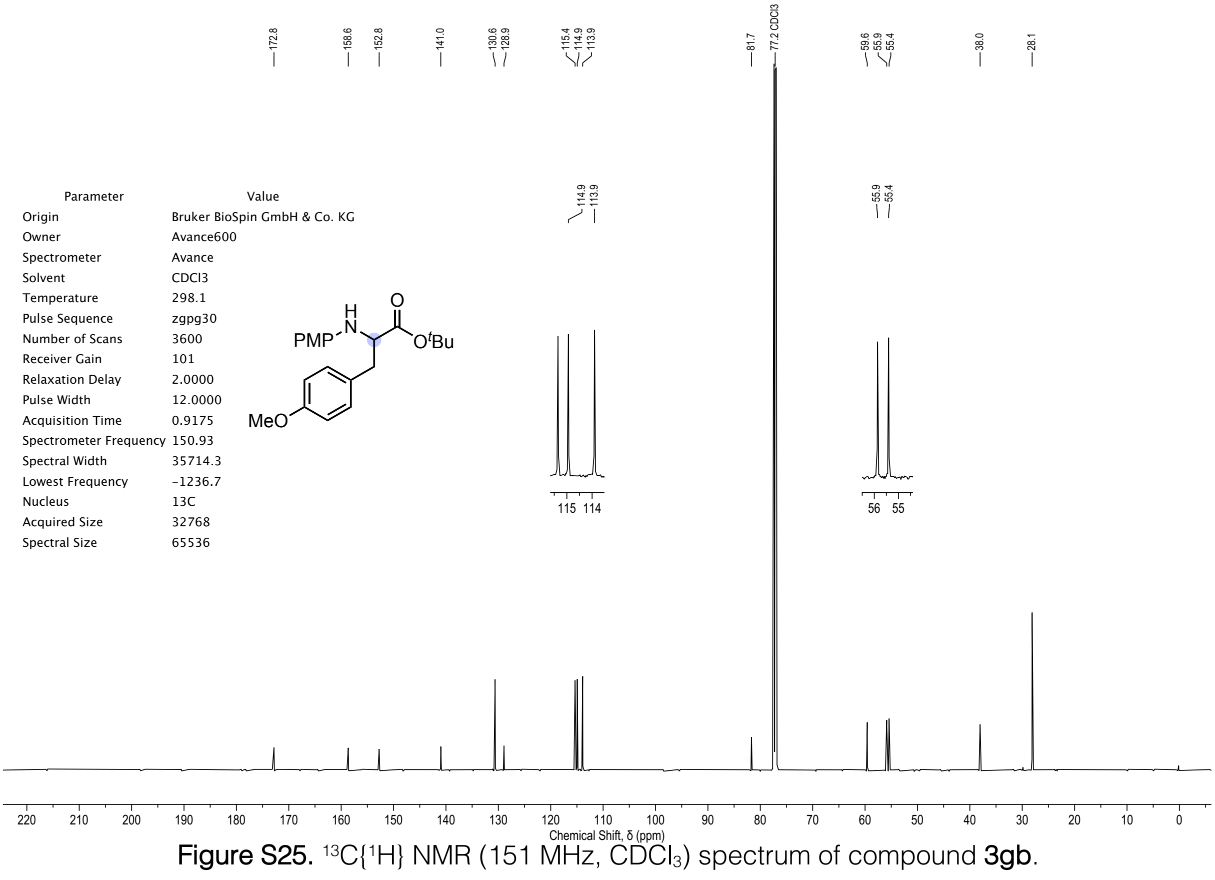

Figure S25. $^{13}C\{^{1}H\}$ NMR (151 MHz, $CDCl_3$) spectrum of compound **3gb**.

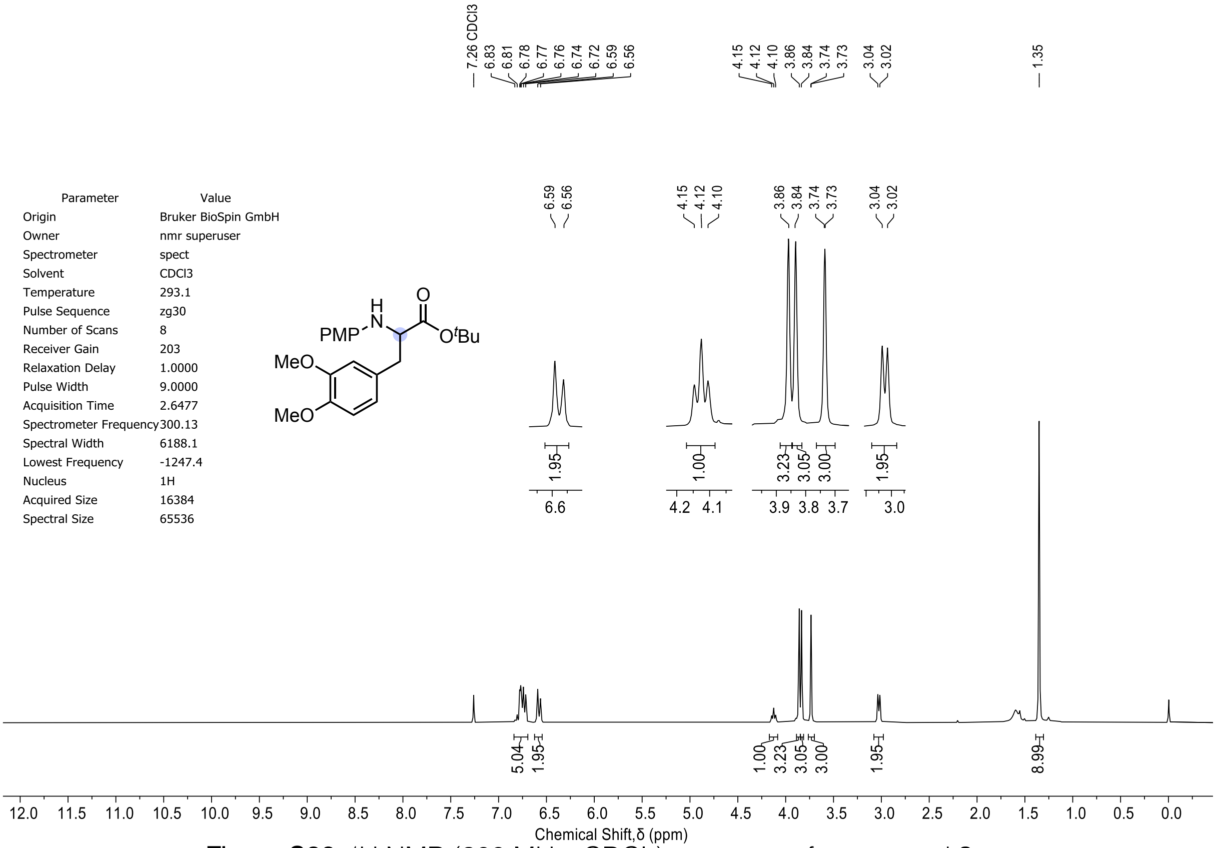

Figure S26. ^{1}H NMR (300 MHz, $CDCl_3$) spectrum of compound **3gc**.

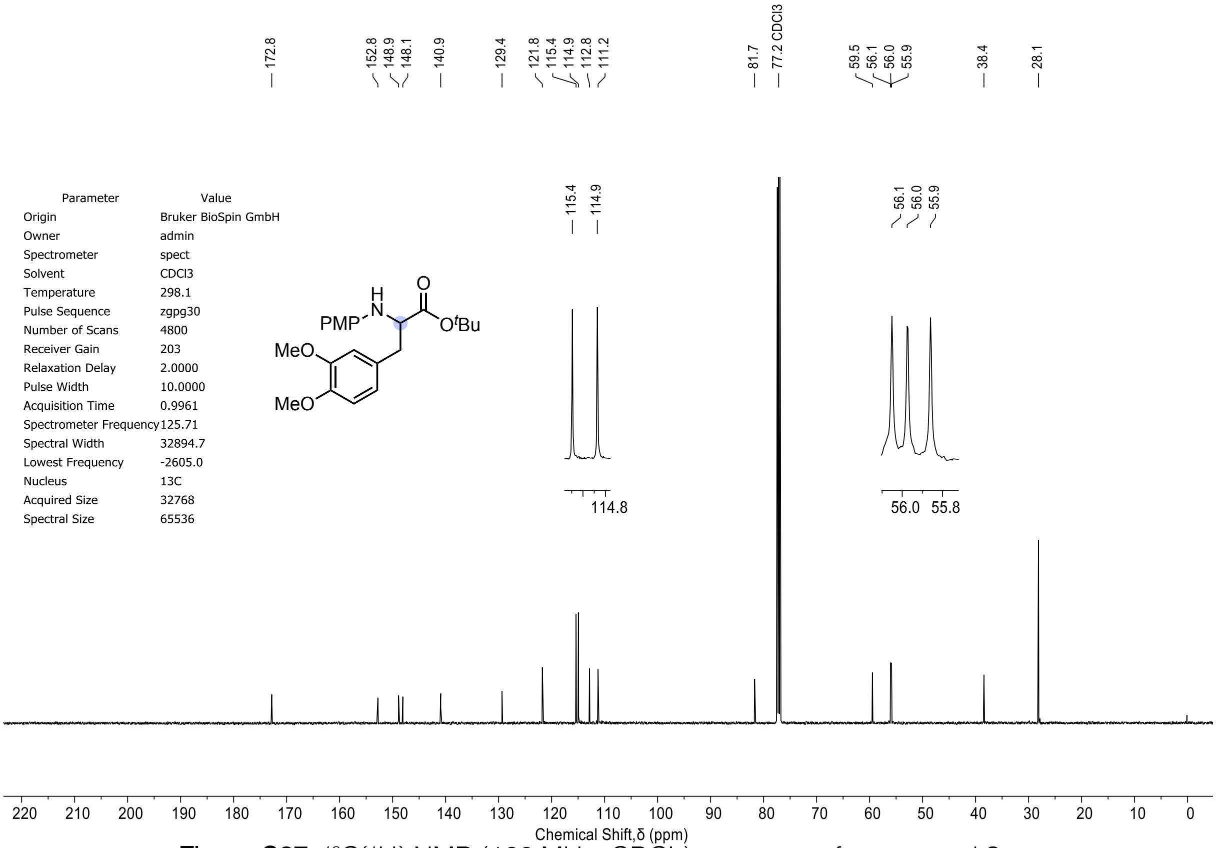

Figure S27. $^{13}C\{^{1}H\}$ NMR (126 MHz, $CDCl_3$) spectrum of compound **3gc**.

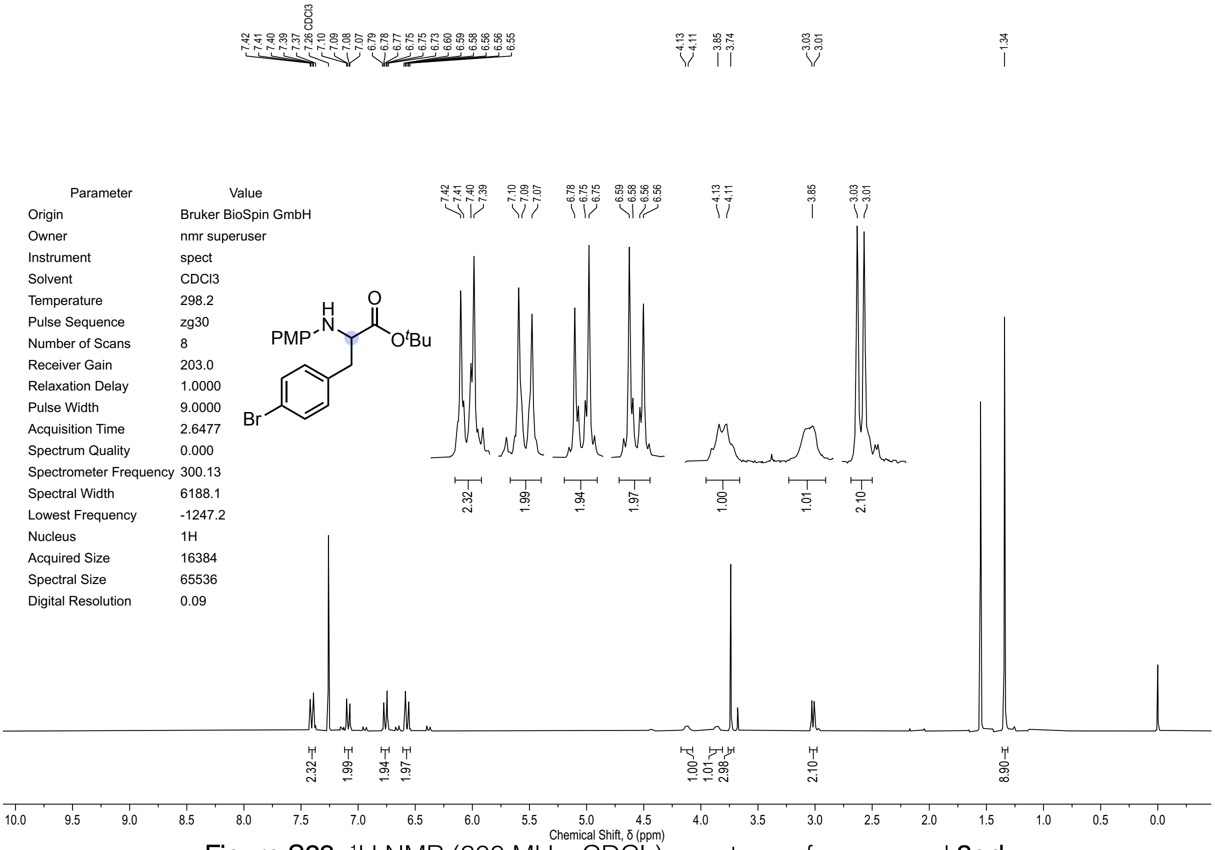

Figure S28. ^{1}H NMR (300 MHz, $CDCl_3$) spectrum of compound **3gd**.

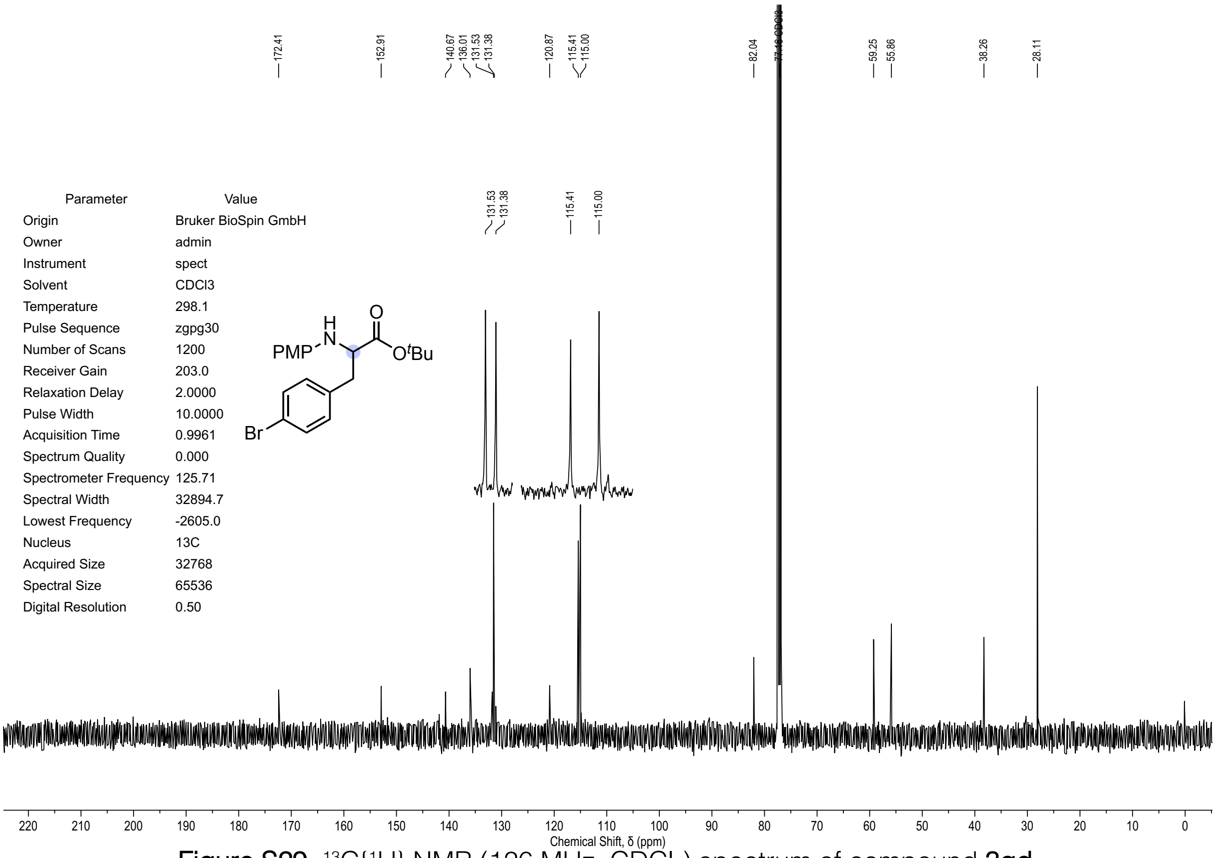

Figure S29. $^{13}C\{^{1}H\}$ NMR (126 MHz, $CDCl_3$) spectrum of compound **3gd**.

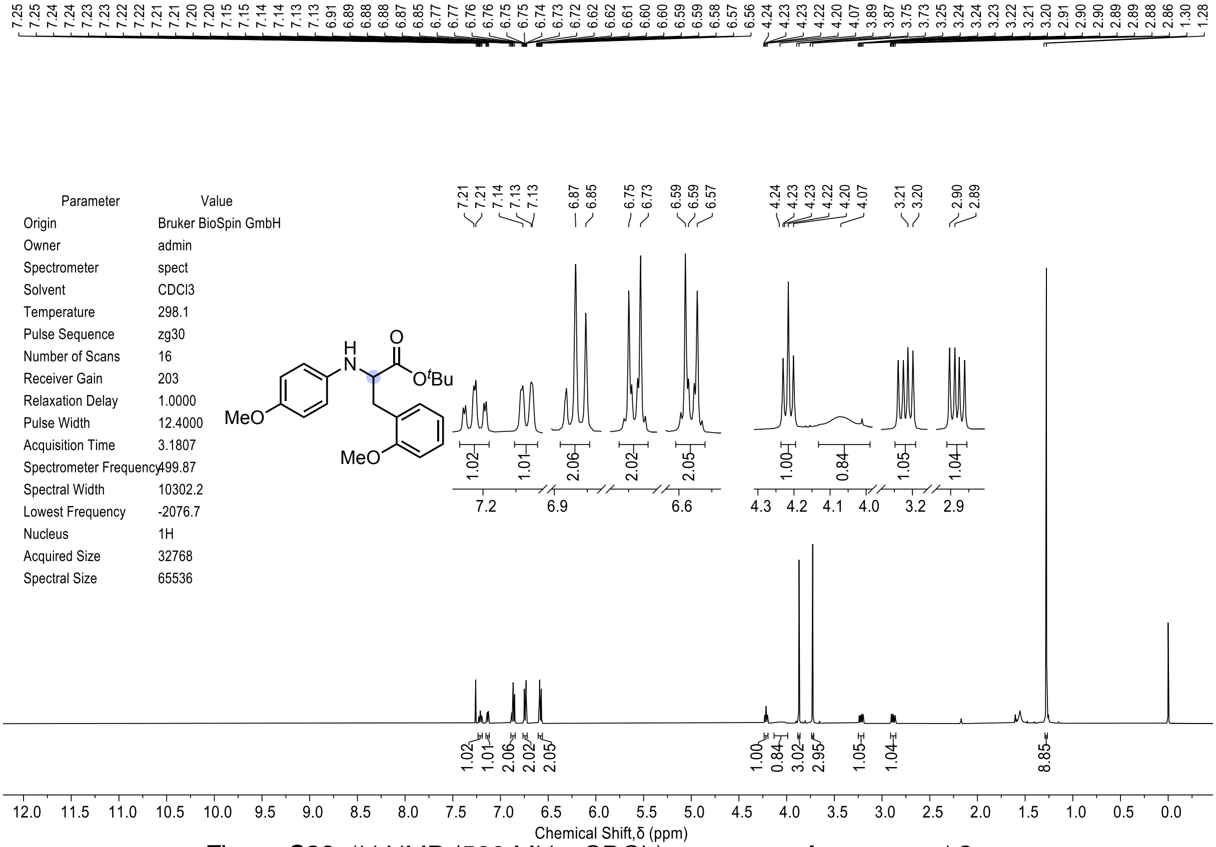

Figure S30. ^{1}H NMR (500 MHz, $CDCl_3$) spectrum of compound **3ge**.

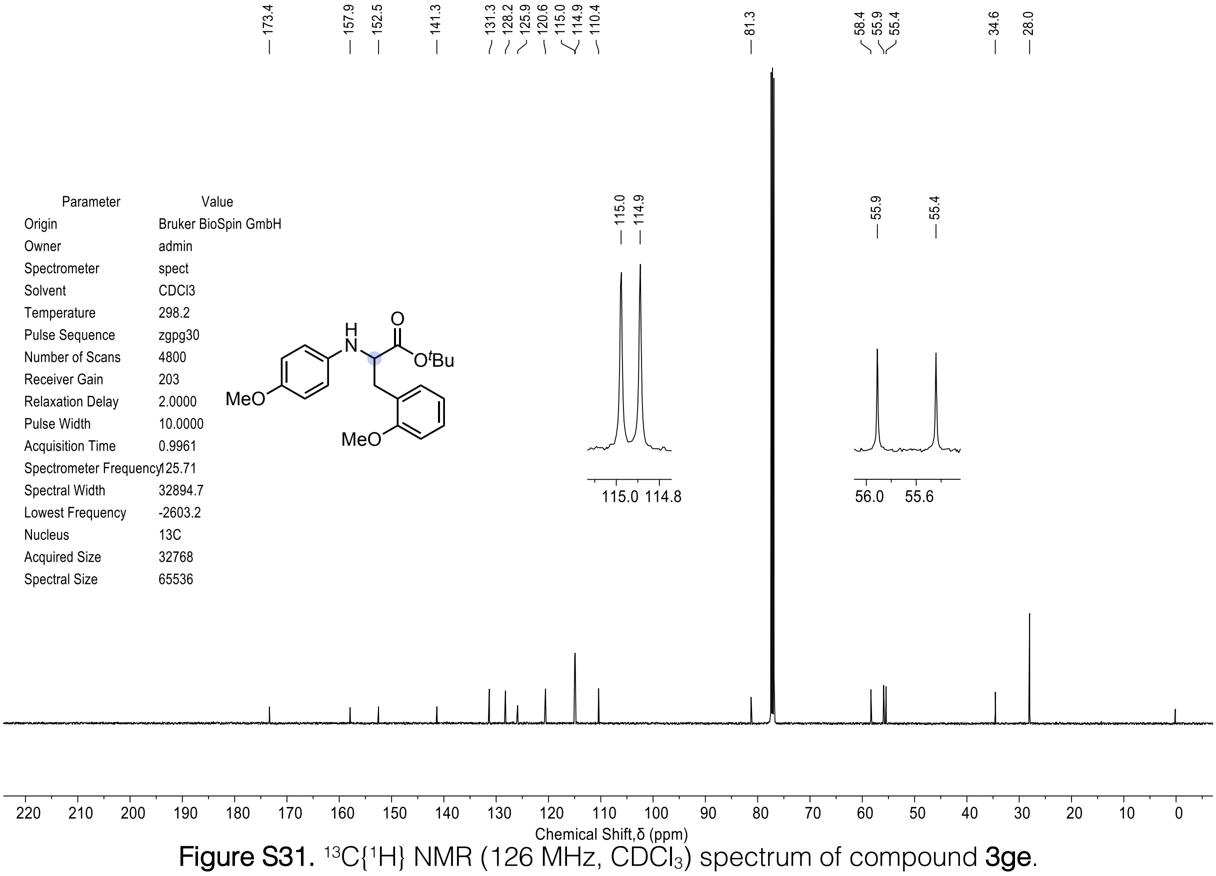

Figure S31. $^{13}C\{^{1}H\}$ NMR (126 MHz, $CDCl_3$) spectrum of compound **3ge**.

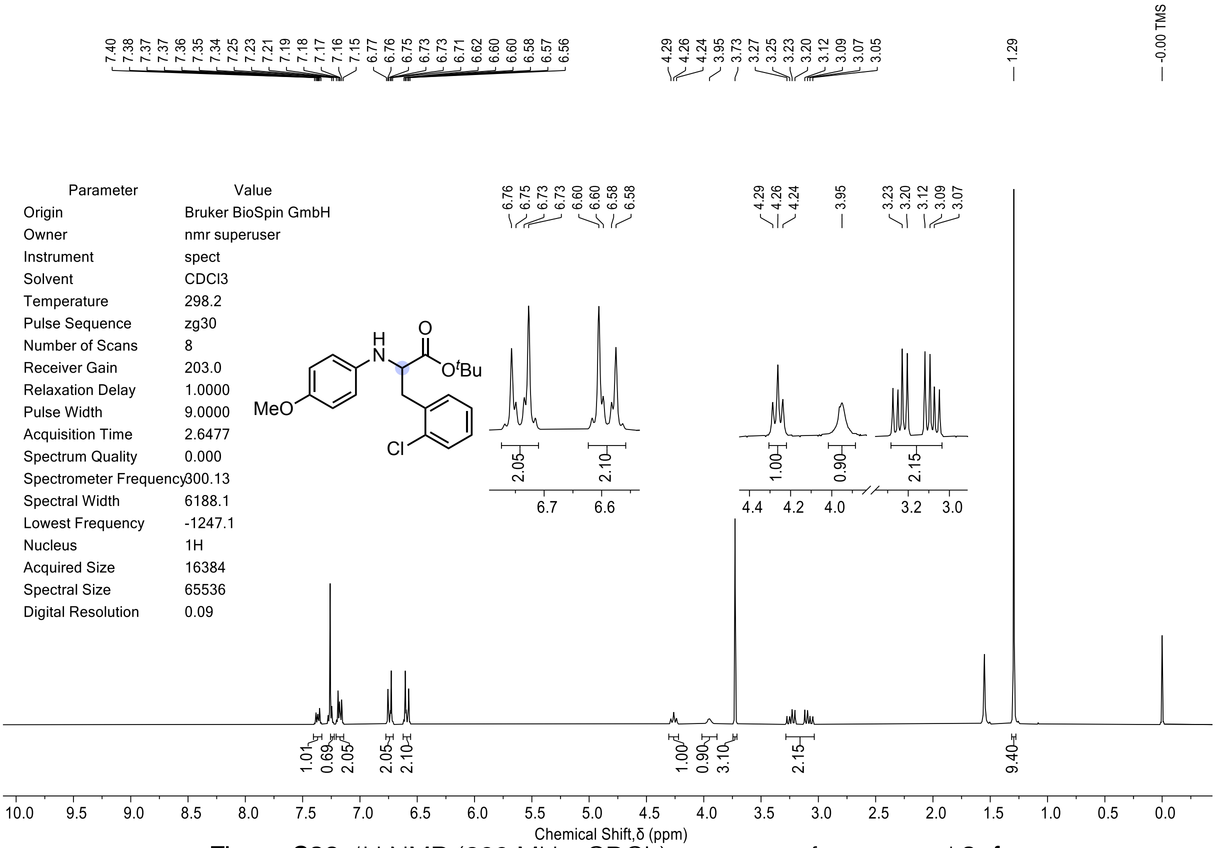

Figure S32. ^{1}H NMR (300 MHz, $CDCl_3$) spectrum of compound **3gf**.

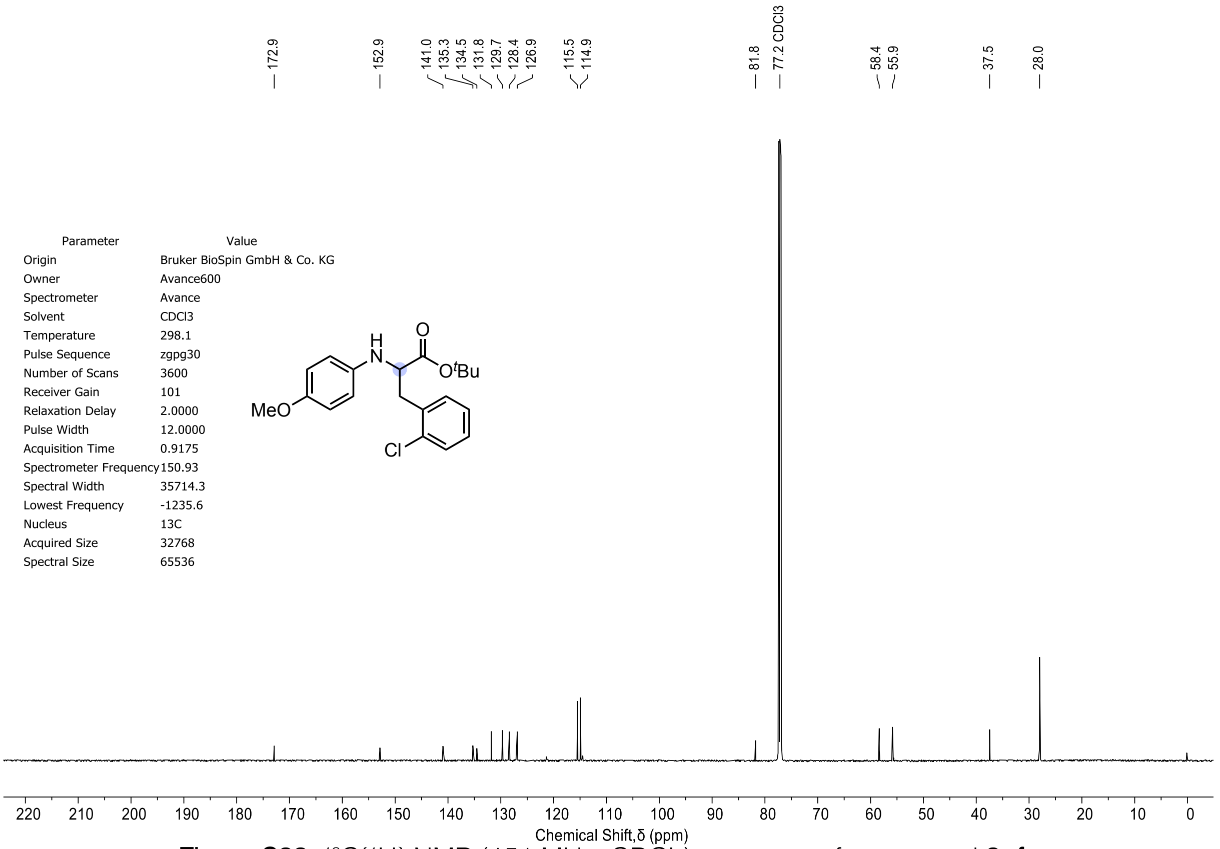

Figure S33. $^{13}C\{^{1}H\}$ NMR (151 MHz, $CDCl_3$) spectrum of compound **3gf**.

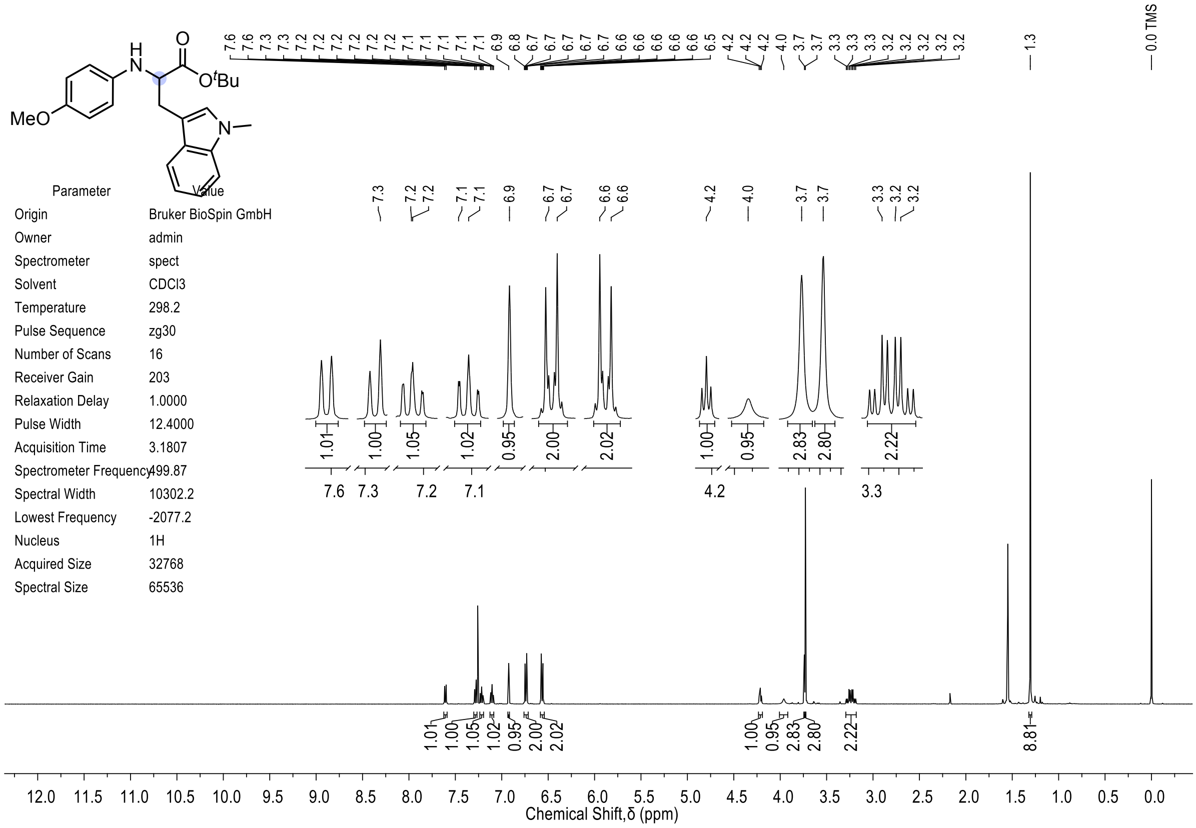

Figure S34. ^{1}H NMR (500 MHz, $CDCl_3$) spectrum of compound **3gg**.

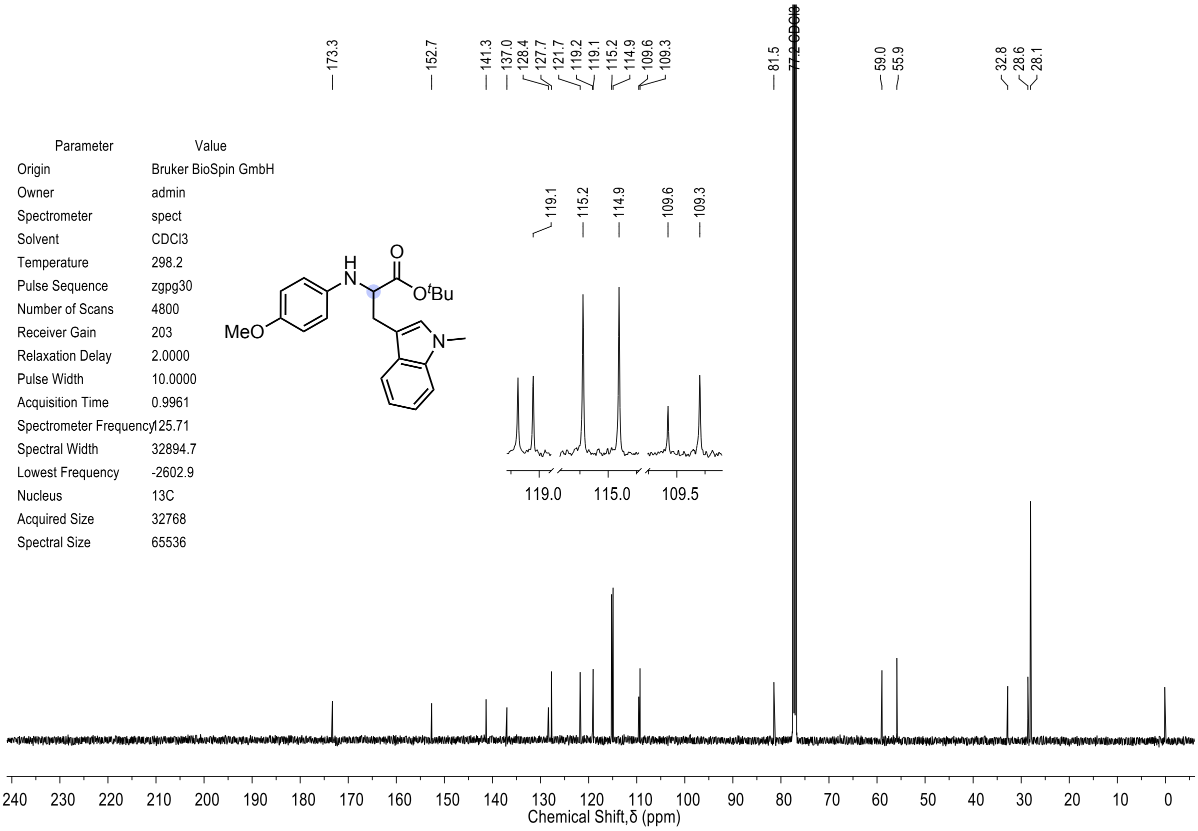

Figure S35. $^{13}C\{^{1}H\}$ NMR (126 MHz, $CDCl_3$) spectrum of compound **3gg**.

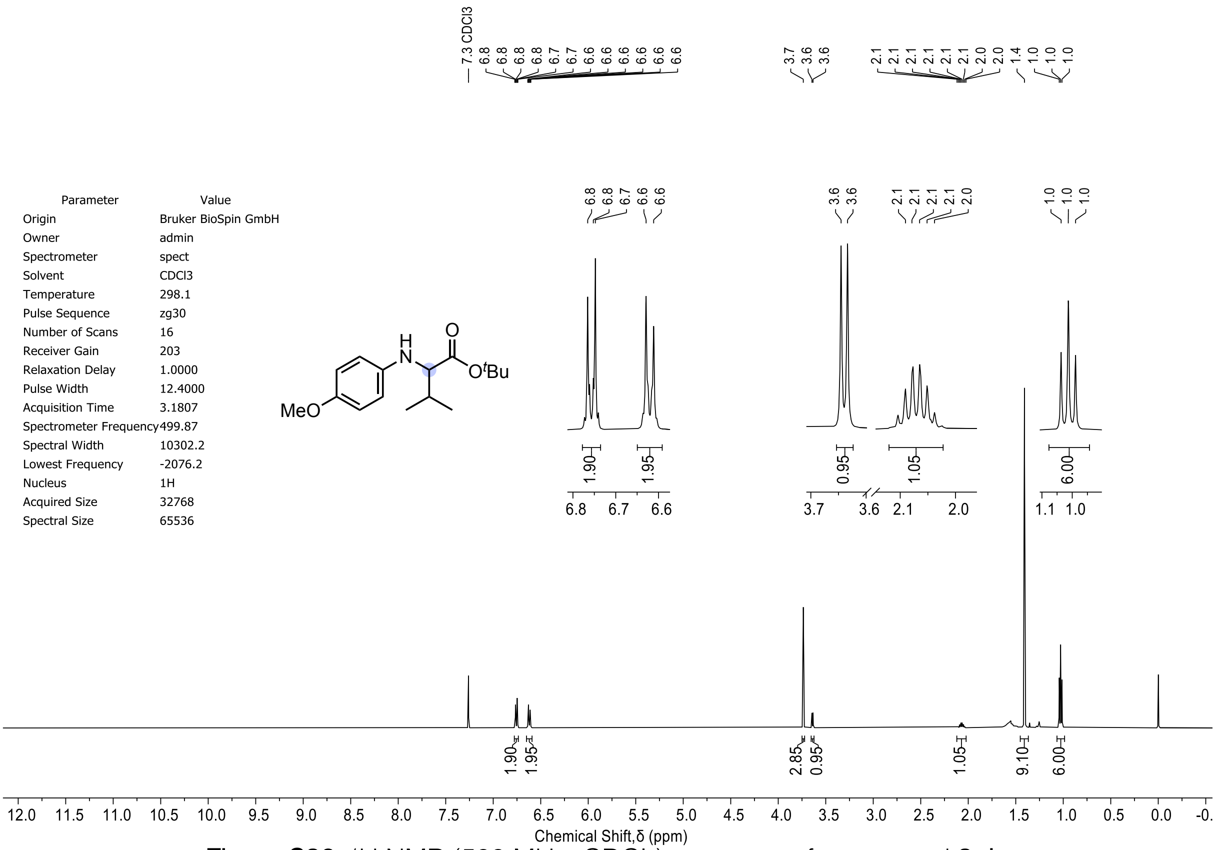

Figure S36. ^{1}H NMR (500 MHz, $CDCl_3$) spectrum of compound **3gh**.

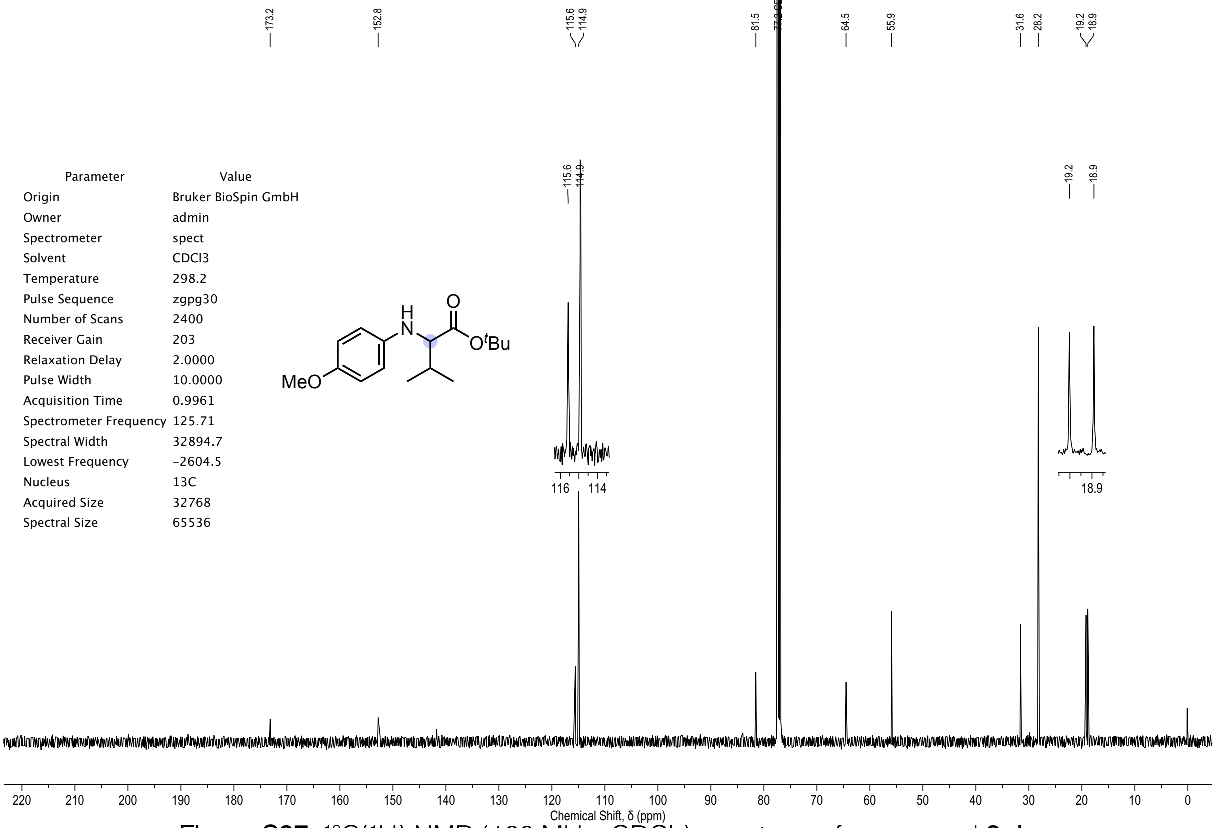

Figure S37. $^{13}C\{^1H\}$ NMR (126 MHz, $CDCl_3$) spectrum of compound **3gh**.

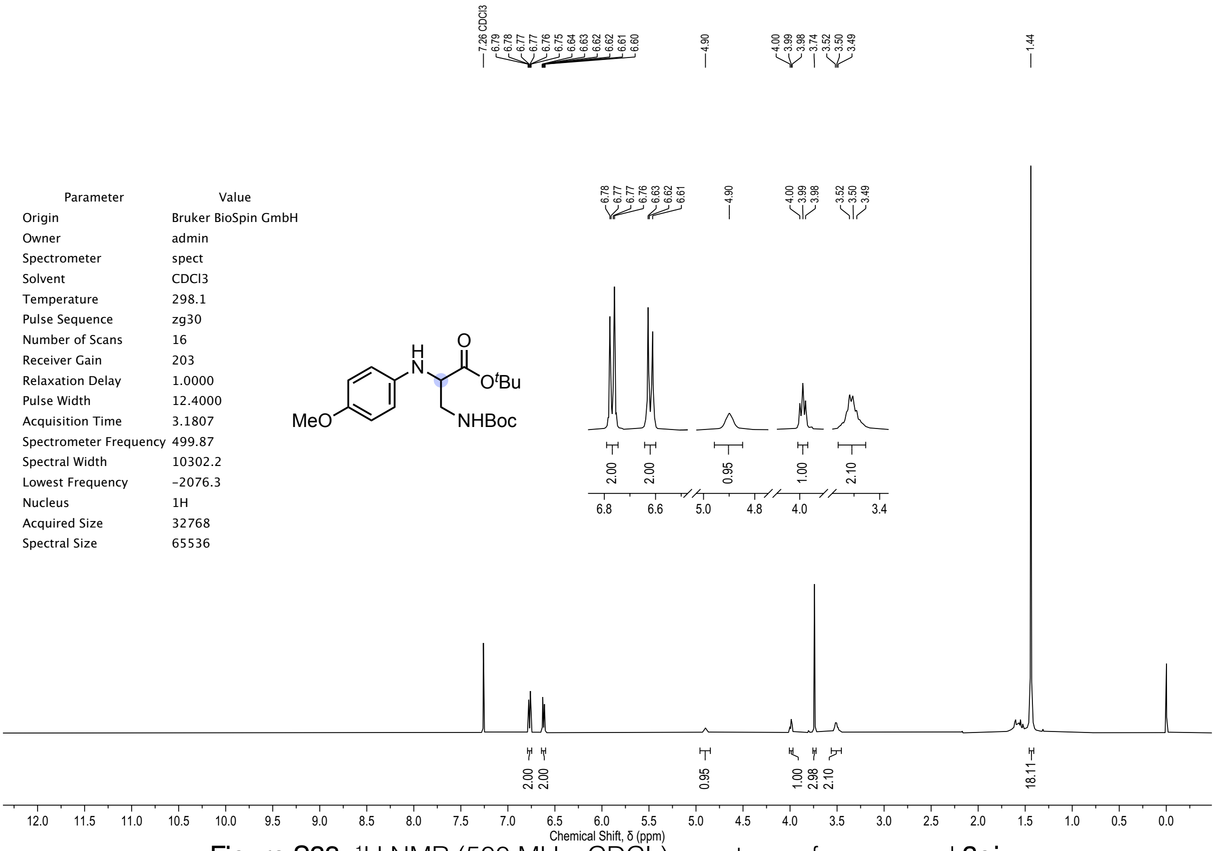

Figure S38. ^{1}H NMR (500 MHz, $CDCl_3$) spectrum of compound **3gi**.

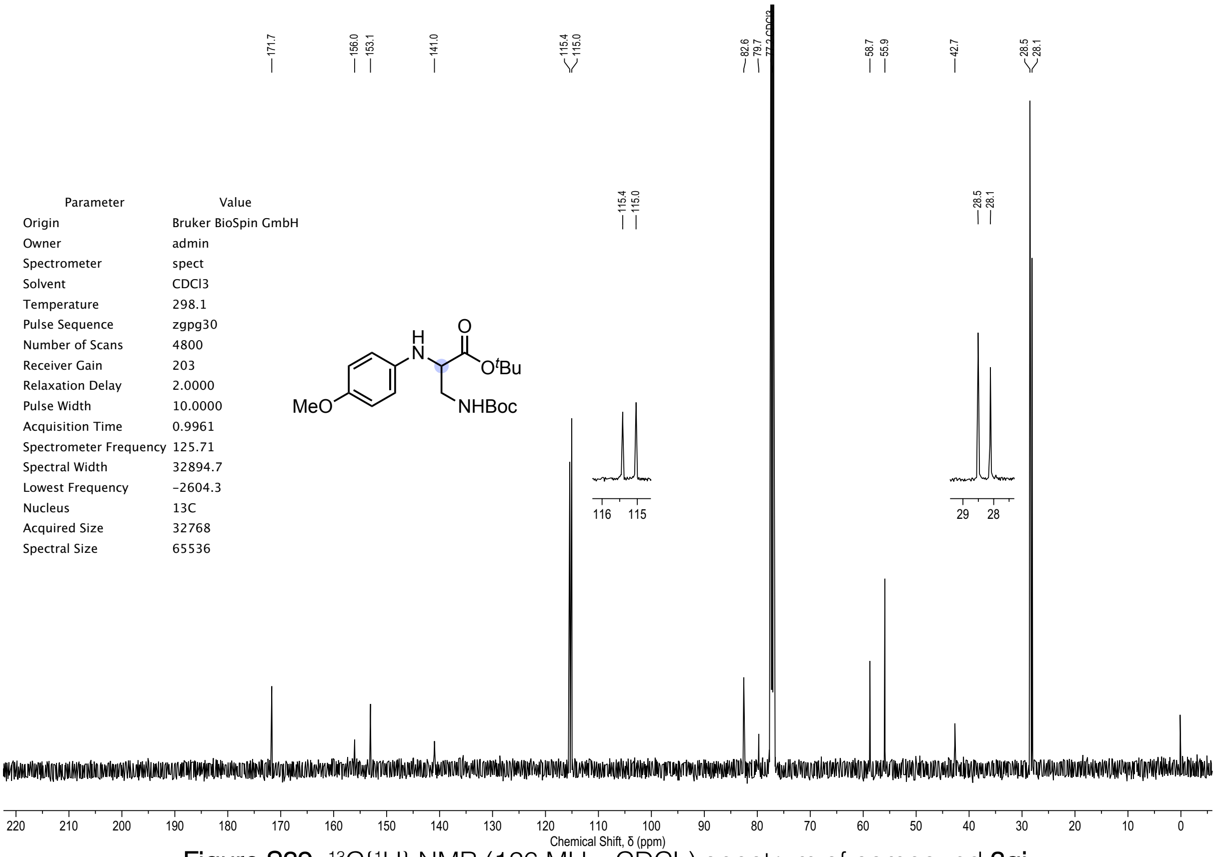

Figure S39. $^{13}C\{^1H\}$ NMR (126 MHz, $CDCl_3$) spectrum of compound **3gi**.

XII. CHROMATOGRAMS

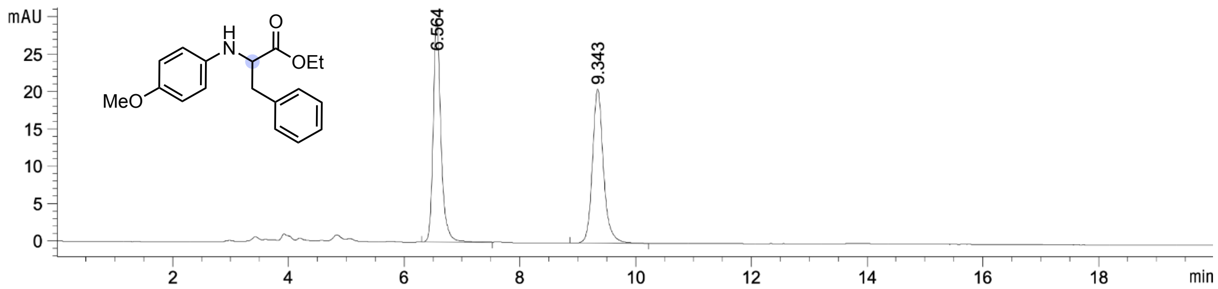

Signal 6: DAD1 F, Sig=260,4 Ref=off

Peak #	RetTime [min]	Type	Width [min]	Area [mAU*s]	Height [mAU]	Area %
1	6.564	BB	0.1382	277.60077	29.94056	50.4227
2	9.343	BB	0.2002	272.94617	20.64960	49.5773

Figure S40. HPLC trace of *rac*-**3aa**.

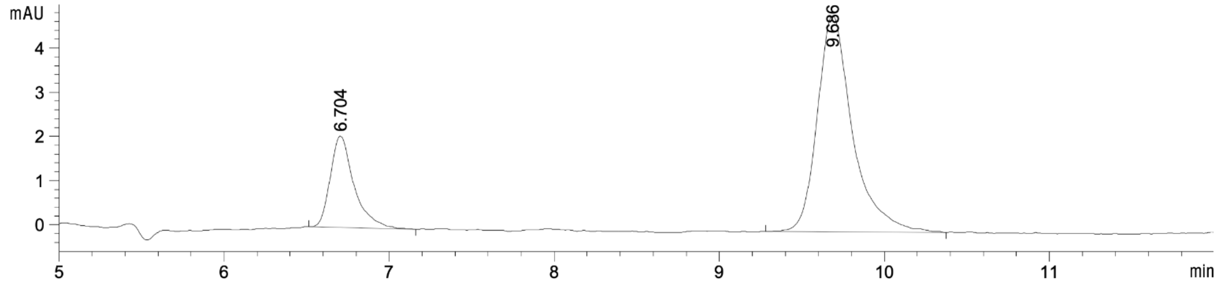

Signal 5: DAD1 E, Sig=260,4 Ref=off

Peak #	RetTime [min]	Type	Width [min]	Area [mAU*s]	Height [mAU]	Area %
1	6.704	BB	0.1491	20.76073	2.06776	22.2299
2	9.686	BB	0.2231	72.63041	4.89439	77.7701

Figure S41. HPLC trace of (*S*)-**3aa**.

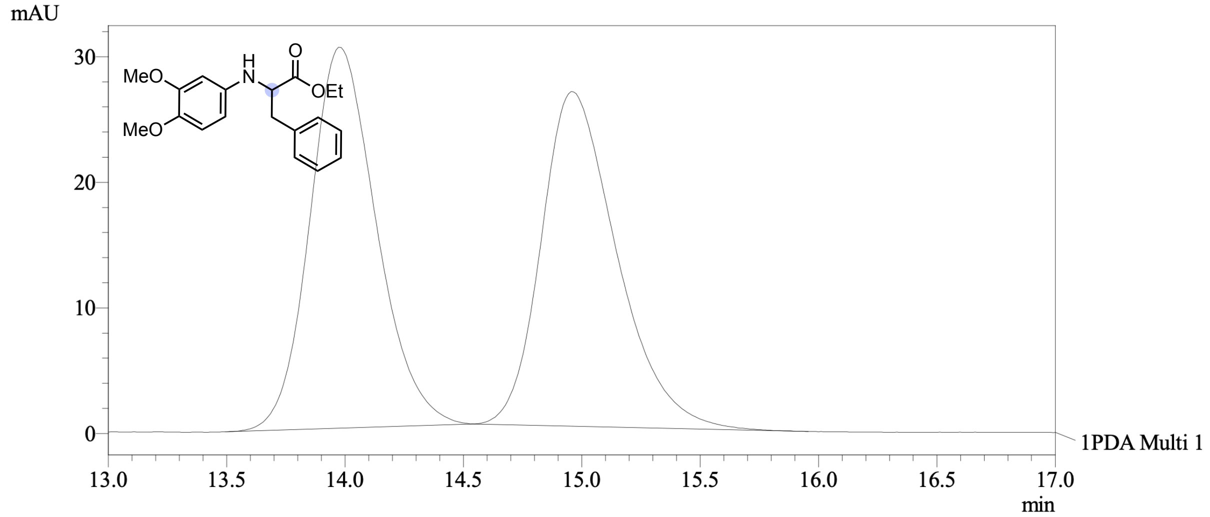

1 PDA Multi 1 / 300nm 4nm

PeakTable

PDA Ch1 300nm 4nm

Peak#	Ret. Time	Area	Height	Area %	Height %
1	13.973	593771	30338	50.053	53.254
2	14.954	592510	26631	49.947	46.746
Total		1186282	56969	100.000	100.000

Figure S42. HPLC trace of *rac*-**3ba**.

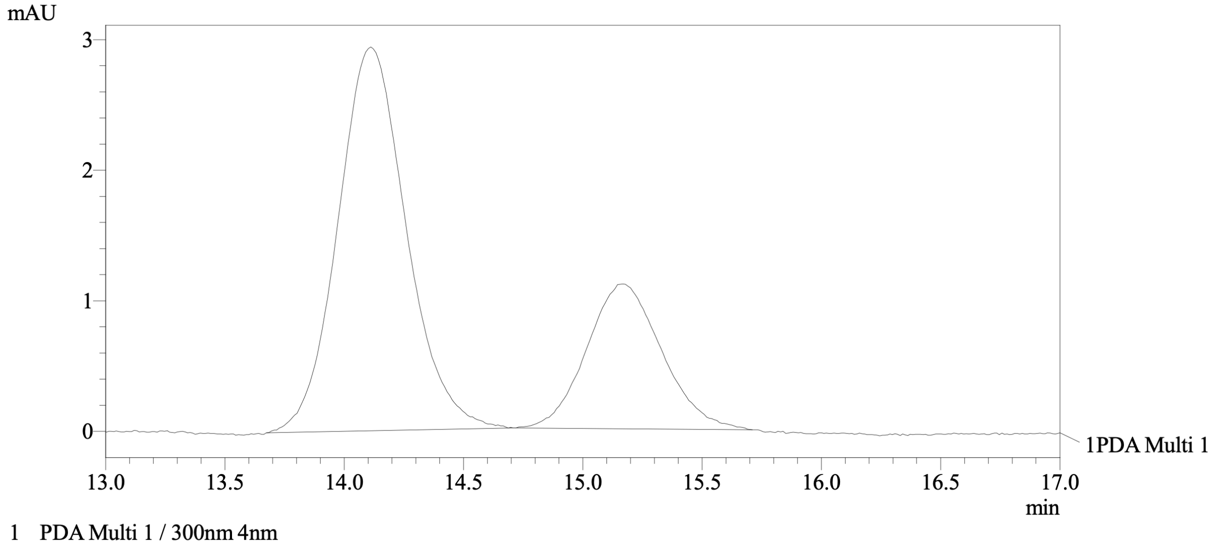

1 PDA Multi 1 / 300nm 4nm

PeakTable

PDA Ch1 300nm 4nm

Peak#	Ret. Time	Area	Height	Area %	Height %
1	14.106	58638	2940	70.677	72.575
2	15.164	24329	1111	29.323	27.425
Total		82967	4051	100.000	100.000

Figure S43. HPLC trace of (*S*)-**3ba**.

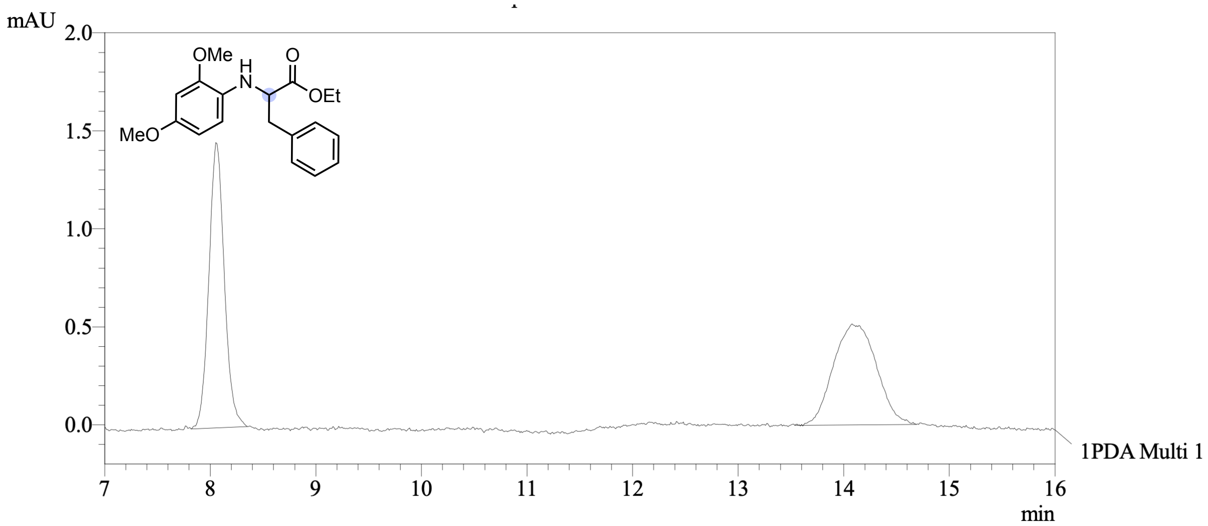

1 PDA Multi 1 / 300nm 4nm

PeakTable

PDA Ch1 300nm 4nm

Peak#	Ret. Time	Area	Height	Area %	Height %
1	8.052	14782	1456	50.341	73.835
2	14.073	14581	516	49.659	26.165
Total		29363	1972	100.000	100.000

Figure S44. HPLC trace of *rac*-**3ca**.

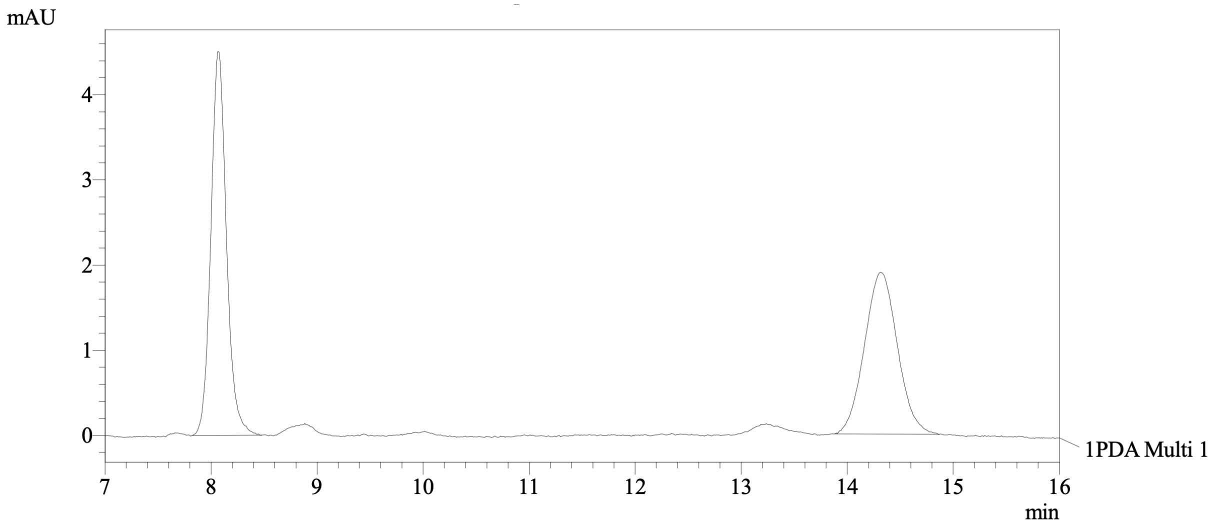

1 PDA Multi 1 / 300nm 4nm

PeakTable

PDA Ch1 300nm 4nm

Peak#	Ret. Time	Area	Height	Area %	Height %
1	8.064	46083	4508	52.922	70.345
2	14.311	40994	1900	47.078	29.655
Total		87077	6408	100.000	100.000

Figure S45. HPLC trace of **3ca**.

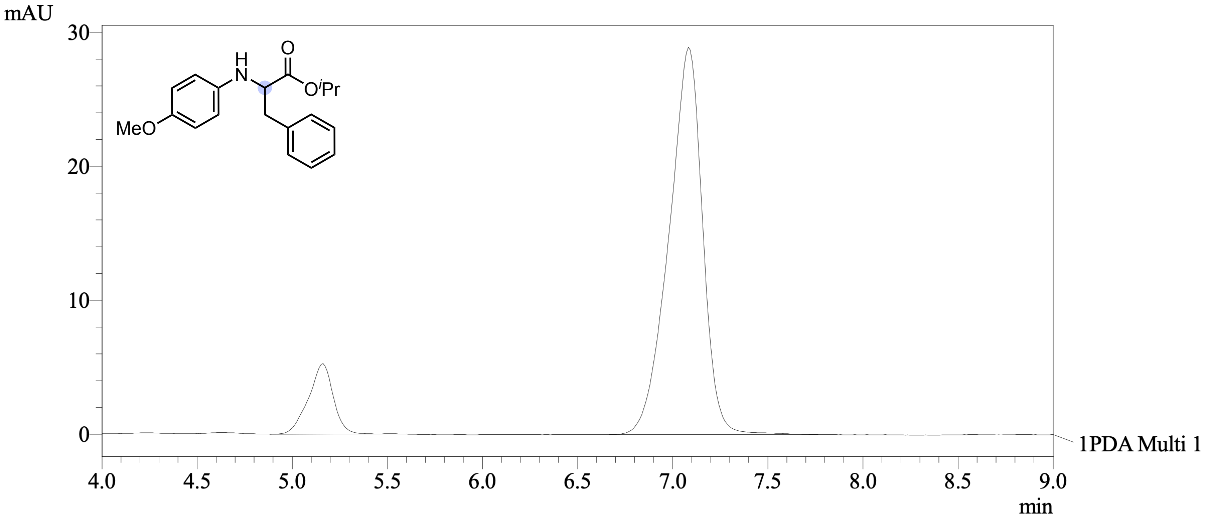

1 PDA Multi 1 / 310nm 4nm

PeakTable

PDA Ch1 310nm 4nm

Peak#	Ret. Time	Area	Height	Area %	Height %
1	5.154	47306	5259	11.721	15.380
2	7.079	356299	28933	88.279	84.620
Total		403605	34192	100.000	100.000

Figure S46. HPLC trace of (*S*)-**3da**.

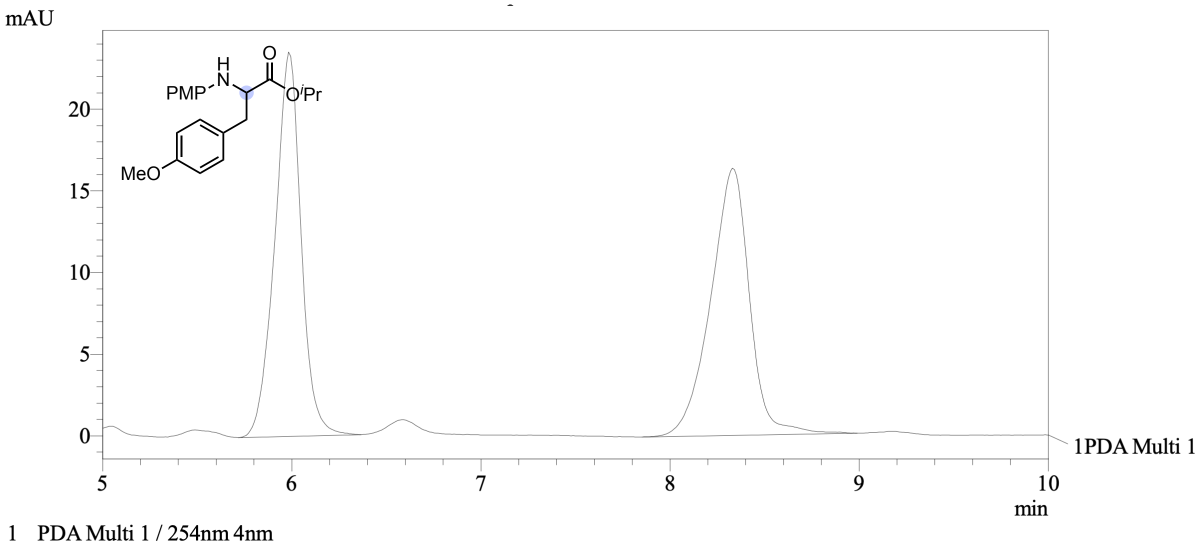

PeakTable

PDA Ch1 254nm 4nm

Peak#	Ret. Time	Area	Height	Area %	Height %
1	5.981	226168	23539	49.161	58.987
2	8.327	233884	16366	50.839	41.013
Total		460052	39905	100.000	100.000

Figure S47. HPLC trace of *rac*-3db.

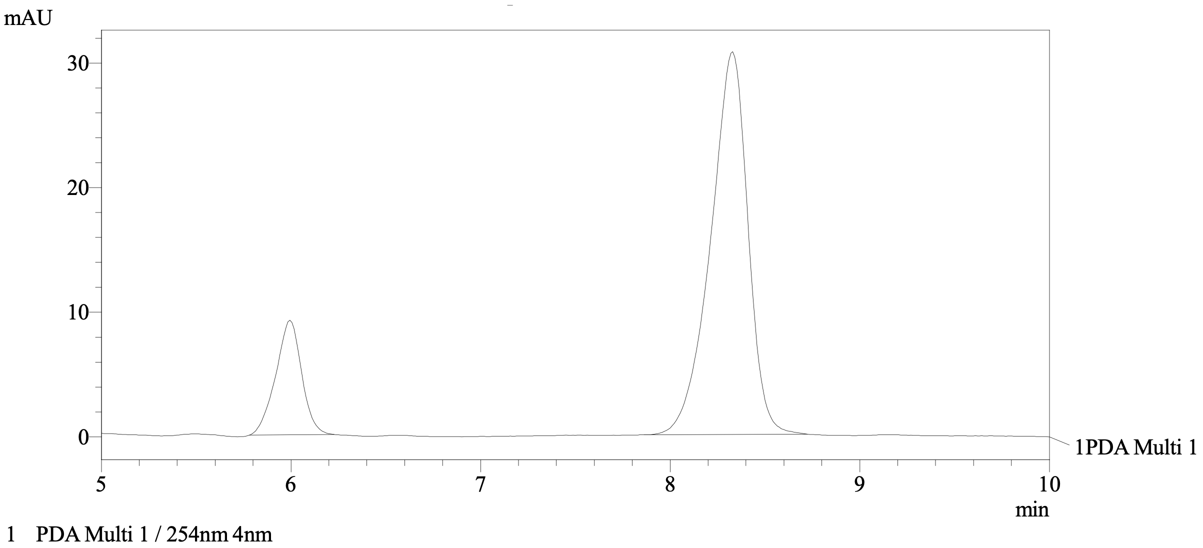

PeakTable

PDA Ch1 254nm 4nm

Peak#	Ret. Time	Area	Height	Area %	Height %
1	5.989	87306	9201	17.169	23.039
2	8.322	421215	30734	82.831	76.961
Total		508521	39934	100.000	100.000

Figure S48. HPLC trace of (*S*)-3db.

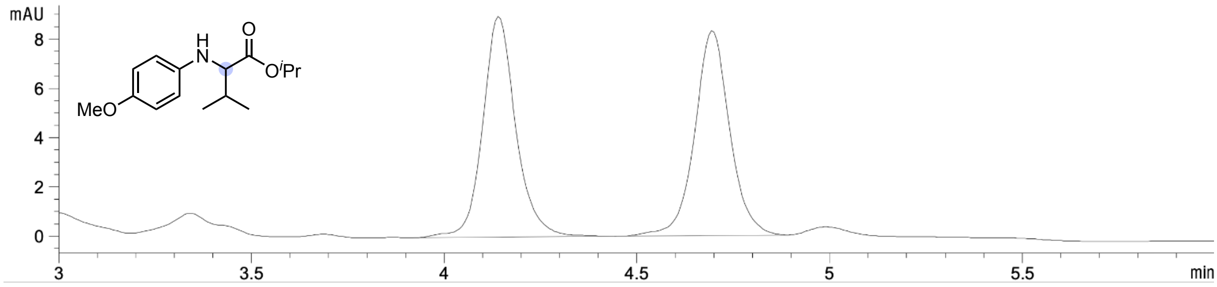

Signal 4: DAD1 D, Sig=254,4 Ref=off

Peak #	RetTime [min]	Type	Width [min]	Area [mAU*s]	Height [mAU]	Area %
1	4.141	BB	0.0919	53.78237	8.97647	50.5255
2	4.695	BB	0.0958	52.66359	8.32588	49.4745

Figure S49. HPLC trace of *rac*-**3dh**.

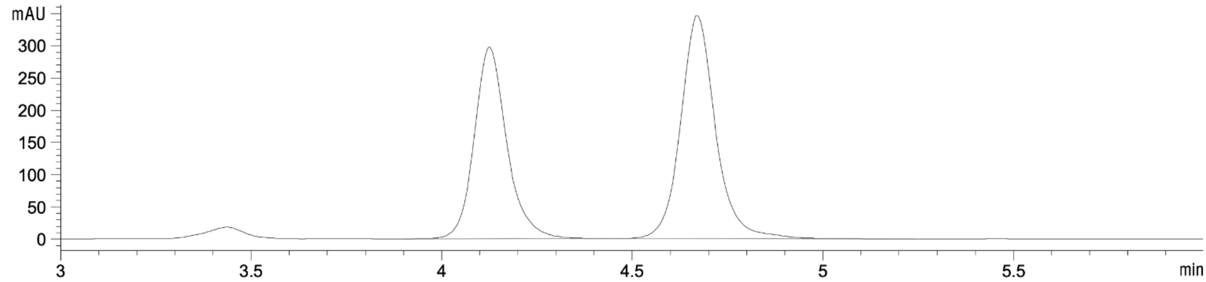

Signal 4: DAD1 D, Sig=254,4 Ref=off

Peak #	RetTime [min]	Type	Width [min]	Area [mAU*s]	Height [mAU]	Area %
1	4.125	BB	0.0892	1769.84485	298.34421	44.3993
2	4.670	BB	0.0945	2216.35474	346.93506	55.6007

Figure S50. HPLC trace of **3dh**.

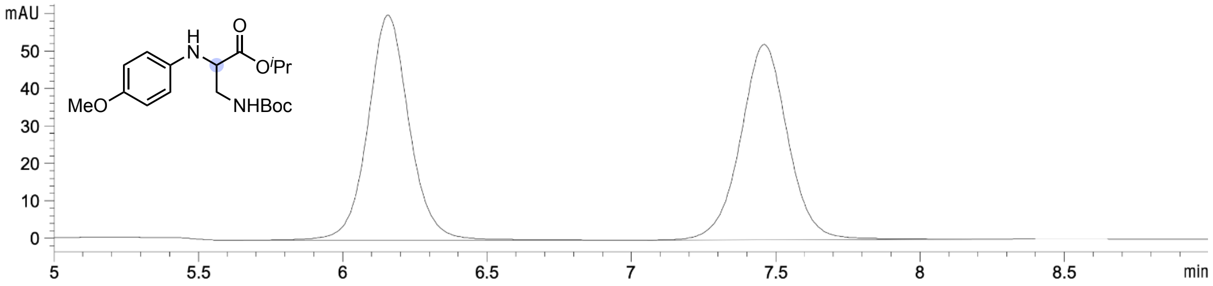

Signal 1: DAD1 A, Sig=210,4 Ref=off

Peak #	RetTime [min]	Type	Width [min]	Area [mAU*s]	Height [mAU]	Area %
1	6.159	BB	0.1524	600.01117	60.12045	50.2897
2	7.462	BB	0.1748	593.09760	52.11092	49.7103

Figure S51. HPLC trace of *rac*-**3di**.

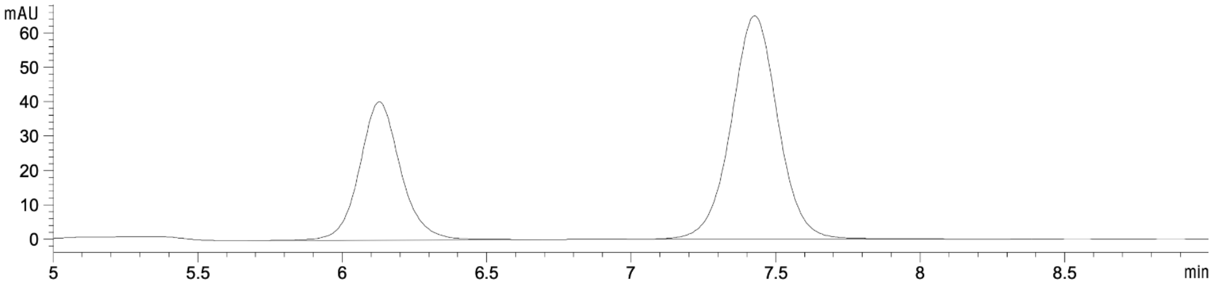

Signal 1: DAD1 A, Sig=210,4 Ref=off

Peak #	RetTime [min]	Type	Width [min]	Area [mAU*s]	Height [mAU]	Area %
1	6.127	BB	0.1503	401.55496	40.26772	35.4020
2	7.425	BB	0.1735	732.71655	65.00604	64.5980

Figure S52. HPLC trace of (*S*)-**3di**.

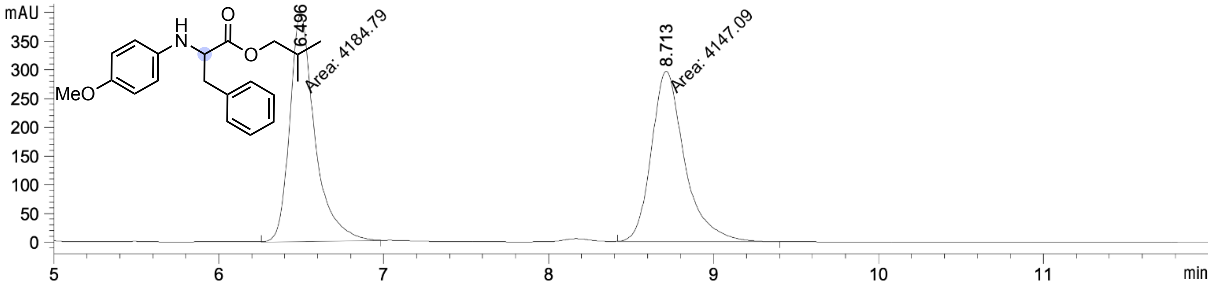

Signal 2: DAD1 B, Sig=254,4 Ref=off

Peak #	RetTime [min]	Type	Width [min]	Area [mAU*s]	Height [mAU]	Area %
1	6.496	MM	0.1766	4184.78955	395.03937	50.2262
2	8.713	MM	0.2333	4147.09424	296.29752	49.7738

Figure S53. HPLC trace of *rac*-**3ea**.

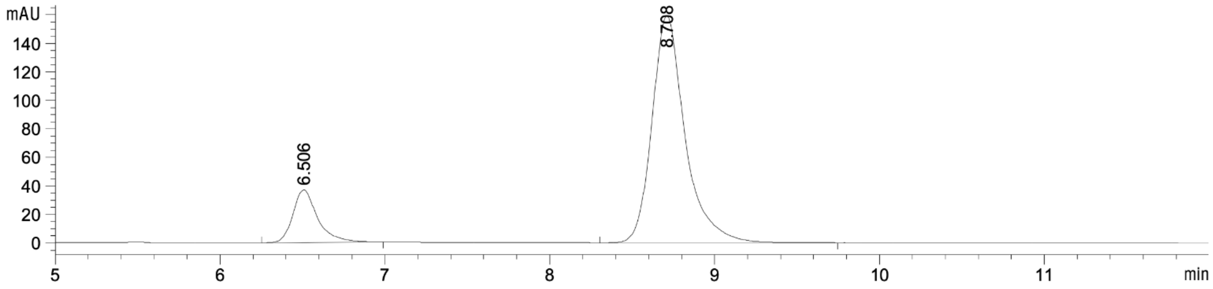

Signal 2: DAD1 B, Sig=254,4 Ref=off

Peak #	RetTime [min]	Type	Width [min]	Area [mAU*s]	Height [mAU]	Area %
1	6.506	BB	0.1559	386.47385	36.98249	14.8171
2	8.708	BB	0.2091	2221.82324	158.97409	85.1829

Figure S54. HPLC trace of (*S*)-**3ea**.

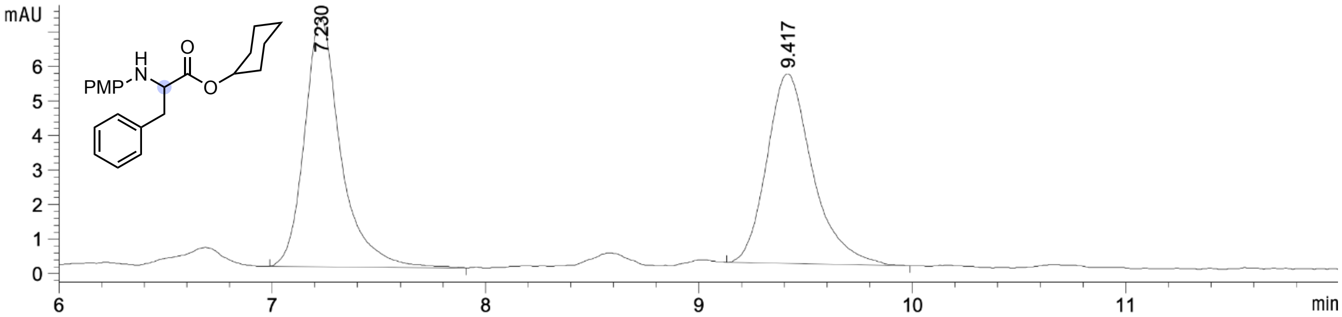

Signal 1: DAD1 A, Sig=280,4 Ref=off

Peak #	RetTime [min]	Type	Width [min]	Area [mAU*s]	Height [mAU]	Area %
1	7.230	BB	0.1754	85.05344	7.22359	50.8474
2	9.417	BB	0.2244	82.21836	5.50040	49.1526

Figure S55. HPLC trace of *rac*-**3fa**.

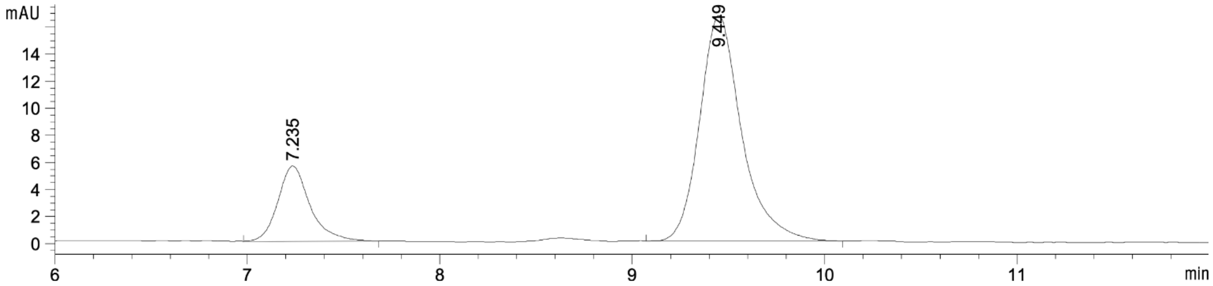

Signal 1: DAD1 A, Sig=280,4 Ref=off

Peak #	RetTime [min]	Type	Width [min]	Area [mAU*s]	Height [mAU]	Area %
1	7.235	BB	0.1732	64.62483	5.57588	20.3740
2	9.449	BB	0.2266	252.56787	16.68164	79.6260

Figure S56. HPLC trace of (*S*)-**3fa**.

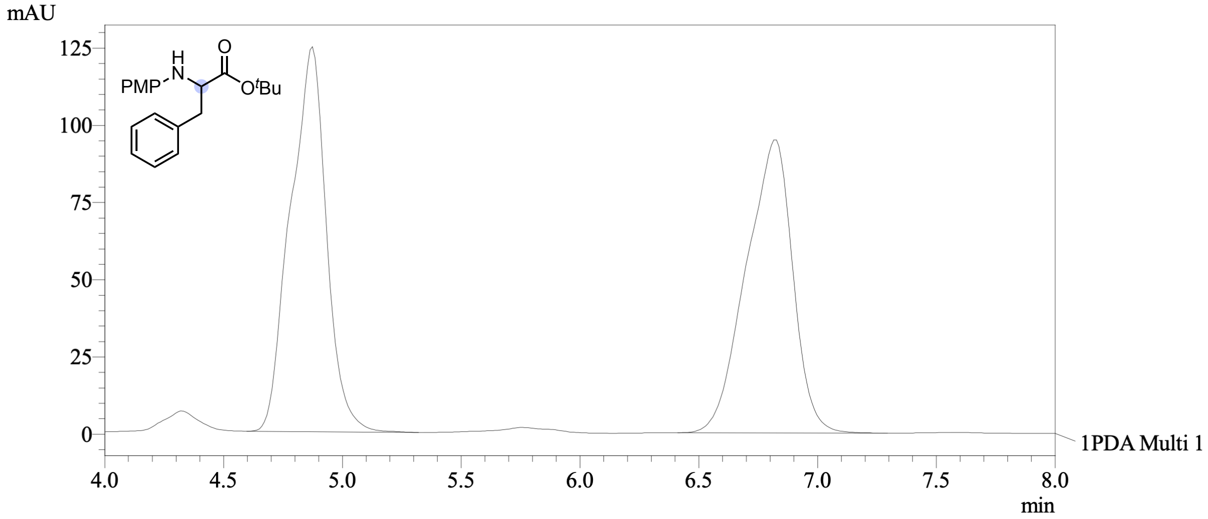

1 PDA Multi 1 / 310nm 4nm

PeakTable

PDA Ch1 310nm 4nm

Peak#	Ret. Time	Area	Height	Area %	Height %
1	4.866	1315570	124646	50.364	56.779
2	6.816	1296551	94882	49.636	43.221
Total		2612120	219527	100.000	100.000

Figure S57. HPLC trace of *rac*-**3ga**.

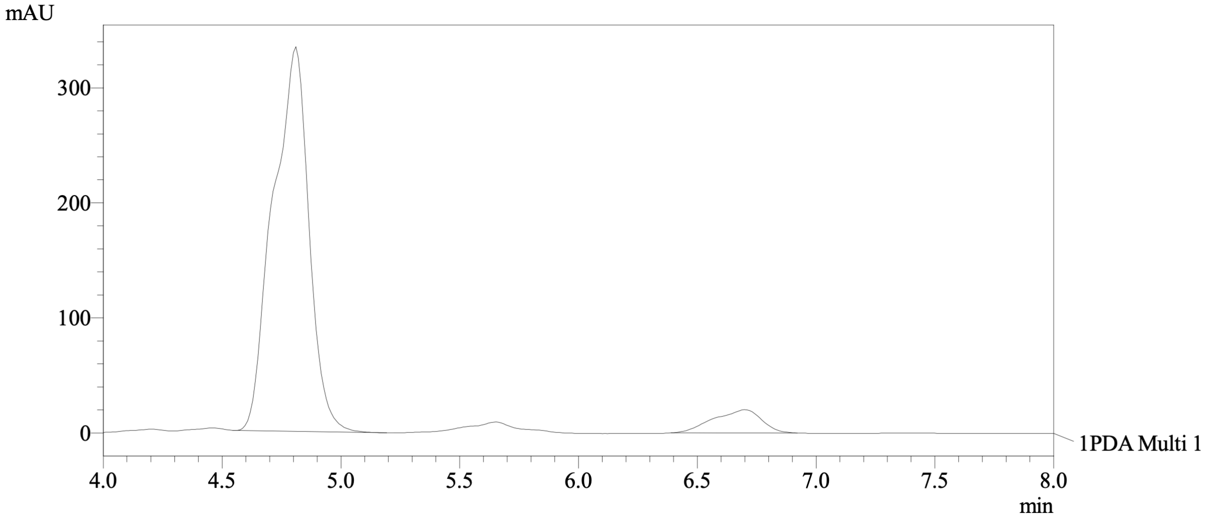

1 PDA Multi 1 / 310nm 4nm

PeakTable

PDA Ch1 310nm 4nm

Peak#	Ret. Time	Area	Height	Area %	Height %
1	4.804	3514943	334361	92.535	94.299
2	6.695	283564	20215	7.465	5.701
Total		3798507	354576	100.000	100.000

Figure S58. HPLC trace of (*R*)-**3ga**.

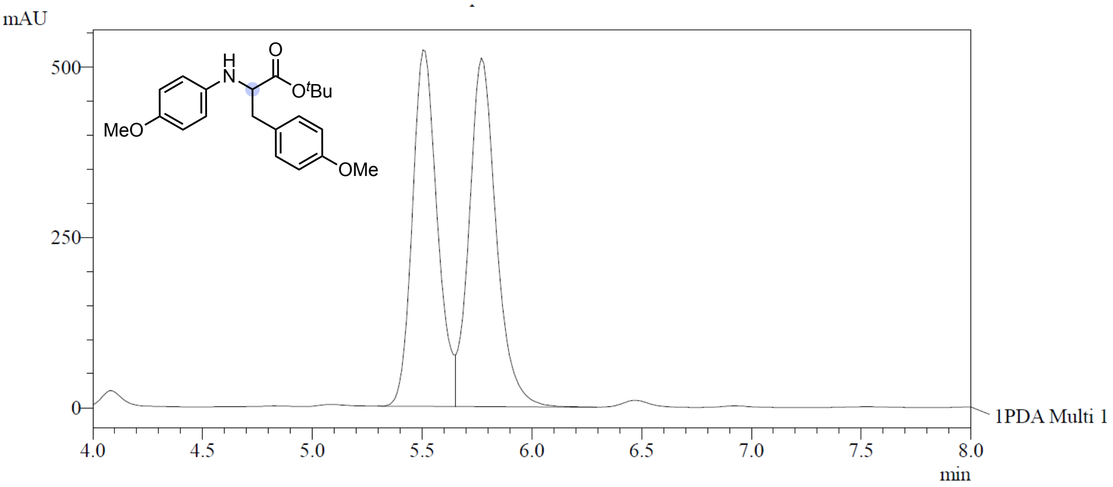

1 PDA Multi 1 / 228nm 4nm

PeakTable

PDA Ch1 228nm 4nm

Peak#	Ret. Time	Area	Height	Area %	Height %
1	5.503	4155558	523665	48.748	50.551
2	5.767	4369003	512249	51.252	49.449
Total		8524561	1035914	100.000	100.000

Figure S59. HPLC trace of *rac*-**3gb**.

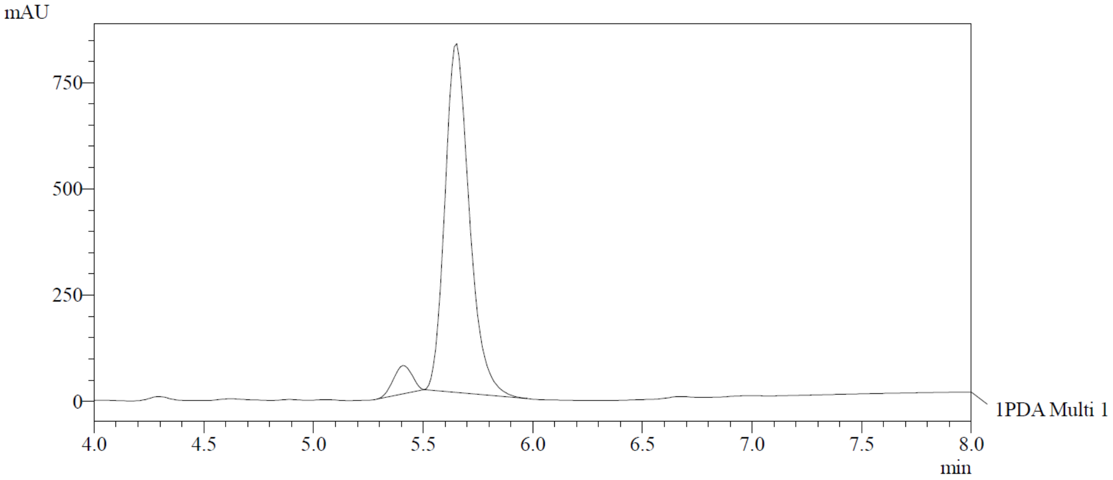

1 PDA Multi 1 / 228nm 4nm

PeakTable

PDA Ch1 228nm 4nm

Peak#	Ret. Time	Area	Height	Area %	Height %
1	5.405	404709	67049	5.958	7.555
2	5.645	6388352	820424	94.042	92.445
Total		6793060	887473	100.000	100.000

Figure S60. HPLC trace of (*S*)-**3gb**.

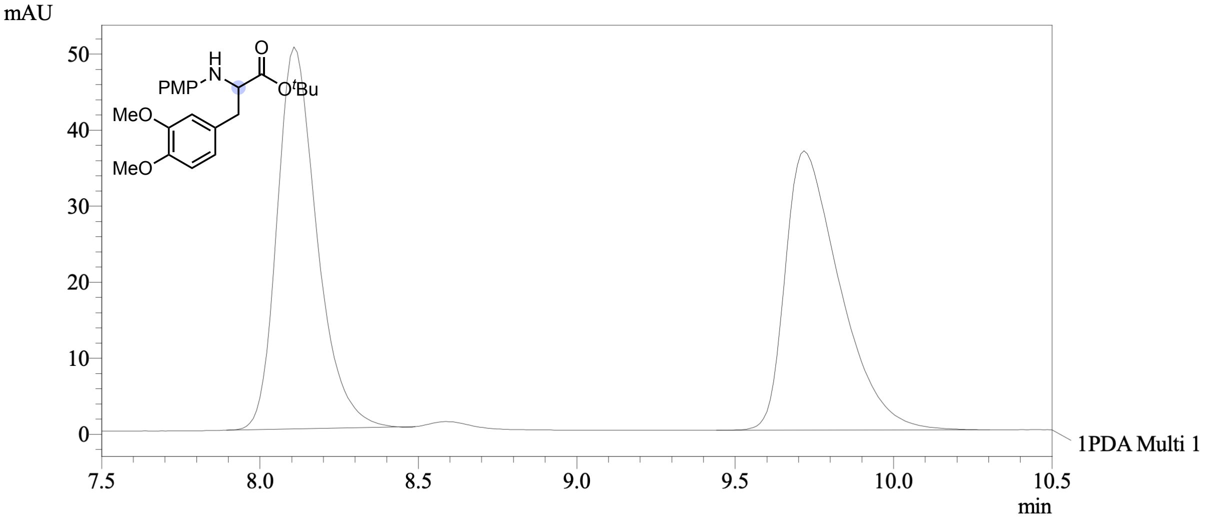

1 PDA Multi 1 / 315nm 4nm

PeakTable

PDA Ch1 315nm 4nm

Peak#	Ret. Time	Area	Height	Area %	Height %
1	8.103	440271	50252	49.931	57.761
2	9.712	441483	36748	50.069	42.239
Total		881754	87001	100.000	100.000

Figure S61. HPLC trace of *rac*-**3gc**.

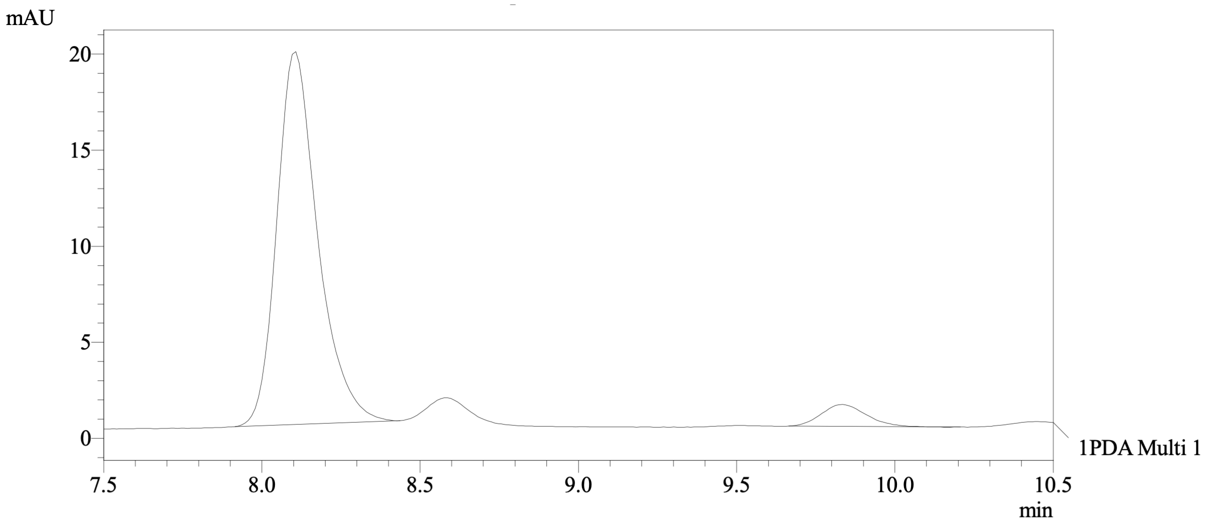

1 PDA Multi 1 / 315nm 4nm

PeakTable

PDA Ch1 315nm 4nm

Peak#	Ret. Time	Area	Height	Area %	Height %
1	8.098	174838	19406	93.921	94.425
2	9.828	11317	1146	6.079	5.575
Total		186155	20552	100.000	100.000

Figure S62. HPLC trace of (*R*)-**3gc**.

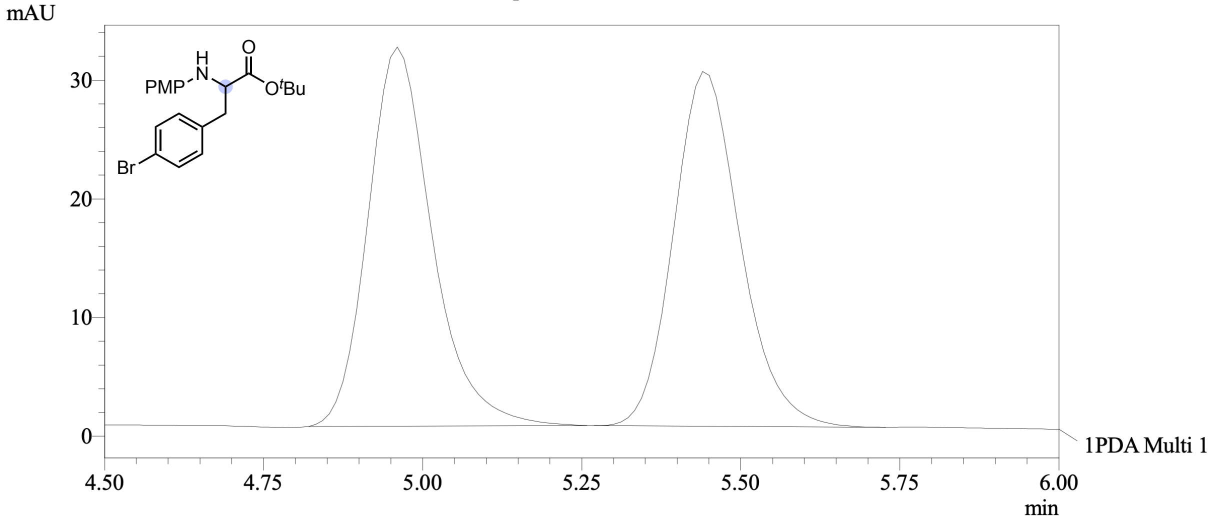

1 PDA Multi 1 / 310nm 4nm

PeakTable

PDA Ch1 310nm 4nm

Peak#	Ret. Time	Area	Height	Area %	Height %
1	4.954	223745	31935	50.626	51.657
2	5.438	218208	29887	49.374	48.343
Total		441953	61822	100.000	100.000

Figure S63. HPLC trace of *rac*-**3gd**.

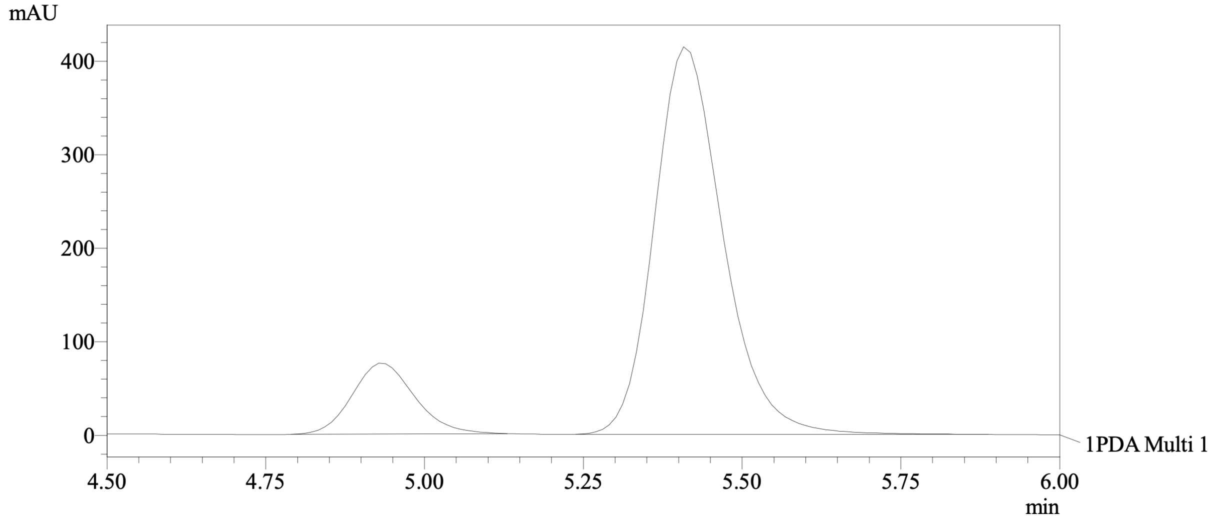

1 PDA Multi 1 / 310nm 4nm

PeakTable

PDA Ch1 310nm 4nm

Peak#	Ret. Time	Area	Height	Area %	Height %
1	4.927	519267	75871	14.284	15.477
2	5.405	3115970	414351	85.716	84.523
Total		3635237	490223	100.000	100.000

Figure S64. HPLC trace of (*S*)-**3gd**.

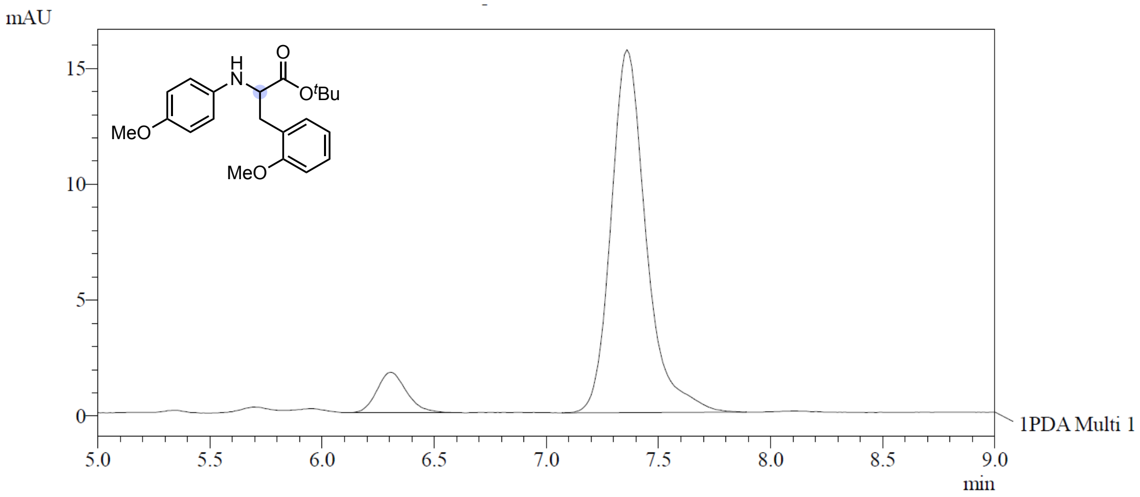

1 PDA Multi 1 / 310nm 4nm

PeakTable

PDA Ch1 310nm 4nm

Peak#	Ret. Time	Area	Height	Area %	Height %
1	6.301	16039	1744	8.611	10.017
2	7.355	170227	15663	91.389	89.983
Total		186266	17407	100.000	100.000

Figure S65. HPLC trace of (*S*)-**3ge**.

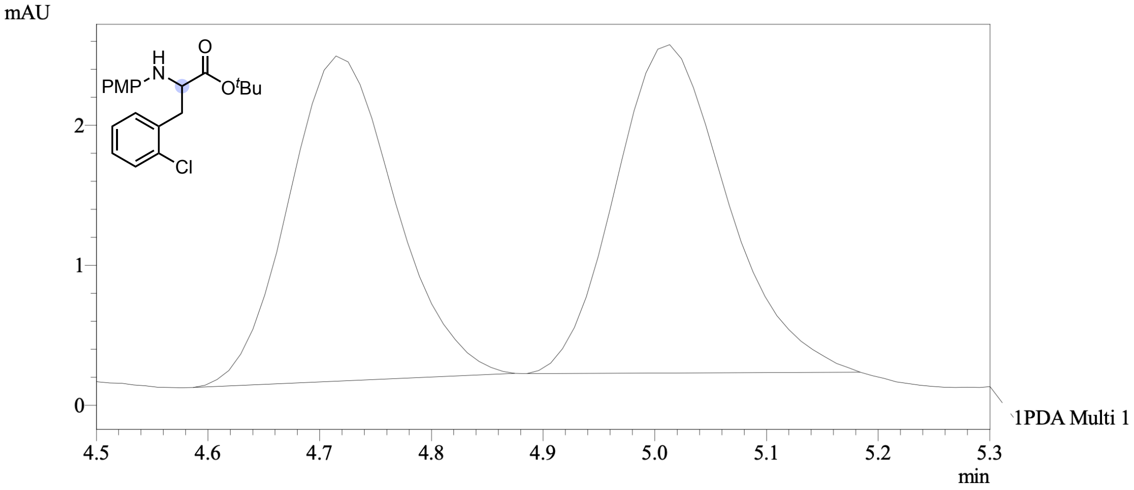

1 PDA Multi 1 / 310nm 4nm

PeakTable

PDA Ch1 310nm 4nm

Peak#	Ret. Time	Area	Height	Area %	Height %
1	4.712	15473	2323	48.591	49.773
2	5.005	16371	2345	51.409	50.227
Total		31844	4668	100.000	100.000

Figure S66. HPLC trace of *rac*-**3gf**.

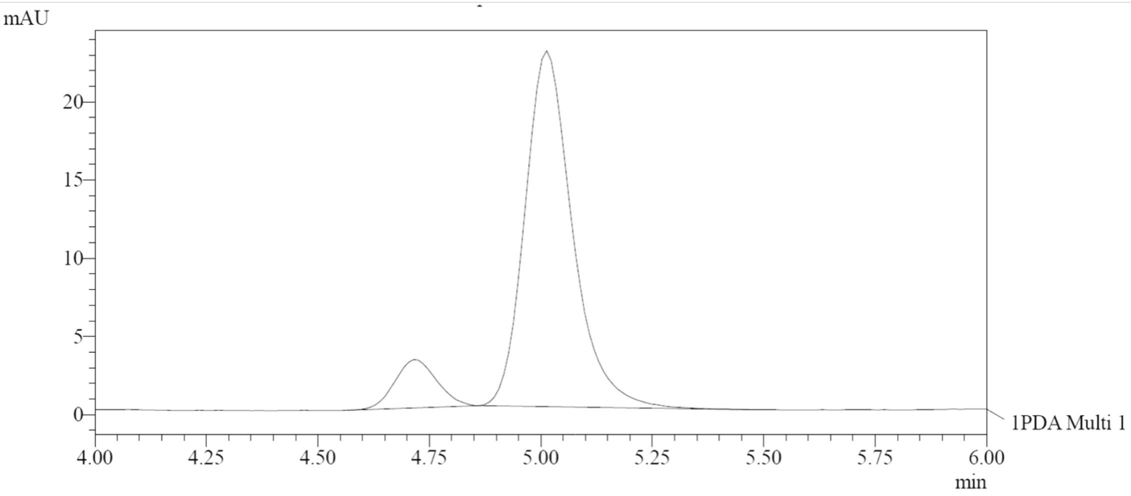

1 PDA Multi 1 / 310nm 4nm

PeakTable

PDA Ch1 310nm 4nm

Peak#	Ret. Time	Area	Height	Area %	Height %
1	4.713	20247	3087	10.702	11.942
2	5.008	168940	22762	89.298	88.058
Total		189187	25849	100.000	100.000

Figure S67. HPLC trace of (*S*)-**3gf**.

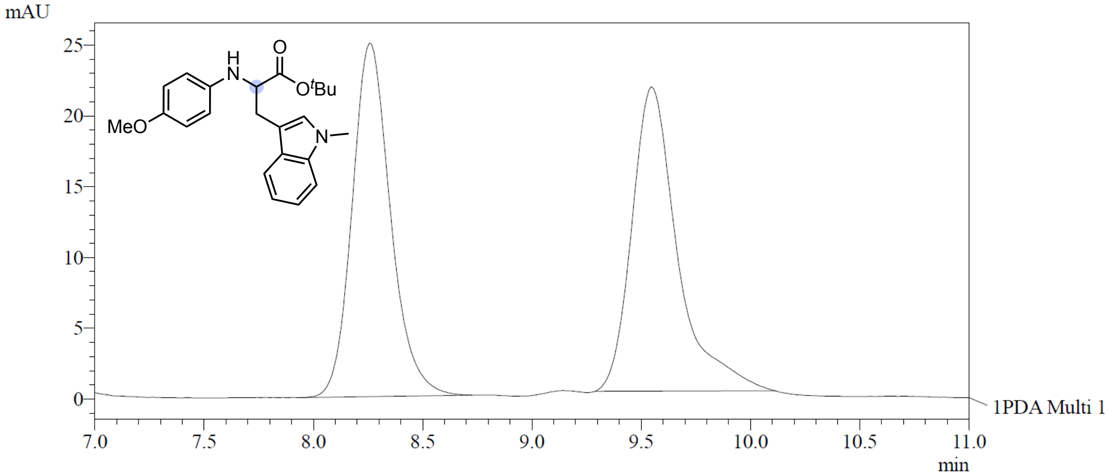

1 PDA Multi 1 / 290nm 4nm

PeakTable

PDA Ch1 290nm 4nm

Peak#	Ret. Time	Area	Height	Area %	Height %
1	8.254	305083	24971	49.448	53.751
2	9.543	311889	21486	50.552	46.249
Total		616972	46458	100.000	100.000

Figure S68. HPLC trace of *rac*-**3gg**.

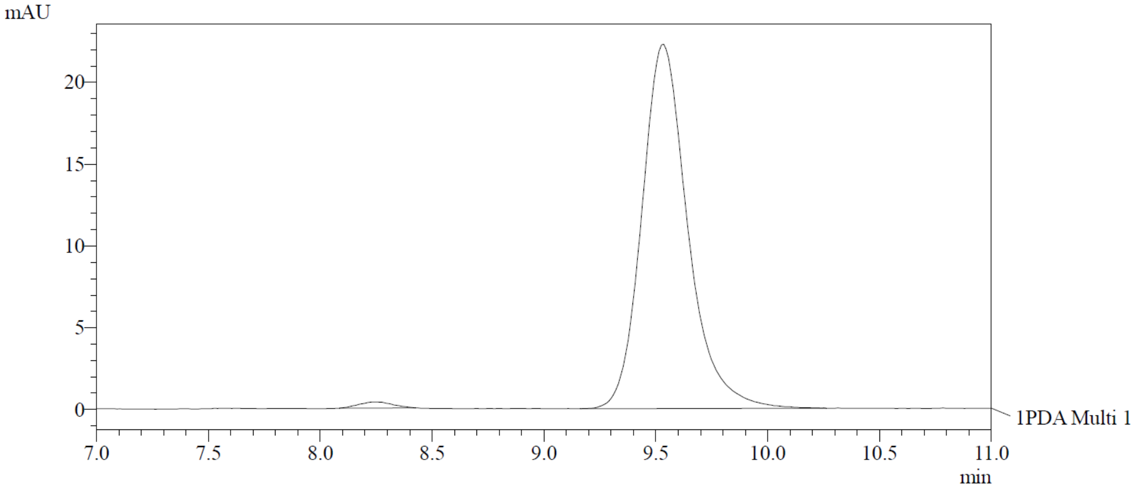

1 PDA Multi 1 / 290nm 4nm

PeakTable

PDA Ch1 290nm 4nm

Peak#	Ret. Time	Area	Height	Area %	Height %
1	8.231	3866	373	1.199	1.650
2	9.527	318478	22265	98.801	98.350
Total		322344	22639	100.000	100.000

Figure S69. HPLC trace of (*S*)-**3gg**.

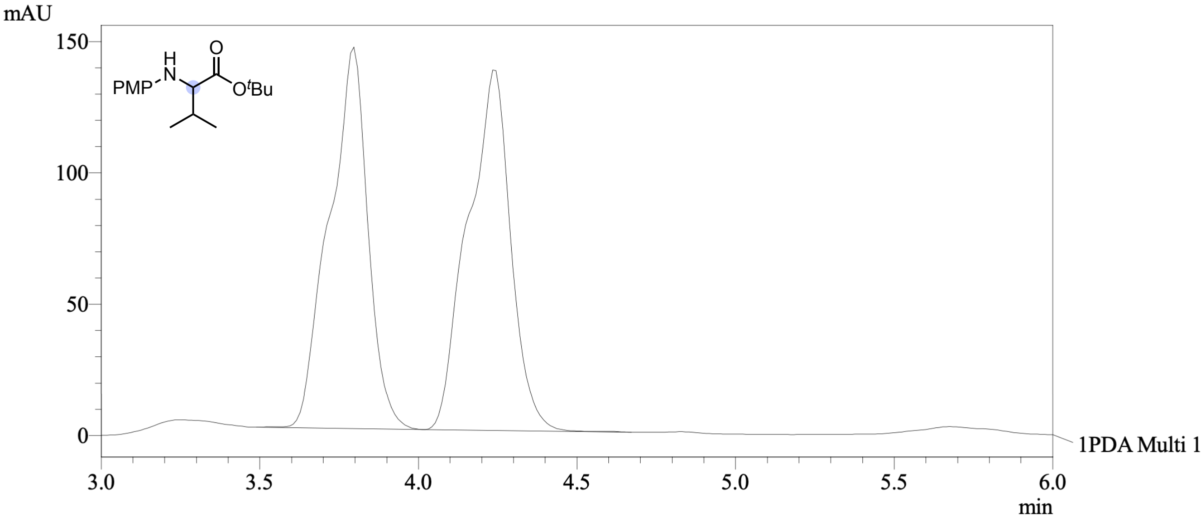

1 PDA Multi 1 / 310nm 4nm

PeakTable

PDA Ch1 310nm 4nm

Peak#	Ret. Time	Area	Height	Area %	Height %
1	3.789	1232427	145260	48.767	51.399
2	4.234	1294763	137351	51.233	48.601
Total		2527190	282611	100.000	100.000

Figure S70. HPLC trace of *rac*-3gh.

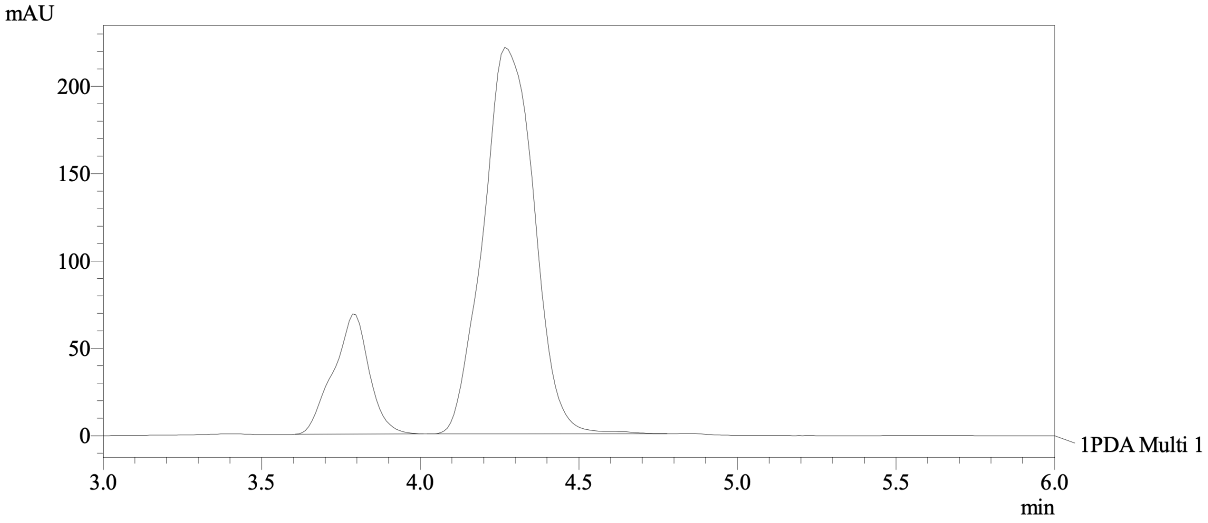

1 PDA Multi 1 / 310nm 4nm

PeakTable

PDA Ch1 310nm 4nm

Peak#	Ret. Time	Area	Height	Area %	Height %
1	3.785	542055	68917	17.903	23.745
2	4.264	2485603	221321	82.097	76.255
Total		3027658	290238	100.000	100.000

Figure S71. HPLC trace of (*S*)-3gh.

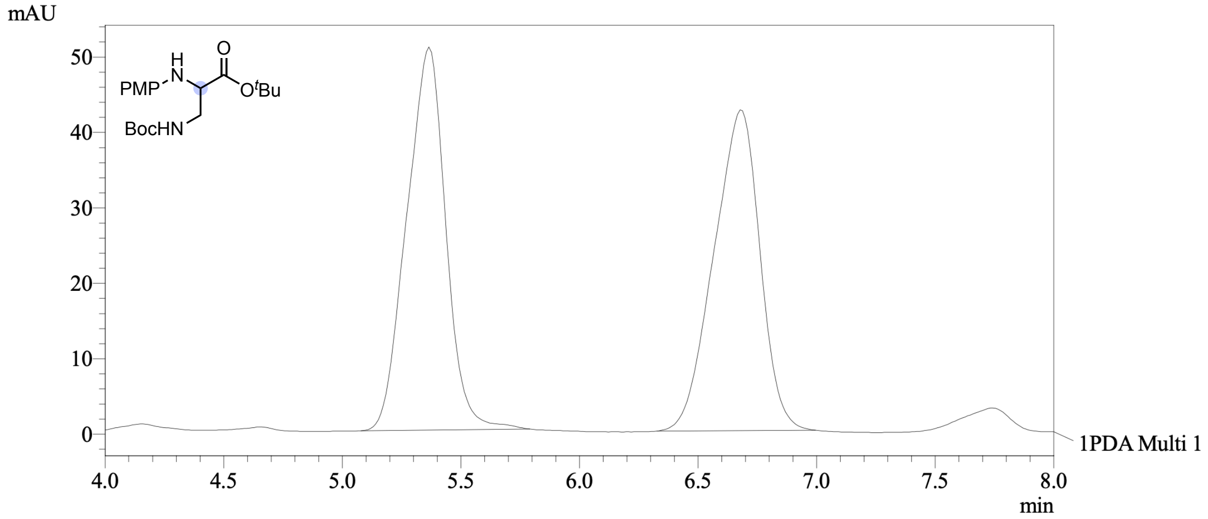

1 PDA Multi 1 / 308nm 4nm

PeakTable

PDA Ch1 308nm 4nm

Peak#	Ret. Time	Area	Height	Area %	Height %
1	5.360	583917	50816	50.312	54.425
2	6.676	576683	42553	49.688	45.575
Total		1160601	93369	100.000	100.000

Figure S72. HPLC trace of *rac*-**3gi**.

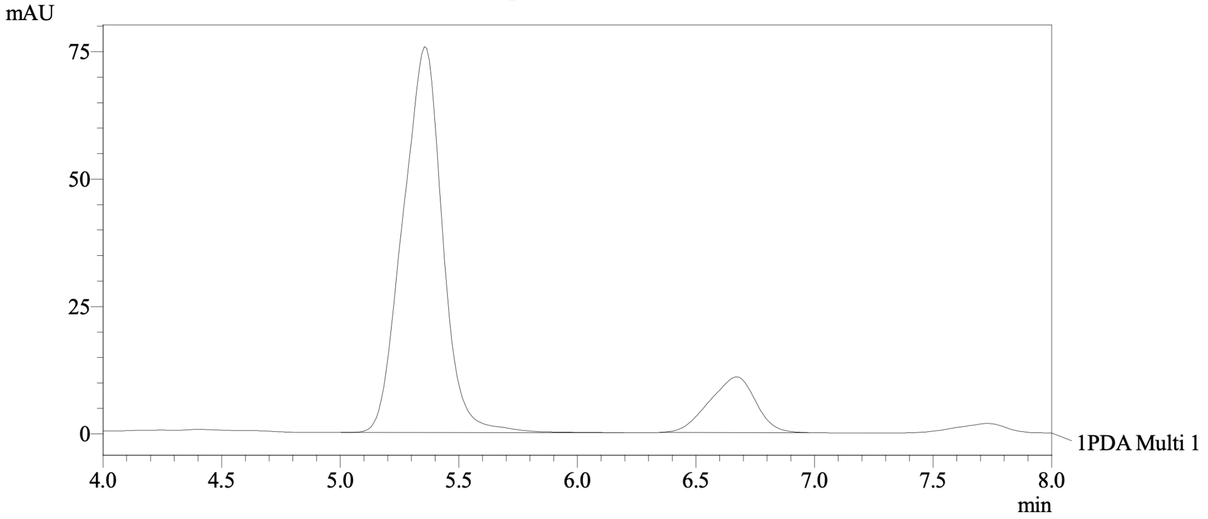

1 PDA Multi 1 / 308nm 4nm

PeakTable

PDA Ch1 308nm 4nm

Peak#	Ret. Time	Area	Height	Area %	Height %
1	5.353	879510	75734	85.594	87.383
2	6.667	148025	10935	14.406	12.617
Total		1027535	86669	100.000	100.000

Figure S73. HPLC trace of (*S*)-**3gi**.
